# Zn-N_x_ sites on N-doped carbon for aerobic oxidative cleavage and esterification of C(CO)-C bonds

**DOI:** 10.1038/s41467-021-25118-0

**Published:** 2021-08-10

**Authors:** Chao Xie, Longfei Lin, Liang Huang, Zixin Wang, Zhiwei Jiang, Zehui Zhang, Buxing Han

**Affiliations:** 1 Key Laboratory of Catalysis and Energy Materials Chemistry of Ministry of Education & Hubei Key Laboratory of Catalysis and Materials Science, South-Central University for Nationalities, Wuhan, China; 2 Beijing National Laboratory for Molecular Sciences, CAS Key Laboratory of Colloid, Interface and Chemical Thermodynamics, Institute of Chemistry, Chinese Academy of Sciences, Beijing, China; 3 The State Key Laboratory of Refractories and Metallurgy, Wuhan University of Science and Technology, Wuhan, China

**Keywords:** Heterogeneous catalysis, Chemical engineering, Sustainability

## Abstract

Selective cleavage of C-C bonds is very important in organic chemistry, but remains challenging because of their inert chemical nature. Herein, we report that Zn/NC-X catalysts, in which Zn^2+^ coordinate with N species on microporous N-doped carbon (NC) and X denotes the pyrolysis temperature, can effectively catalyze aerobic oxidative cleavage of C(CO)-C bonds and quantitatively convert acetophenone to methyl benzoate with a yield of 99% at 100 °C. The Zn/NC-950 can be applied for a wide scope of acetophenone derivatives as well as more challenging alkyl ketones. Detail mechanistic investigations reveal that the catalytic performance of Zn/NC-950 can be attributed to the coordination between Zn^2+^ and N species to change the electronic state of the metal, synergetic effect of the Zn single sites with their surrounding N atoms, as well as the microporous structure with the high surface area and structural defects of the NC.

## Abstract (web summary)

Selective cleavage of C-C bonds is very important in organic chemistry, but remains challenging because of their inert chemical nature. Here, the authors report that Zn^2+^ coordinated with N species on N-doped carbon catalysts can catalyze the aerobic oxidative cleavage and esterification of C(CO)-C bonds with high efficiency and activity.

## Introduction

The selective cleavage of C–C bonds is an attractive but challenging topic in organic chemistry^1–9^. It enables a straightforward reconstruction strategy of carbon skeletons that could hardly be achieved by other means. Ketones are a class of versatile compounds that can be easily obtained from fossil resources or biomass. Through C(CO)–C bond cleavage, the ketones can be converted into acids^10^, esters^11^, amides^12,13^, nitriles^14^, ketones^15–17^, and acyl-metal complexes^18^. Thus, the selective cleavage and functionalization of C(CO)–C bond is of crucial importance for the conversion of ketones into value-added products.

To date, numerous catalytic strategies have been developed for the cleavage and functionalization of C(CO)–C bond. The pre-functionalization of the *α*-C–H in ketones as the directing group is one of the efficient protocols for C(CO)–C bond cleavage^19–21^. However, the protocol is only available for specific substrates and the pre-functionalization steps are tedious, which limit its wide application. Therefore, researchers have been focusing on the direct cleavage and functionalization of C(CO)–C bond. For example, selective cleavage of C(CO)–C bond in methyl ketones to esters was carried out by the use of CuCl with air as the oxidant^11^. Besides CuCl, CuBr^22^, CuI^23^, CuCl_2_^12,24^, Cu(NO_3_)_2_^10^, and Cu(OAc)_2_^14^ were also used for the C(CO)–C bond cleavage. Despite the great achievements that have been made, the need for substantial amounts of Cu salts and additives, and the difficulty in recovery limit their applications. Heterogeneous catalyst such as Co nanoparticles was also reported for catalyzing the C–C bond cleavage and esterification in C(OH)–C via C(CO)–C intermediate^25^. However, the state-of-the-art catalysts have low activity for oxidative cleavage of the inert C(CO)–C bond at mild conditions (Supplementary Table 1, TOF ≤0.6 h^−1^). Therefore, the design of heterogeneous catalytic systems to effectively catalyze aerobic oxidative cleavage of C(CO)–C bonds under mild conditions is still highly desirable.

Zinc is one of the most abundant, safest, and cheapest materials available. Zinc salts have been used as efficient catalysts for some organic transformation^26–28^, and their catalytic performance could be easily adjusted by changing the coordination environment^29^. However, the conventional Zinc catalysts were usually used as Lewis acid for the electron-rich groups activation, such as alkyne and carbonyl groups^30^. They suffered from limited substrate scopes, and also intrinsic difficulty in product separation and catalyst recycling. Therefore, it is highly desired to seek heterogeneous zinc catalysts with very active sites, and broad substrate scope. Recently, two elegant works on Zn coordination catalyst for organic transformation have been developed^31,32^. Li et al. reported that Zn(II) coordinated with a bipyridine-based metal-organic framework could be used for the intramolecular hydroamination of *o*-alkynylanilines^31^. Yang et al. reported that hollow N-doped porous carbon with ultrahigh concentrations of single Zn sites could be used for the CO_2_ cycloaddition reactions^32^. However, the Zn-based catalysts have not been reported for catalyzing the oxidative cleavage of C(CO)–C bond.

Carbon‐based materials play an important role in energy and catalytic applications^33–35^. Their excellent design flexibility enables us to fabricate a variety of materials with unique properties^36–40^. Herein, we fabricated Zn-based heterogeneous catalysts (Zn/NC-X), in which Zn single sites [Zn^δ+^ (0 < *δ* < 2)] were anchored on microporous N-doped carbon (NC). It was found that the designed Zn/NC-950 catalyst showed good catalytic performance for aerobic oxidative cleavage and esterification of C(CO)–C bonds of ketones, and the activity with TOF of 7.5 h^−1^ for conversion of acetophenone to methyl benzoate at 100 °C was obtained (Supplementary Table 1).

## Results

### Synthesis and characterization

**Fig. 1:**
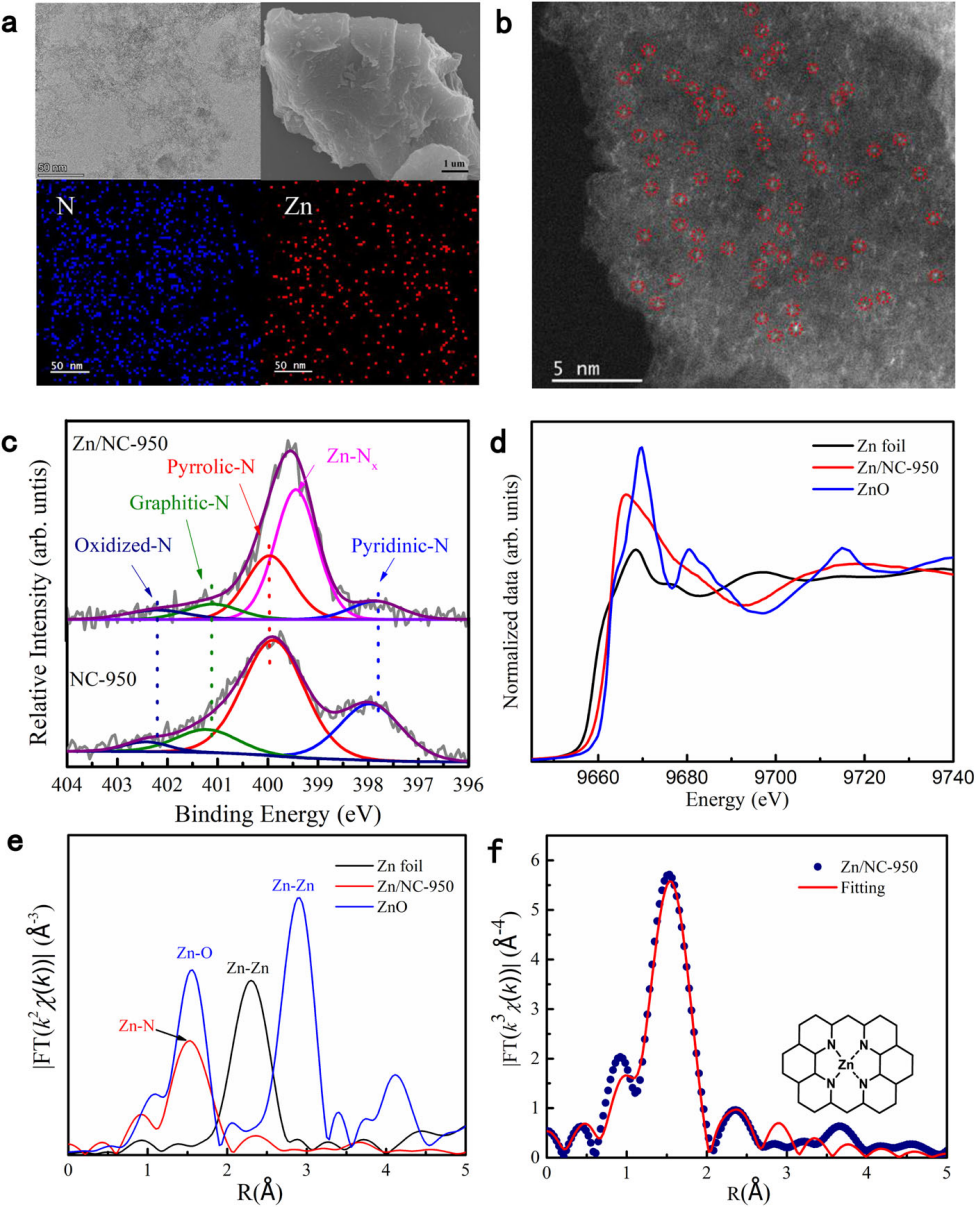
Characterization of the synthesized Zn/NC-950. **a** STEM, SEM images, and elemental mapping of N and Zn of Zn/NC-950 catalyst. **b** Atomic-resolution HAADF-STEM image of Zn/NC-950 catalyst, in which the bright dots are Zn centers, some of which are highlighted by red circles. **c** N 1*s* XPS spectra for Zn/NC-950 and NC-950. **d** XANES, **e** FT-EXAFS spectra, and **f** FT-EXAFS fitting curve for Zn/NC-950 at the Zn K-edge. The inset shows the structure model of a Zn site in Zn/NC-950.

The Zn/NC-X catalysts were prepared by the pyrolysis of zinc chloride/chitosan-composites (Supplementary Fig. 1). A representative material Zn/NC-950 was systematically studied. The Zn/NC-950 exhibits microporous structures with BET surface areas of 1678 m^2^ g^−1^ (Supplementary Fig. 2a). Powder X-ray diffraction (XRD) patterns of Zn/NC-950 showed two broaden peaks at ~25^°^ and ~44^°^, which could be assigned to (002) and (101) planes of the graphitic carbon, and no characteristic peaks corresponding to the metallic Zn or ZnO species were observed (Supplementary Fig. 2b). Zn/NC-950 catalyst was composed of amorphous flakes as shown in STEM and SEM images (Fig. 1a) and site-isolated Zn centers were dispersed over the entire flakes confirmed by HAADF-STEM and EDS mapping (Fig. 1a, b). A loading of 0.75 wt% Zn was identified for Zn/NC-950 by inductively coupled plasma atomic emission spectroscopy (ICP-AES). The N 1*s* in Zn/NC-950 was characterized by high-resolution X-ray photoelectron spectroscopy (XPS) (Fig. 1c), and a new peak located in 399.4 eV was observed, indicating some N species were coordinated with the Zn sites^41^. The XPS position shifts in Zn/NC-950 compared with NC-950 can be ascribed to the local environment change caused by the coordination of Zn centers with N species.

The Zn/NC-950 was further characterized by Zn K-edge synchrotron X-ray absorption spectroscopy. The absorption edge position for Zn/NC-950 from X-ray absorption near-edge spectra is neither consistent with the Zn foil nor ZnO, but the absorption threshold is closed to that of ZnO (Fig. 1d), indicating that the valance state of zinc species in Zn/NC-950 was between 0 and +2 (close to +2) which shows that the Zn^2+^ ions coordinate with the N species in the catalyst. The high-resolution XPS spectrum of Zn 2*p* was also conducted. It shows two peaks at 1021.7 and 1044.8 eV, corresponding to the 2*p*_3/2_ and 2*p*_1/2_ electronic states of Zn species, respectively (Supplementary Fig. 2c). The lower binding energy of 2*p*_3/2_ (1021.7 eV) of Zn species in Zn/NC-950 catalyst than that of the standard ZnO (1022 eV) further confirms that the Zn–N_x_ is the predominant active component in Zn/NC-950 catalyst rather than ZnO or metal Zn^0^. The *k*^2^-weighted Fourier transform (FT) of the extended X-ray absorption fine structure spectra (EXAFS) illustrates that the Zn/NC-950 exhibits an obvious peak located at 1.53 Å, which can be attributed to the Zn–N coordination^41^, and small scattering peaks derived from Zn–Zn coordination were observed (Fig. 1e), indicating the existence of a small number of Zn clusters or nanoparticles. The fitted EXAFS data reveals that the ZnN_4_ structure formed through the coordination of Zn^2+^ ions with N species in Zn/NC-950 is the predominant active component (Fig. 1f, Supplementary Table 2).

### Catalytic tests

The catalytic activities of the prepared materials were tested for the aerobic oxidative esterification of acetophenone with methanol (Table 1). No reaction occurred without any catalysts (Table 1, Entry 1). Among all pyrolysis products, the synthesized Zn/NC-950 afforded the best catalytic performance (Table 1, Entries 2–5). In addition, the acetophenone could be fully converted into methyl benzoate with a product yield of 99% over Zn/NC-950 in 12 h (Table 1, Entry 6). As expected, no target product was formed under nitrogen atmosphere (Table 1, Entry 7), indicating that O_2_ was essential for this transformation. The poisoning experiment was performed with KSCN as binding molecule, which would interrupt the metal-centered active site and inhibit the catalyst toward the reaction^25^ (Table 1, Entry 8). 2 equiv. of KSCN was mixed with the Zn/NC-950 catalyst in methanol at 100 °C for 1 h, and then the reaction was conducted under standard conditions for 6 h. The significant decrease of the conversion of acetophenone and the yield of methyl benzoate (Table 1, Entries 4 vs 8) indicates the essential role of Zn–N_4_ structure in the catalyst for this reaction. To better distinguish the role of isolated Zn species and Zn clusters or nanoparticles, the Zn/AC-900 without the ZnN_4_ sites (derived from the pyrolysis of MOF-74 at 900 °C) was prepared, the result clearly showed that Zn nanoparticles or clusters were not the active centers for this reaction (Table 1, Entry 9). In addition, the accessible Zn clusters or nanoparticles were completely removed by acid treatment to give Zn/NC-950-H (0.62 wt% Zn content) for a control study, 52% yield of methyl benzoate was obtained (Table 1, Entry 10), indicating that the Zn single sites were responsible for the high catalytic performance, and the decreased content of the accessible Zn single sites caused by the acid treatment was the main reason for the decrease of the methyl benzoate yield. Besides, the Co/NC-950 and Cu/NC-950 were also prepared, and their catalytic activity was very low (Table 1, Entries 11–12), further confirmed the unique role of ZnN_4_ sites. In addition, control experiments were performed using N-doped carbon, Zn-phthalocyanine, and N-doped carbon combined with ZnCl_2_ as catalysts, respectively (Table 1, Entries 13–15), and these catalytic systems only afford trace amount of the target product. These results show that the coordination between Zn^2+^ and N species in the NC materials was crucial for the high activity. In other words, the activity of Zn is successfully excited for aerobic oxidative cleavage of C(CO)–C bonds by the coordination.

**Table 1:** Aerobic oxidative cleavage and esterification of acetophenone with methanol over different catalysts^a^.


Entry	Catalyst	Time/h	Conversion/%	Yield/%
1	None	6	N.D.	N.D
2	Zn/NC-800	6	43	40
3	Zn/NC-900	6	60	58
4	Zn/NC-950	6	85	83
5	Zn/NC-1000	6	77	76
6	Zn/NC-950	12	>99	99
7^b^	Zn/NC-950	6	N.D.	N.D.
8^c^	Zn/NC-950 + KSCN	6	32	28
9	Zn/AC-900	6	Trace	Trace
10	Zn/NC-950-H	6	57	52
11	Co/NC-950	6	5	2
12	Cu/NC-950	6	4	Trace
13	NC-950	6	Trace	Trace
14	Zn-phthalocyanine	6	Trace	Trace
15^d^	NC-950 + ZnCl_2_	6	Trace	Trace

^a^Reaction conditions: acetophenone (0.5 mmol), ethylbenzene (internal standard, 0.5 mmol), catalyst (50 mg), methanol (10 mL), O_2_ (5 atm), 100 °C.

^b^5 atm. N_2_.

^c^1 mmol KSCN was added.

^d^0.1 mmol ZnCl_2_ was added. N.D., not detected.

The heterogeneous nature of Zn/NC-950 was evaluated by removing the catalyst after the reaction was conducted for 2 h, and then the reaction was continued for 4 and 10 h. The product yield did not further increase after removal of Zn/NC-950 (Supplementary Fig. 3c), indicating no leaching of active species into the reaction mixture. Meanwhile, the concentrations of Zn in the reaction solution are below the detection limit of ICP-AES, further confirmed the negligible leaching of Zn species and the heterogeneous nature of Zn/NC-950. Notably, the activity of Zn/NC-950 as a heterogeneous catalyst is an order of magnitude higher than state-of-the-art catalysts including homogeneous catalysts (Supplementary Table 1, TOF 7.5 vs 0.2–0.6), and the C(CO)–C bond cleavage of acetophenone can occur even at 70 °C with TOF of 3.8 h^−1^ on Zn/NC-950 (Supplementary Fig. 3a and Supplementary Table 1), which was barely reported under such mild conditions. The Zn/NC-950 catalyst could be recycled for five times without considerable decrease in activity (Supplementary Fig. 3d). Moreover, no obvious change was observed between the virgin and recovered Zn/NC-950 as characterized by TEM, XRD, and XPS (Supplementary Fig. 4, Supplementary Table 3), and the content of the main active component of Zn–N_x_ calculated from N 1*s* XPS shows negligible variation, which is consistent with the observed stable catalytic performance.

The catalytic performance of Zn/NC-950 was also investigated for conversion of various ketones with alcohols. A series of linear aliphatic alcohols including ethanol, 1-propanol, and 1-butanol undergo the oxidative cleavage of acetophenone into the corresponding esters in good yields with the assistance of K_2_CO_3_, and a gradual decrease in product yield was observed when the carbon chain length of the aliphatic alcohol increased (Table 2, 1a–1d). The present catalytic system is also applicable to the oxidative esterification of branched alcohols (Table 2, 1e). The scope of the presented methodology was then extended to the oxidative esterification of a series of structurally diverse (het)aryl methyl ketones under similar conditions. Various (het)aryl methyl ketones were selectively converted into the corresponding methyl esters in excellent yields. The methoxy group at *ortho* positions shows a lower reactivity to the desired ester in comparison with that in the *para* and *meta* position (Table 2, 1f–1h), indicating that the efficiency is affected by the position of the substituents. It was observed that the (het)aryl methyl ketones substituted with various electron-withdrawing and electron donating groups all reacted with methanol smoothly, giving the desired esters in >90% yields, and a wide range of functional groups, including CN-, halo-, CH_3_SO_2_-, NO_2_-, and CF_3_- groups, are all tolerant in this transformation (Table 2, 1i–1q). In addition, 4-acetyl-biphenyl and 2-acetonaphthone also work well, giving corresponding esters in 96% and 97% yields, respectively (Table 2, 1r–1s). It is worth noting that various heteroaryl methyl ketones, including 1,3-benzodioxole, 2,3-dihydrobenzo[b][1,4]dioxine, benzofuran, pyridine, thiophene, and furan fragments, are usually in low yields and poor selectivity^23,25^, but all of them can be successfully converted into corresponding heterocyclic carboxylic acid esters with yields over 90% over Zn/NC-950 (Table 2, 1t–1y).

**Table 2:**
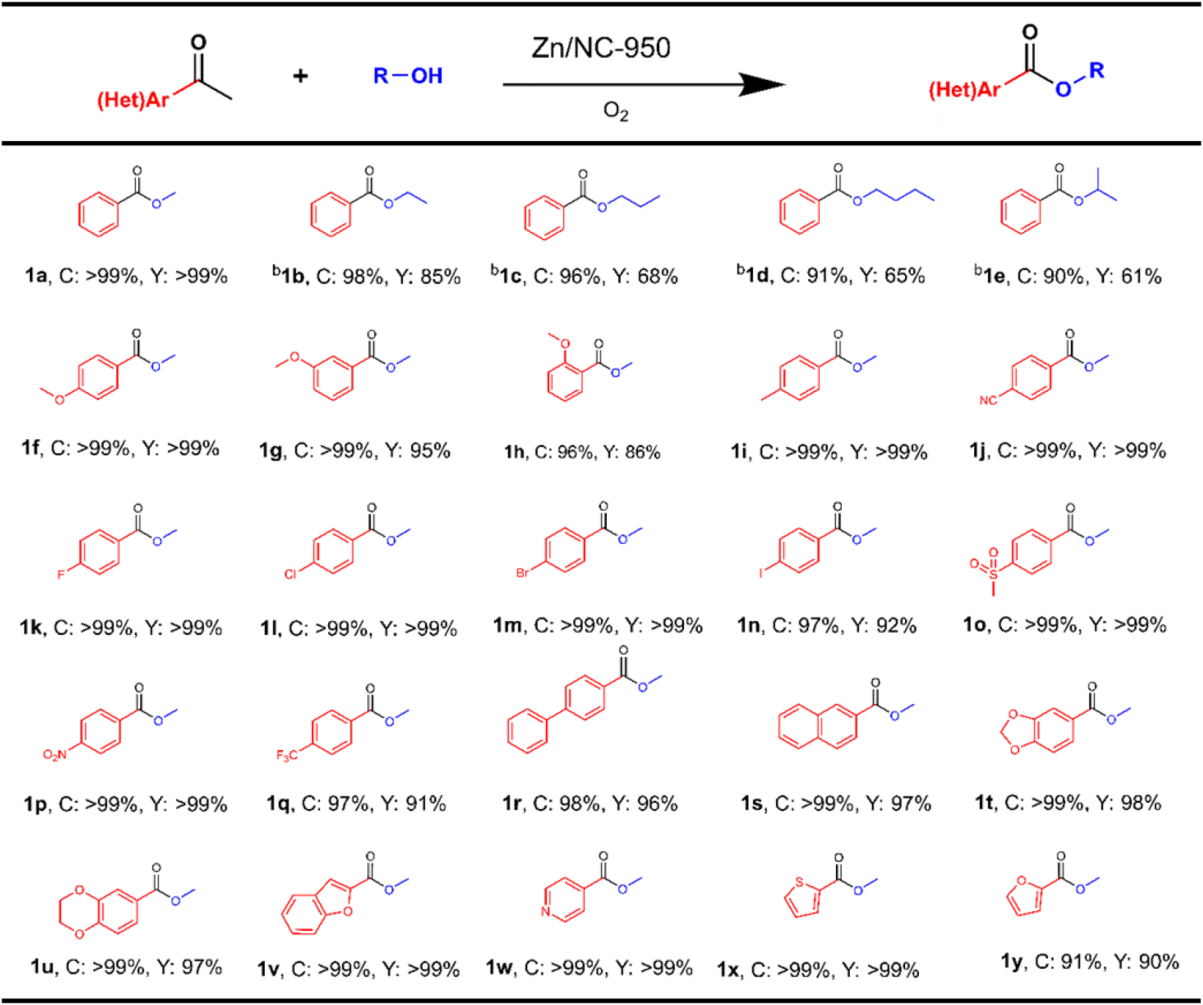
Chemoselective oxidative cleavage and esterification of C(CO)–C bonds in various (het)aryl methyl ketones with alcohols^a^.

^a^Reaction conditions: substrate (0.5 mmol), ethylbenzene (0.5 mmol), Zn/NC-950 (50 mg), methanol (10 mL), O_2_ (5 atm), 100 °C, 12 h.

^b^Substrate (0.5 mmol), ethylbenzene (0.5 mmol), Zn/NC-950 (50 mg), alcohol (10 mL), K_2_CO_3_ (0.2 mmol), O_2_ (5 atm), 120 °C, 12 h.

The capacity of the Zn/NC-950 catalyst for more challenging inactive alkyl ketones was also tested. Remarkably, the aryl ketones with long-chain alkyl groups and aliphatic ketone can also be cleaved into methyl benzoate smoothly at higher temperature with the addition of K_2_CO_3_ (Table 3, Entries 1–3). The alkyl group in heptanophenone was first cleaved into the corresponding aldehyde, acetal, and ester, then oxidatively cleaved into shorter chain aldehydes, acetals, and esters, giving CO_2_ as the final product, as convinced by the GC-MS chromatography and lime-water test (Supplementary Figs. 5–6), indicating that a successive C–C bond cleavage of the alkyl groups occurred. When 1-Indanone was used as the reactant, dimethyl phthalate was obtained as the main product (Table 3, Entry 4). Besides, the lignin model compound 2-phenoxyacetophenone was also examined, and a 97% yield of methyl benzoate was obtained. Importantly, the alkyl ketones with C=C bond were also tested, and excellent yield was obtained without cleavage of the C=C bond (Table 3, Entries 6 and 7), further confirming the superior catalytic performance of the Zn/NC-950 catalyst. In addition, *α*-ionone and *β*-ionone, as important spices, also undergo C–C bond cleavage to provide corresponding methyl ester with excellent yields (Table 3, Entries 8–9).

**Table 3:**
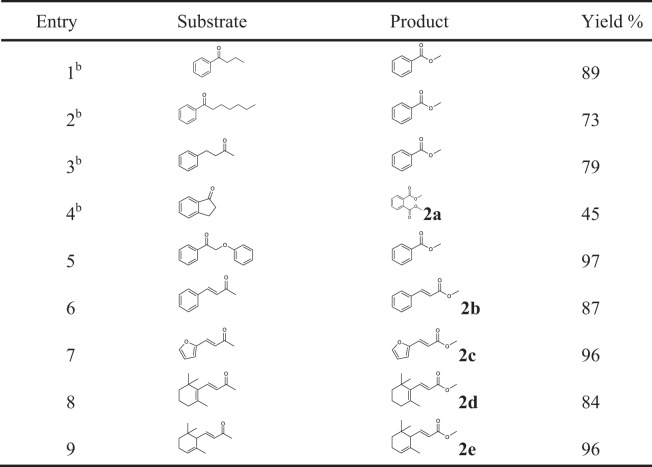
Chemoselective oxidative cleavage of C–C bonds with various alkyl ketones^a^.

^a^Reaction conditions: substrate (0.5 mmol), ethylbenzene (0.5 mmol), Zn/NC-950 (50 mg), methanol (10 mL), O_2_ (5 atm), 100 °C, 12 h.

^b^Substrate (0.5 mmol), ethylbenzene (0.5 mmol), Zn/NC-950 (50 mg), methanol (10 mL), K_2_CO_3_ (0.2 mmol), O_2_ (5 atm), 120 °C, 12 h.

### Determination of active site

**Fig. 2:**
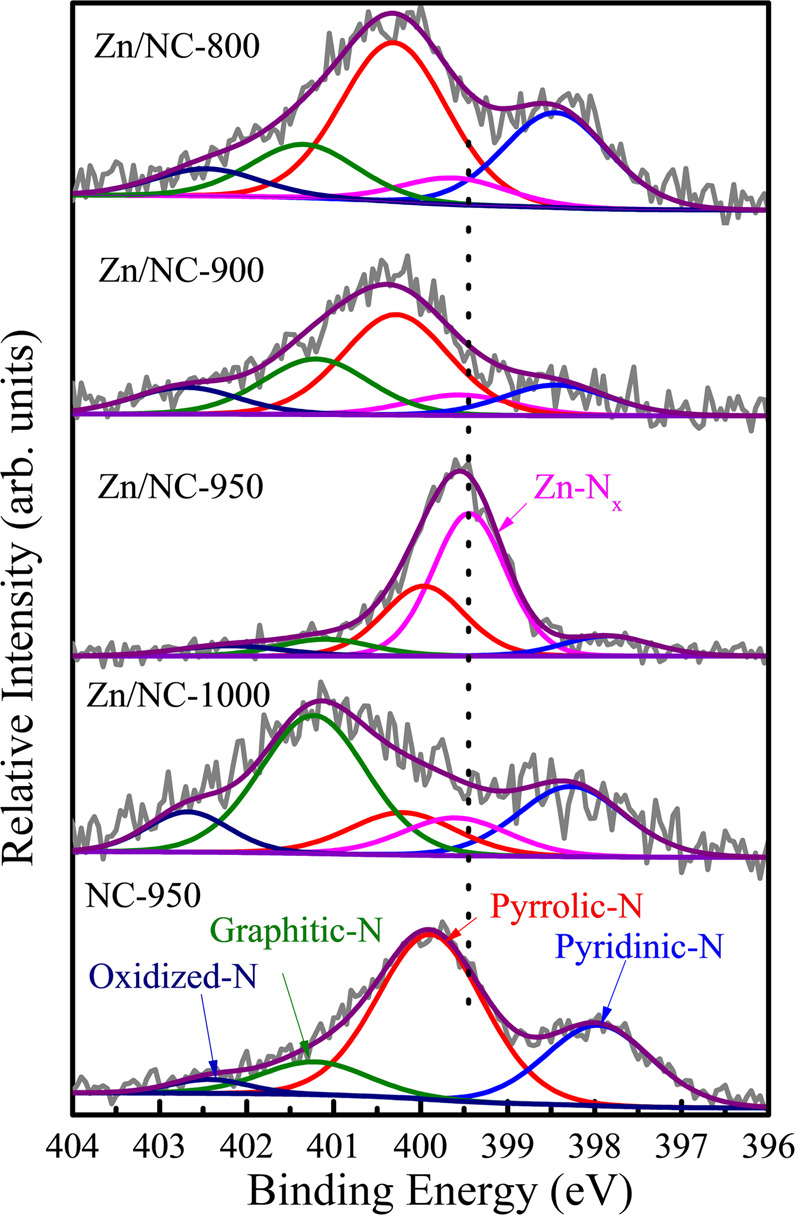
The XPS spectra for N 1*s* in Zn/NC-X and NC-950. Blue for pyridinic-N, magenta for Zn–N_x_, red for pyrrolic-N, olive for graphitic-N, and navy for oxidized-N.

The key factors for the excellent performance of Zn/NC-950 were then investigated. Initially, as examined by XPS (Fig. 2), all Zn/NC-X samples contained the Zn–N_x_ species, and the relative content of Zn–N_x_ was decreased in the order Zn/NC-950 (50.8 %) > Zn/NC-1000 (12.1%) > Zn/NC-900 (8.8%) > Zn/NC-800 (7.5%) > NC-950 (0%), which is proportional to their catalytic activity (Supplementary Fig. 7, Table 1, Entries 2–5 and 13), indicating that the Zn^2+^ ions coordinated with N species were indeed responsible for the high efficiency of Zn/NC-950. In addition, we investigated the proton/deuterium kinetic isotope effect of the reaction (Supplementary Fig. 8). The deuterium-labeling studies reveal a primary kinetic isotope effect (KIE) (*k*_H_/*k*_D_ = 2.18) in the oxidative cleavage of acetophenone, which indicates that the *α*-C_*sp*3_–H bond oxidation is the rate-determining step. According to the literature, the *α*-C_*sp*3_–H of ketones could be effectively acidified by the cooperation of acid-base sites^42–45^. The coordinated Zn^2+^ ions and the N species could form an enhanced Lewis acid-base pair, where single Zn sites in the Zn^δ+^ (0<*δ*<2) oxidation state, possessing empty orbitals to accept electrons, serve as Lewis acid sites, and their surrounding N atoms serve as Lewis base sites^32^. Thus, we deduce that the ZnN_x_ structure played an important role in the oxidation of *α*-C_*sp*3_–H, and propose a plausible acetophenone activation model (Supplementary Fig. 9), in which the Zn polarizes the carbonyl group of ketones, acidifies the *α*-C_*sp*3_–H, promoting its abstraction. The activation of acetophenone by ZnN_4_ structure was further confirmed by DFT calculation. With the activation of the ZnN_4_ center, the C=O bond increased from 1.235 to 1.249 Å, and the C(CO)–C bond decreased from 1.52 to 1.503 Å, indicating that the carbonyl group in acetophenone was polarized by the ZnN_4_ site, which makes the carbon more electrophilic and the *α*-H more acidic^44^, thereby facilitating the abstraction of the *α*-H by the superoxide radical to form hydroperoxide, as shown in Supplementary Fig. 10, which will be discussed below.

**Fig. 3:**
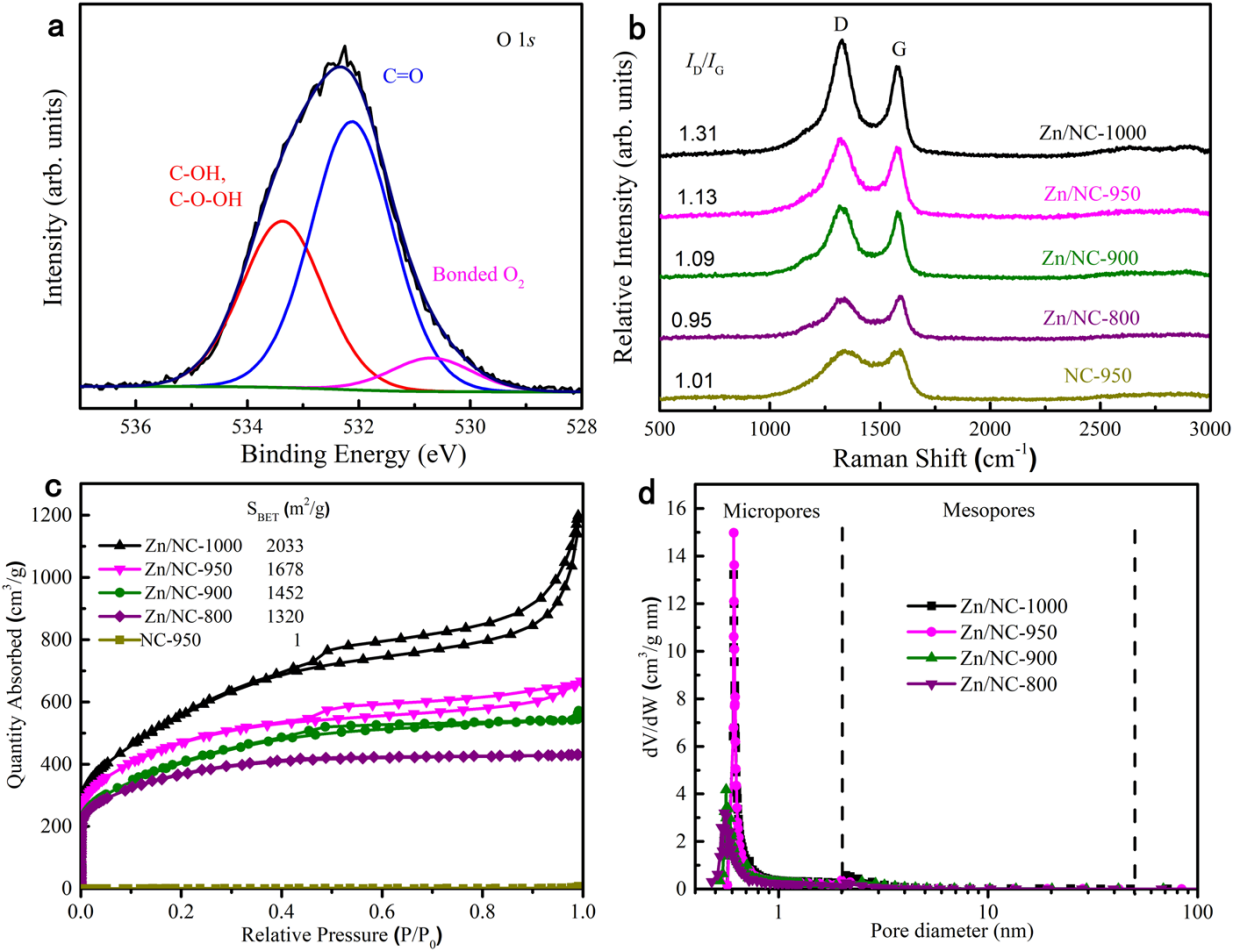
Characterization of the synthesized Zn/NC-X. **a** O 1*s* XPS spectrum of Zn/NC-950. **b** Raman spectra. **c** N_2_ adsorption–desorption isotherms. **d** Pore size distribution curves (calculated by the Saito–Foley method (<2 nm) and the Barrett–Joyner–Halenda method (>2 nm)) of Zn/NC-X and NC-950.

Furthermore, it has been widely accepted that the ZnN_x_ sites^46^, *sp*2 carbon^47^, defects^48^, graphitic-N^48,49^, and carbon atoms adjacent to the graphitic-N^50^ all showed good performance for the oxygen activation. According to Song’s work^46^, the Zn–N_4_ site represented an easier or faster ORR process than any other pure N–C site and pristine C, indicating an easier or faster O_2_ activation than that with N–C active sites and pristine C. We also computed the O_2_ activation on the carbon-supported ZnN_4_ site by DFT calculation. The differential charge density diagram shows that an electron is transferred from Zn–N_4_ to O_2_ and concentrated at both ends of O–O (Supplementary Fig. 11), and the O–O bond length of O_2_ changed from 1.234 to 1.297 Å, agreeing with the reported 1.32 Å and calculated 1.303 Å bond distance of ${{{{{{\rm{O}}}}}}}_{2}^{-}$• species^51^. These calculation results suggest that the carbon-supported ZnN_4_ enhanced the kinetics for the activation of molecular oxygen by a sequence of electron transport and reduction to superoxide radical, which has a strong oxidizing ability. The O 1*s* XPS of Zn/NC-950 was also performed to confirm the O_2_ adsorption. The O 1*s* spectrum of Zn/NC-950 was fitted into three peaks centered at 533.3, 532.1, and 530.6 eV (Fig. 3a). Peaks located at 530.6 eV could be attributed to the physically absorbed oxygen^52^. The strong gas adsorption enables them to enrich and activate the gas, especially polarizable O_2_ for chemical transformation^52,53^.

In addition, the Raman spectra showed that all samples displayed one intense D band at ~1324 cm^−1^ and G band at ~1582 cm^−1^. The 2D band of graphite was ignorable and the broad and strong D peak suggests the existence of edge structures in the Zn/NC-X samples (Fig. 3b). The intensity ratio of *I*_D_/*I*_G_ increased with the increase of the pyrolysis temperature, indicating that more defects were generated with the increasing temperature. The N_2_ adsorption–desorption isotherms (Fig. 3c) show that the prepared Zn/NC-X samples have much higher BET surface area, while the BET surface area for NC-950 was only 1 m^2^/g. The pore size distribution curves confirmed the existence of micropores in all Zn/NC-X samples (Fig. 3d). The structural defects and microporous structure with high surface area may be caused by the high pyrolysis temperature and the evaporation of zinc^54^.

It can be known from the results above that the Zn single sites coordinated with their surrounding N on microporous NC material serves as the active sites to acidify the *α*-C_*sp*3_–H bond in acetophenone, and activate the molecular oxygen. Besides, high specific surface areas, suitable porous structure, and surface enriched defects enabled sufficient exposure and improved accessibility of active sites, which kinetically favors the substrate activation (ketones, alcohol, and O_2_ activation)^52,53^, acceleration of the mass transfer, leading to the excellent catalytic activity of the synthesized Zn/NC-950.

### Studies of reaction mechanism

**Fig. 4:**
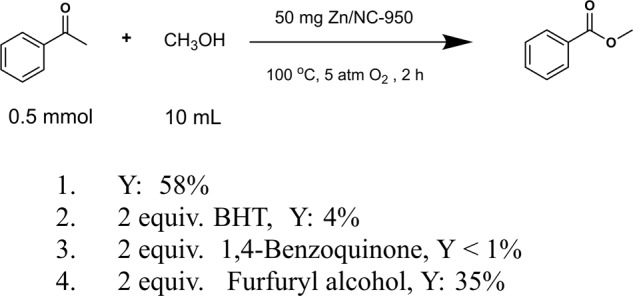
The radical inhibition experiments. Reaction conditions: substrate (0.5 mmol), ethylbenzene (0.5 mmol), Zn/NC-950 (50 mg), methanol (10 mL), O_2_ (5 atm), 100 °C, 2 h, the additive was added as above.

To better elucidate the mechanism of the oxidative cleavage of ketones to esters, we first conducted the radical inhibition experiments (Fig. 4). When a radical scavenger 2,6-di-tert-butyl-4-methylphenol (BHT) was added, the yield of methyl benzoate sharply decreased from 58 to 4%, indicating a possible radical reaction process. When 1,4-Benzoquinone (a quenching agent to scavenge super oxygen radical) was added, the reaction completely quenched. We further added furfuryl alcohol (a quenching agent to scavenge singlet oxygen) to the reaction system, the yield of methyl benzoate decreased slightly. These results suggest that the oxidative cleavage of ketones was proceeded by ${{{{{{\rm{O}}}}}}}_{2}^{-}$• radicals process. The electron paramagnetic resonance (EPR) measurements were also performed to verify the reactive oxygen species involved in the reaction with 5,5-dimethyl-1-pyrroline *N*-oxide (DMPO) as the trapping reagent (Supplementary Fig. 12). Four obvious signals with Zn/NC-950 in methanol were obtained, which confirmed the existence of DMPO-${{{{{{\rm{O}}}}}}}_{2}^{-}$•^55,56^.

**Fig. 5:**
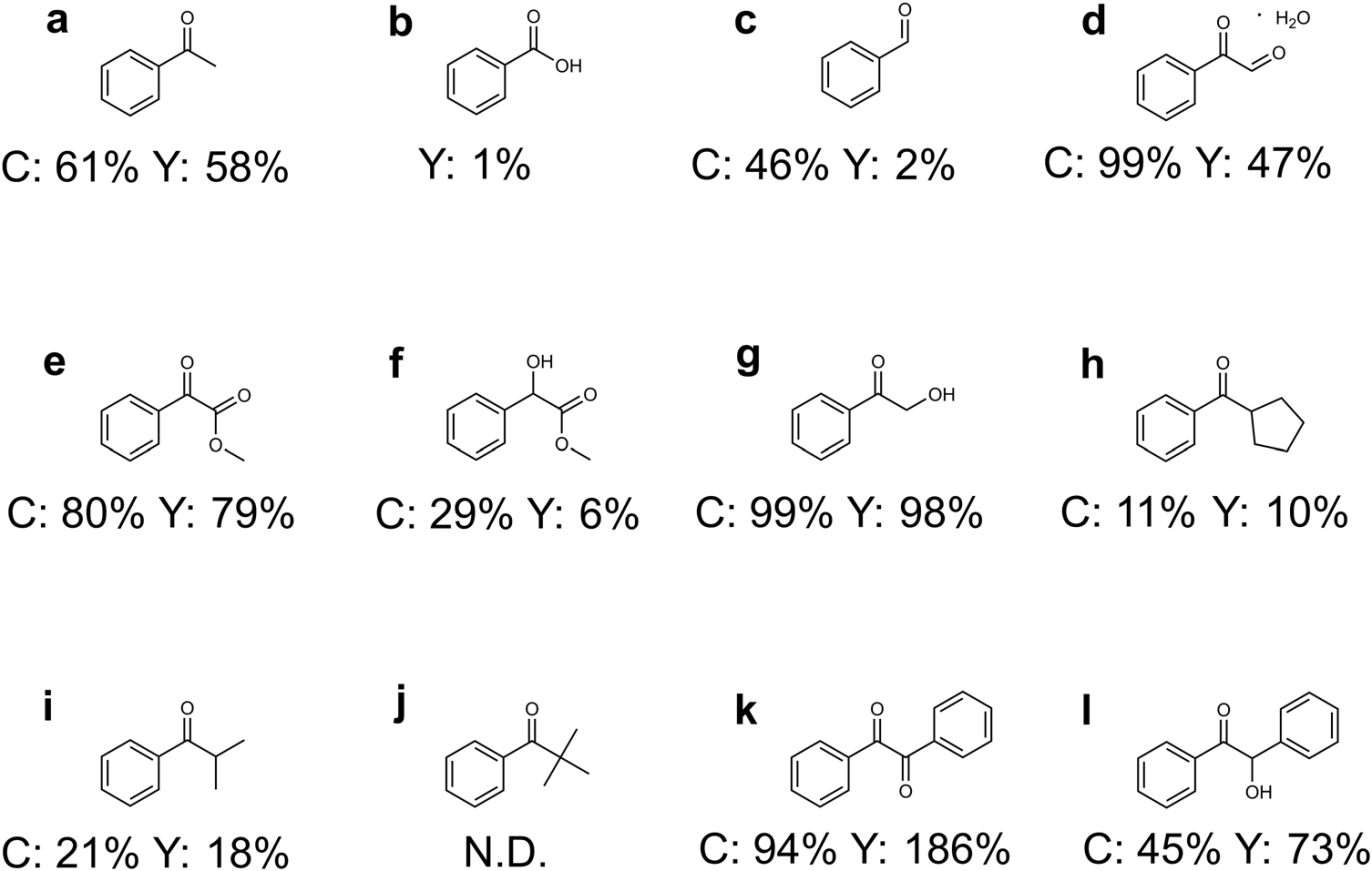
Control experiments. **a**–**l** The substrates used in the control experiments. Reaction conditions: substrate (0.5 mmol), ethylbenzene (0.5 mmol), Zn/NC-950 (50 mg), methanol (10 mL), O_2_ (5 atm), 100 °C, 2 h (120 °C, 12 h for **h**–**j**).

Several potential intermediates were then investigated. The benzoic acid (Fig. 5b) and benzaldehyde (Fig. 5c) afforded a low yield of methyl benzoate (1% and 2%, respectively), and the benzaldehyde was mainly converted into benzaldehyde dimethyl acetal via acetalization reaction, indicating that the possibility of benzaldehyde or benzoic acid as intermediate could be ruled out. When 2-oxo-2-phenylacetaldehyde (Fig. 5d) was employed as the substrate, the reaction affords methyl benzoate in 47% yield, with methyl benzoylformate and methyl mandelate as the side products. The lower yield than that obtained from conversion of acetophenone suggests that the main reaction route through 2-oxo-2-phenylacetaldehyde as intermediate can be excluded, but it might be an alternative route because when ethanol was used as solvent, the corresponding ethyl benzoylformate and ethyl mandelate could be easily detected by GC-MS (Supplementary Fig. 13). Besides, methyl benzoylformate (Fig. 5e) and methyl mandelate (Fig. 5f) can also be converted into methyl benzoate under reaction conditions, and the methyl mandelate was converted into methyl benzoate first. When 2-hydroxyacetophenone (Fig. 5g) was employed as the substrate, 98% yield of methyl benzoate was obtained, which was much higher than that of 2-oxo-2-phenylacetaldehyde. Furthermore, the cyclopentyl(phenyl)methanone (Fig. 5h) and isobutyrophenone (Fig. 5i) could also yield the methyl benzoate successfully, and cyclopentanone was detected as the split product for cyclopentyl(phenyl)methanone (Supplementary Fig. 14). These results confirm the possibility of oxidative cleavage of ketones through α-hydroxyketone directly, and the aldehydes or ketones would be the split product. However, no methyl benzoate was detected when using 2,2-dimethyl-1-phenyl-propan-1-one as the substrate (Fig. 5j), indicating that the oxygenation of the *α*-C_*sp*3_–H would be the key step and at least one *α*-C_*sp*3_–H is required. Notably, we also tested benzil (Fig. 5k, with *α*-diketone structure) as the substrate, the result clearly showed that the Zn/NC-950 catalytic systems could effectively oxidize the *α*-diketone bonds into esters. When benzoin (Fig. 5l, with *α*-hydroxyketone structure) was tested, the products distribution obviously presented two possible reaction pathways. On the one hand, most of the benzoin was oxidized into benzil and then converted into 2 equiv. of methyl benzoate via *α*-diketone cleavage. On the other hand, a small part of benzoin was converted into methyl benzoate via *α*-hydroxyketone directly, with benzaldehyde as the split product (Supplementary Fig. 15, most of the benzaldehyde was then converted into benzaldehyde dimethyl acetal).

**Fig. 6:**
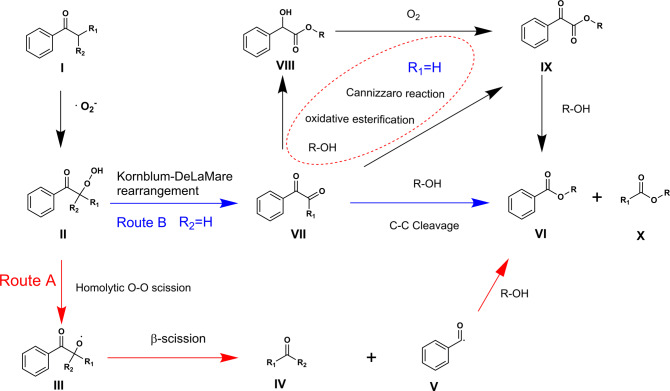
The proposed reaction mechanism of the oxidative cleavage of C(CO)–C bonds of ketones to esters over Zn/NC-950 catalyst. Two pathways were proposed for this transformation: cleave via α-oxy radical (Route A) and cleave via α-diketone (Route B).

On the basis of the observed experimental results and previous reports^10,25,57,58^, a possible mechanism for the aerobic oxidative cleavage of C(CO)–C bond to esters over Zn/NC-950 was proposed (Fig. 6). Initially, the ketone I was oxidized to hydroperoxide II through the super oxygen radical^10,57^. Subsequently, two reaction pathways proceeded to form the desired ester. In one pathway (Route A), II might be homolytically cleaved to the corresponding *α*-oxy radical III, and then cleaved to ketones/aldehydes IV and benzaldehyde radical species V via *β*-scission^56^. The V was then reacted with R-OH, giving ester as the final products and the ketones/aldehydes as the split products. In another pathway (Route B), when R_2_ = H, the intermediates II could be converted into VII via Kornblum–DelaMare rearrangement^59^, and then directly cleaved to the corresponding esters via *α*-diketone cleavage. When R_1_ = H, the intermediate VII could be further converted into VIII and IX by intramolecular cannizzaro and oxidative esterification reaction^60,61^, then converted into VI as the final product.

**Fig. 7:**
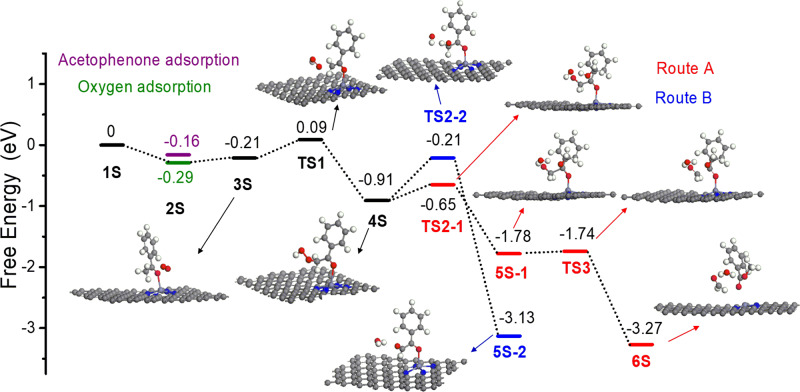
Energy profiles. The oxidative cleavage of acetophenone with methanol to methyl benzoate was chosen to simplify the calculation.

To shed more light on the origin of the high catalytic activity of Zn/NC-950, DFT calculations were performed to explore the possible reaction mechanism. The oxidative cleavage of acetophenone with methanol to methyl benzoate was chosen to simplify the calculation (Fig. 7). We first investigated the competitive adsorption between acetophenone and O_2_ molecule on the carbon-supported ZnN_4_ site. The O_2_ molecule has slightly higher adsorption energy than that of acetophenone (Fig. 7, 2S, −0.29 eV vs −0.16 eV). Thus, we considered O_2_ molecule adsorption as the first step in the reaction path. After the adsorption of O_2_, the acetophenone could also be adsorbed, only 0.08 eV energy was needed for the adsorption of acetophenone while keeping superoxide-like O_2_ species close to acetophenone and ZnN_4_ site. Along the reaction path, the superoxide-like O_2_ species interacted with the acidified *α*-C_*sp*3_–H in acetophenone and triggered the oxidation reaction with a moderated activation energy of 0.3 eV. The red line shows the homolytic cleavage of 4S to the corresponding *α*-oxy radical and hydroxyl radical (Fig. 7, TS2-1, illustrated as Route A in Fig. 6). The generated hydroxyl radical would rapidly react with methanol to water and methoxy radical, the *α*-oxy radical could also transfer to the benzaldehyde radical and formaldehyde via *β*-scission. Then the methoxy radical reacted with the benzaldehyde radical, giving methyl benzoate as the final product. For Route B, much higher activation energy was needed for the Kornblum–DelaMare rearrangement of 4S to 5S-2 (0.7 eV vs 0.26 eV), indicating that route A was the main reaction route for the oxidative cleavage of acetophenone with methanol to methyl benzoate, which agrees well with the experimental results. On the basis of the above calculation results, the C_*α*_–H bond oxidation would be the rate-determining step for the transformation of acetophenone to methyl benzoate, which was in line with the observed primary KIE effect. The C_*α*_–H bond oxidation involves the C_*α*_–H bond acidification and O_2_ activation, further confirming the essential role of ZnN_4_ sites in Zn/NC-950.

## Discussion

In summary, we fabricated Zn single sites coordinated with N species on microporous NC for aerobic oxidative cleavage of C(CO)–C(alkyl) bond in ketones to esters. A series of acetophenone derivatives as well as more challenging alkyl ketones could be efficiently cleaved and esterified into the corresponding esters with high yield. The high activity of Zn/NC-950 could be attributed to the cooperation of Zn single sites with their surrounding N atoms, as well as the microporous structure with high surface area and structural defects. This work illustrates an example of fabricating very active catalyst from a traditionally unactive metal for a certain reaction, which leads to the possibility for catalyzing a reaction efficiently at mild condition. This opens a way for the design of efficient catalysts from cheap and earth-abundant metals.

## Methods

### Materials

Chitosan, acetophenone (99%), ethylbenzene (99%), 4-methoxyacetophenone (99%), 3′-methoxyacetophenone (98%), 2′-methoxyacetophenone (98%), 4′-methylacetophenone (98%), 4′-cyanoacetophenone (97%), 4′-fluoroacetophenone (98%), 4′-chloroacetophenone (97%), 4′-bromoacetophenone (98%), 4′-iodoacetophenone (98%), 4′-(methylsulfonyl)acetophenone (98%), 4′-nitroacetophenone (97%), 4′-(trifluoromethyl)acetophenone (98%), 4-acetyl-biphenyl (98%), 2′-acetonaphthone (98%), 3′,4′-(methylenedioxy)acetophenone (98%), 2-benzofuranyl methyl ketone (99%), 4-acetylpyridine (98%), 2-acetylthiophene (99%), 2-furyl methyl ketone (99%), 2-hydroxyacetophenone (98%), methyl benzoylformate (97%), benzaldehyde (99%), benzoic acid (99.5%), methyl DL-mandelate (97%), phenylglyoxal monohydrate (97%), phenylglyoxylic acid (95%), (R)-(-)-mandelic acid (99%), butyrophenone (99%), heptanophenone (98%), 4-phenyl-2-butanone (98%), cyclopentyl phenyl ketone (98%), isobutyrophenone (98%), benzylideneacetone (98%), 1-indanone (99%), 2-phenoxyacetophenone (98%), β-ionone (97%), α-ionone (90%), benzoin (98%), benzil (99%), 2,6-di-tert-butyl-4-methylphenol (BHT, 99%), *p*-benzoquinone (99%), furfuryl alcohol (99%), 2,5-dihydroxyterephthalic acid (98%), copper nitrate trihydrate (99%), cobalt nitrate hexahydrate (99%) and zinc phthalocyanine (95%) were purchased from Aladdin reagent Co., Ltd. 2,2-dimethylpropiophenone, acetophenone-d3 (98 atom% D) and 3′,4′-(Methylenedioxy)acetophenone (98%) were provided by J&K Scientific Ltd. 4-(2-furyl)-3-buten-2-one (98%) was purchased from TCI. Zinc chloride (99%), potassium thiocyanate (99%), methanol (99%), ethanol (99%), 1-propanol (99%), 1-butanol (99%), isopropanol (99%), hydrochloric acid (36.0–38.0%), and acetic acid (99.5%) were acquired from Sinopharm Chemical Reagent. All the chemical reagents were used as received without further purification.

### The preparation of Zn/NC-X and NC-950

The Zn/NC-X catalyst was prepared by the pyrolysis of Zinc chloride and the chitosan complex. Typically, 3 g chitosan and 6 g Zinc chloride were dissolved in 100 ml 5 wt% acetic acid aqueous solution and stirred into homogeneous semi-transparent paste. The solution was poured into Petri-dish and dried in an oven at 80 °C overnight. The obtained sample was then pyrolyzed at target temperature (800–1000 °C) for 2 h under N_2_ atmosphere with a ramp rate of 3 °C min^−1^. The obtained black powder was directly used without further treatment. The NC-950 catalyst was prepared by the pyrolysis of chitosan at 950 °C for 2 h under N_2_ atmosphere with a ramp rate of 3 °C min^−1^ directly.

### The preparation of Zn/NC-950-H

Typically, 100 mg Zn/NC-950 was immersed in 10 mL aqueous solution of HCl (1 M) under continued stirring for 5 h to remove the accessible Zn nanoparticles or clusters, and the solid was washed five times with de-ionized water to remove all possible residual acid. The Zn content was measured to be 0.62 wt% based on ICP analysis.

### The preparation of Zn/AC-900

The Zn/AC-900 was obtained by the pyrolysis of Zn-MOF-74. Typically, 13 mmol 2,5-dihydroxyterephthalic acid and 38 mmol zinc nitrate were dissolved in 500 mL DMF with stirring, followed by the addition of 25 mL de-ionized water. The mixture was heated in an oven at 100 °C for 20 h. The formed products were collected by centrifugation and washed with methanol thoroughly to remove the residual DMF. The synthesized sample was further dried at 60 °C under vacuum. The obtained sample was then pyrolyzed at 900 °C for 2 h under N_2_ atmosphere with a ramp rate of 3 °C min^−1^. The obtained black powder was directly used without further treatment.

### The preparation of Co/NC-950 and Cu/NC-950

The syntheses of Co/NC-950 and Cu/NC-950 are of similar procedures, and here we took Co/NC-950 as an example. Typically, 3 g chitosan and 1 mmol cobalt nitrate were dissolved in 100 ml 5 wt% acetic acid aqueous solution and stirred into homogeneous semi-transparent paste. The solution was poured into Petri-dish and dried in an oven at 80 °C overnight. The obtained sample was then pyrolyzed at 950 °C for 2 h under N_2_ atmosphere with a ramp rate of 3 °C min^−1^. The obtained black powder was directly used without further treatment. Cu/NC-950 was also prepared according to this route by changing the metal salt from Co(NO_3_)_2_ to Cu(NO_3_)_2_.

### Typical procedures for the aerobic oxidative cleavage and esterification of ketones

In a typical experiment, the desired substrates, catalyst, and solvent were charged into an autoclave (50 mL inner volume). After the reactor was sealed, it was purged with 5 atm. O_2_. Then, the reactor was put into a constant temperature air bath at the desired temperature, and the magnetic stirrer was started. After a certain reaction time, the reactor was placed into ice water, and then the gases in the reactor were released slowly and 0.5 mmol ethylbenzene as internal standard was added into the reactor followed by filtration. The reaction mixture was then analyzed quantitatively by GC (Agilent 7820A) equipped with a flame-ionized detector, and the products were confirmed by GC-MS (Agilent 7890A GC/5973 MS). To obtain the pure products, the reaction mixture was concentrated and purified by column chromatography (silica gel with n-hexane/EtOAc). The pure products were characterized by ^1^H NMR and ^13^C NMR. To examine the reusability of the Zn/NC-950, the catalyst was recovered by centrifugation, washed with methanol, and calcined at 400 °C under N_2_ for 2 h. Then the catalyst was used for the next run.

### Characterization

The scanning electron microscopy (SEM) measurements were performed on a Hitachi S-4800 scanning electron microscope operated at 15 kV. The Scanning transmission electron microscopy (STEM) images were obtained using a FEI Talos F200X instrument. Atomic-resolution HAADF-STEM images and EDS mappings were obtained from a fifth-order aberration-corrected transmission electron microscope (JEOL ARM200CF). The X-ray absorption spectra were collected on the beamline BL07A1 in NSRRC, and were provided technical support by Ceshigo Research Service “www.ceshigo.com”. The radiation was monochromatized by a Si (111) double-crystal monochromator. XANES and EXAFS data reduction and analysis were processed by Athena software. Powder X-ray diffraction (XRD) patterns were collected on a Bruker advanced D8 powder diffractometer using Cu Kα radiation. The N_2_ adsorption–desorption isotherm and pore size distribution of the samples were obtained from a nitrogen adsorption apparatus (V-Sorb 2800P). X-ray photoelectron spectroscopy (XPS) measurements were carried out on a ESCAL Lab 220i-XL spectrometer. Raman spectra were measured on a confocal laser micro-Raman spectrometer (Thermo Fischer DXR) at room temperature. The content of Zn in the Zn/NC-X was determined by inductively coupled plasma atomic emission spectroscopy (ICP-AES VISTA-MPX). ^1^H and ^13^C nuclear magnetic resonance (NMR) spectra were obtained on Bruker Ascend III TM600 MHz NMR spectrometer. EPR spectroscopy was carried out on a JES-FA 200 ESR Spectrometer at the X-band at room temperature with a field modulation of 100 kHz. After reaction for 2 h, the spin-trapping reagent DMPO was added to the reaction mixture, and measured in a glass capillary tube by EPR spectroscopy at room temperature.

### Theoretical method

The DFT calculations were carried out by using VASP with the gradient-corrected PBE exchange-correction functional. The model containing 78 C atoms, 4 N atoms, and 1 Zn atom, where a vacuum of 18 Å was used to simulate the surface in periodic boundary condition. The energy cutoff for the plane-waves was set to 400 eV. The Brillouin zone integration was performed using a Monkhorst-Pack grid of 2 × 2 × 1 special k-points. All the atomic positions were relaxed until all the remaining forces on these atoms are <0.02 eV/Å. The transition states (TS) were searched by the “climbing images” nudged elastic band (CI-NEB) algorithm.

## Supplementary information

Supplementary Information

## Data Availability

All data needed to evaluate the conclusions in the paper are present in the paper and/or the Supplementary Information. Additional data are available from authors upon request.
