# Supplementary material for: Zn-Nx sites on N-doped carbon for aerobic oxidative cleavage and esterification of C(CO)-C bonds
Source: Nat Commun. 2021 Aug 10;12:4823. doi: 10.1038/s41467-021-25118-0 (PMC8355145; doi:10.1038/s41467-021-25118-0)
Supplement: Supplementary file 1 — Supplementary Information [file 41467_2021_25118_MOESM1_ESM.pdf]

Supplementary Information for

Zn-N_x sites on N-doped Carbon for Aerobic Oxidative Cleavage and Esterification of C(CO)-C Bonds

Chao Xie,[1] Longfei Lin,[2] Liang Huang,[3] Zixin Wang,[1] Zhiwei Jiang,[1] Zehui Zhang, [1]* and Buxing Han[2]*

[1]Key Laboratory of Catalysis and Energy Materials Chemistry of Ministry of Education & Hubei Key Laboratory of Catalysis and Materials Science, South-Central University for Nationalities, Wuhan 430074, China;

[2]Beijing National Laboratory for Molecular Sciences, CAS Key Laboratory of Colloid, Interface and Chemical Thermo-dynamics, Institute of chemistry, Chinese Academy of Sciences, Beijing 100190, China;

[3]The State Key Laboratory of Refractories and Metallurgy, Wuhan University of Science and Technology, Wuhan, 430081, China.

E-mail: zehuizh@mail.ustc.edu.cn； hanbx@iccas.ac.cn

Table of Contents

Supplementary Figures 2

Supplementary Tables 10

Supplementary data 13

Supplementary References 51

Supplementary Figures

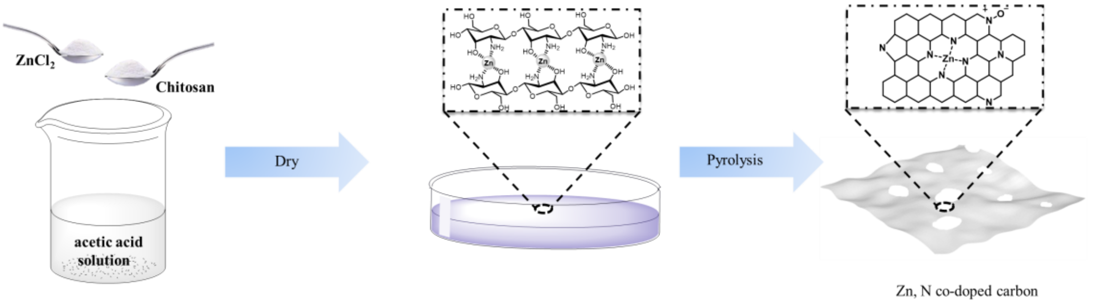

Supplementary Figure 1 Schematic illustration of the fabrication process of the Zn/NC-X.

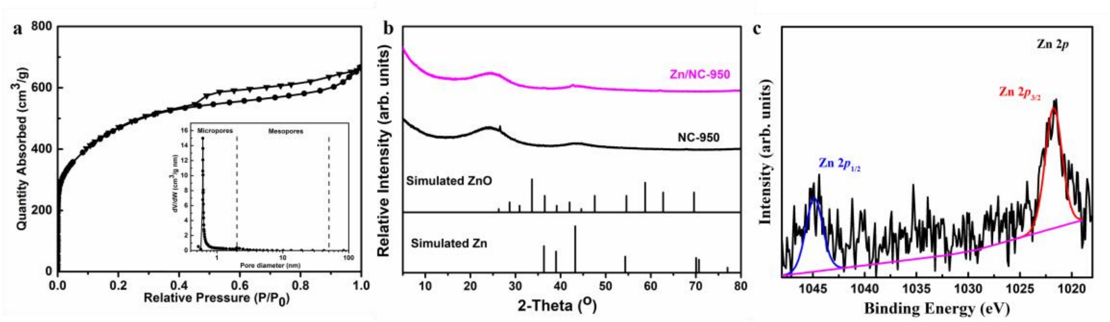

Supplementary Figure 2 Characterization of the synthesized Zn/NC-950 catalyst. **a** N_2 adsorption-desorption isotherms (Insert: pore size distribution curves calculated by the Saito-Foley method (<2 nm) and the Barrett-Joyner-Halenda method (>2 nm) of Zn/NC-950. **b** XRD patterns of Zn/NC-950 and NC-950. **c** Zn 2*p* XPS spectrum for Zn/NC-950.

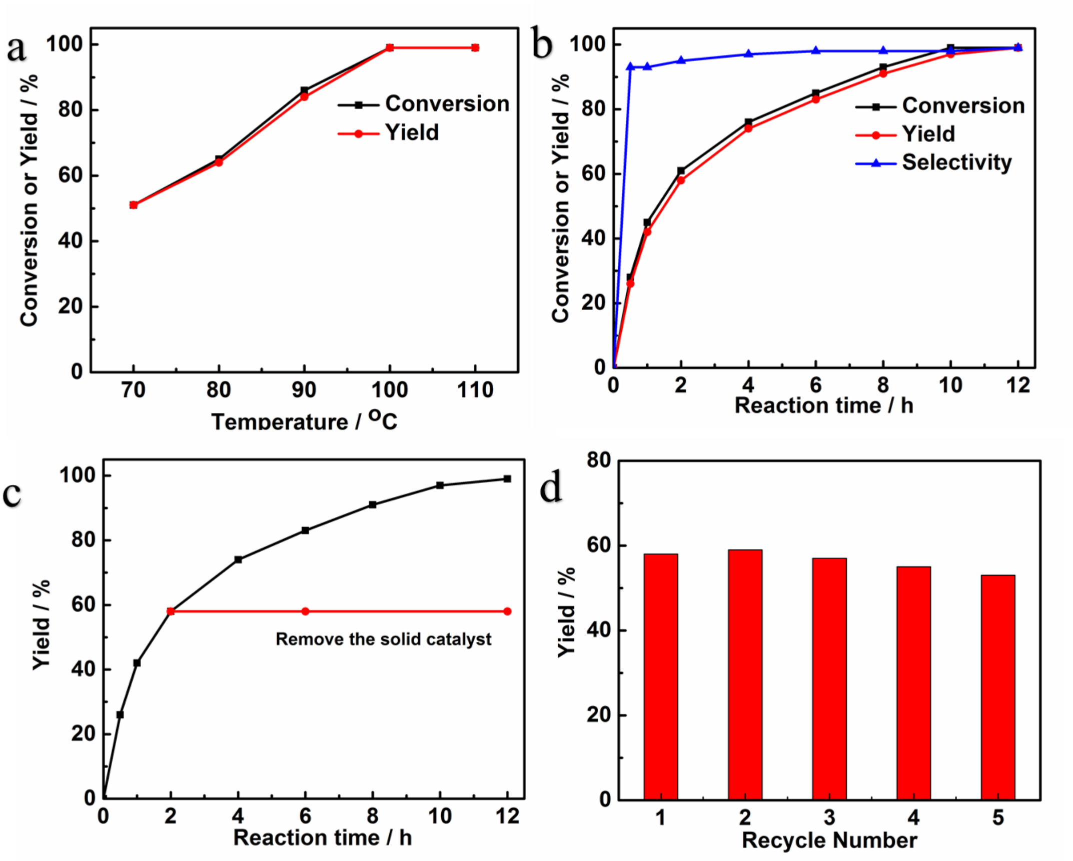

Supplementary Figure 3 The optimization of reaction conditions and recycling test of the Zn/NC-950 catalyst. a Effect of Reaction temperature. **b** Effect of reaction time. **c** Time-yield plots over Zn/NC-950 (black line) or removing Zn/NC-950 after 2 h (red line). **d** Reusability of Zn/NC-950. Reaction conditions: acetophenone (0.5 mmol), ethylbenzene (internal standard, 0.5 mmol), Catalyst (50 mg), methanol (10 mL), O_2 (5 atm), 12 h for a, 100 °C for b and c, d and 2h for d.

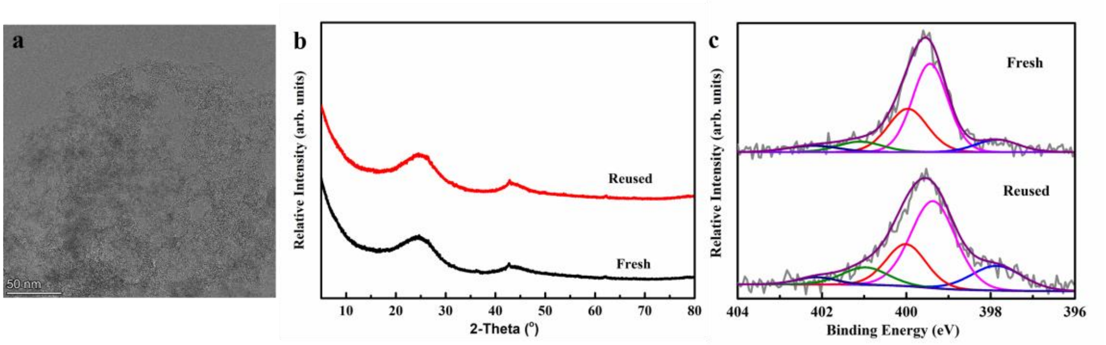

Supplementary Figure 4 Characterization of the reused Zn/NC-950 catalyst. a TEM, **b** XRD and **c** N 1*s* XPS.

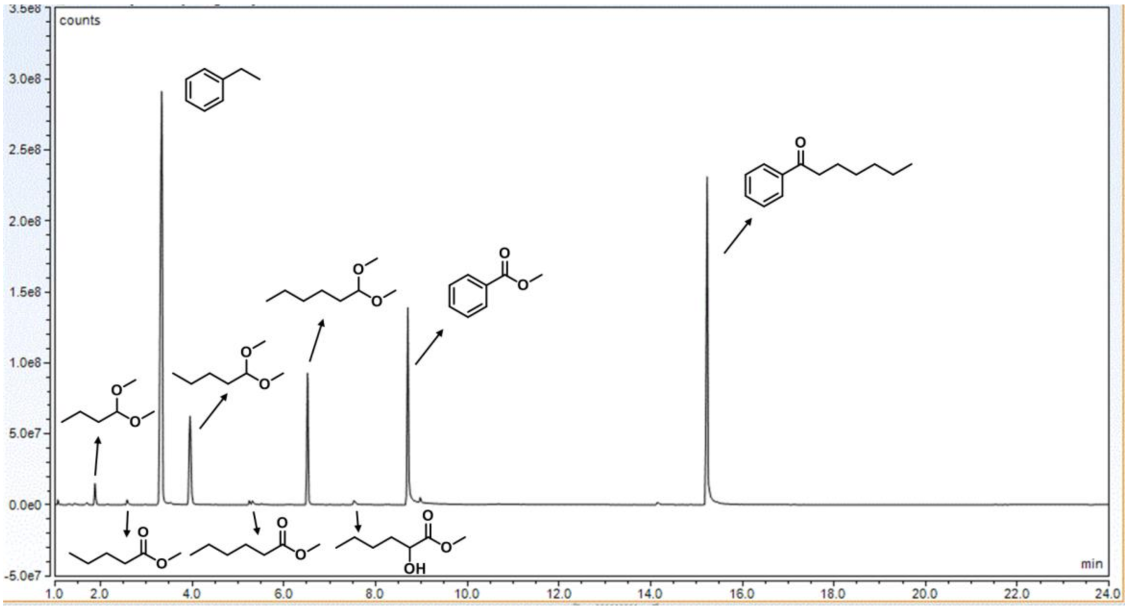

Supplementary Figure 5 GC-MS spectrum of the intermediate generated from the aerobic oxidation cleavage and functionalization of heptanophenone in methanol.

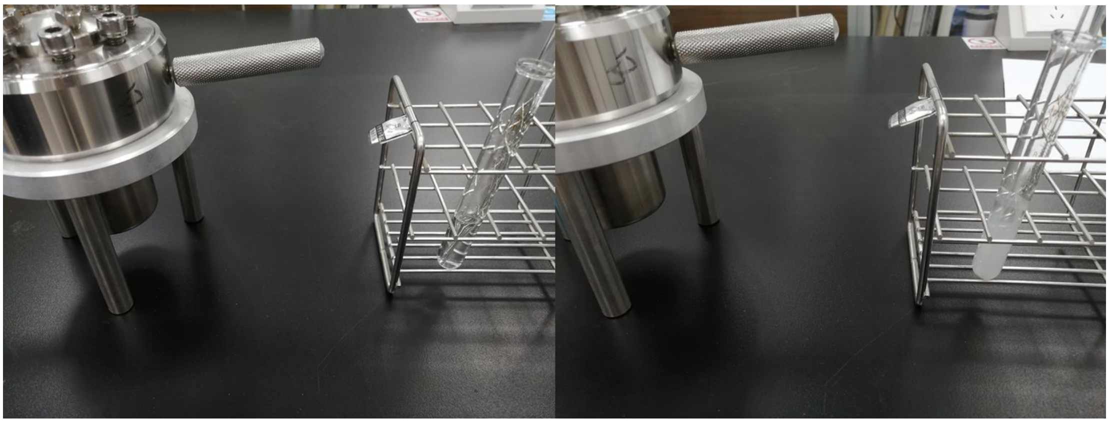

Supplementary Figure 6 Before (left) and after (right) the lime-water test for the aerobic oxidation cleavage and functionalization of heptanophenone in methanol.

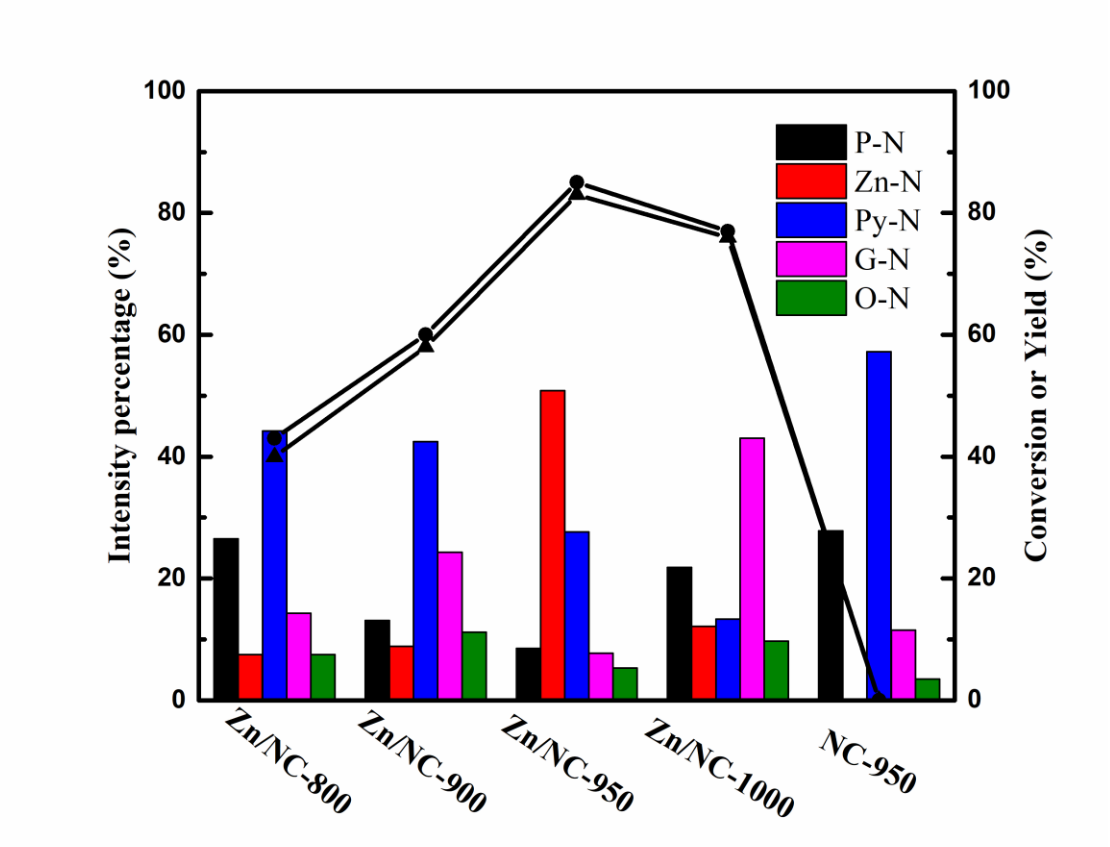

Supplementary Figure 7 N 1*s* regions composition by XPS analysis for Zn/NC-X, and their corresponding reaction efficiency in oxidation cleavage and esterification of acetophenone. P-N for Pyrodinic-N, Py-N for Pyrrolic-N, G-N for Graphitic-N and O-N for Oxidized-N.

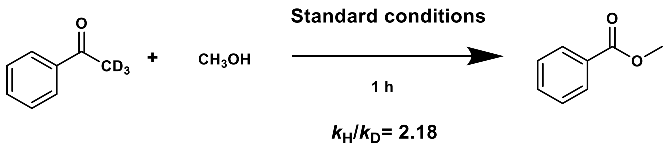

Supplementary Figure 8 Proton/deuterium kinetic isotope effect of the reaction. Reaction conditions: substrate (0.5 mmol), ethylbenzene (0.5 mmol), Zn/NC-950 (50 mg), methanol (10 mL), O_2 (5 atm), 100 °C, 1 h.

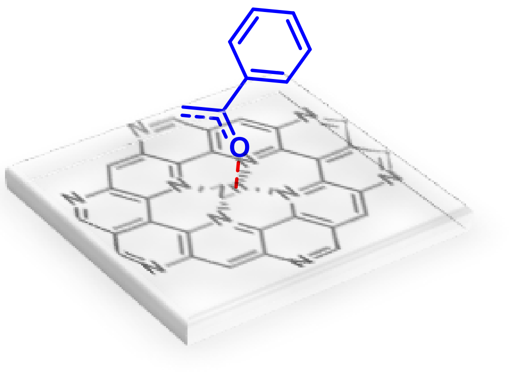

Supplementary Figure 9 The activation model of acetophenone on Zn/NC-950.

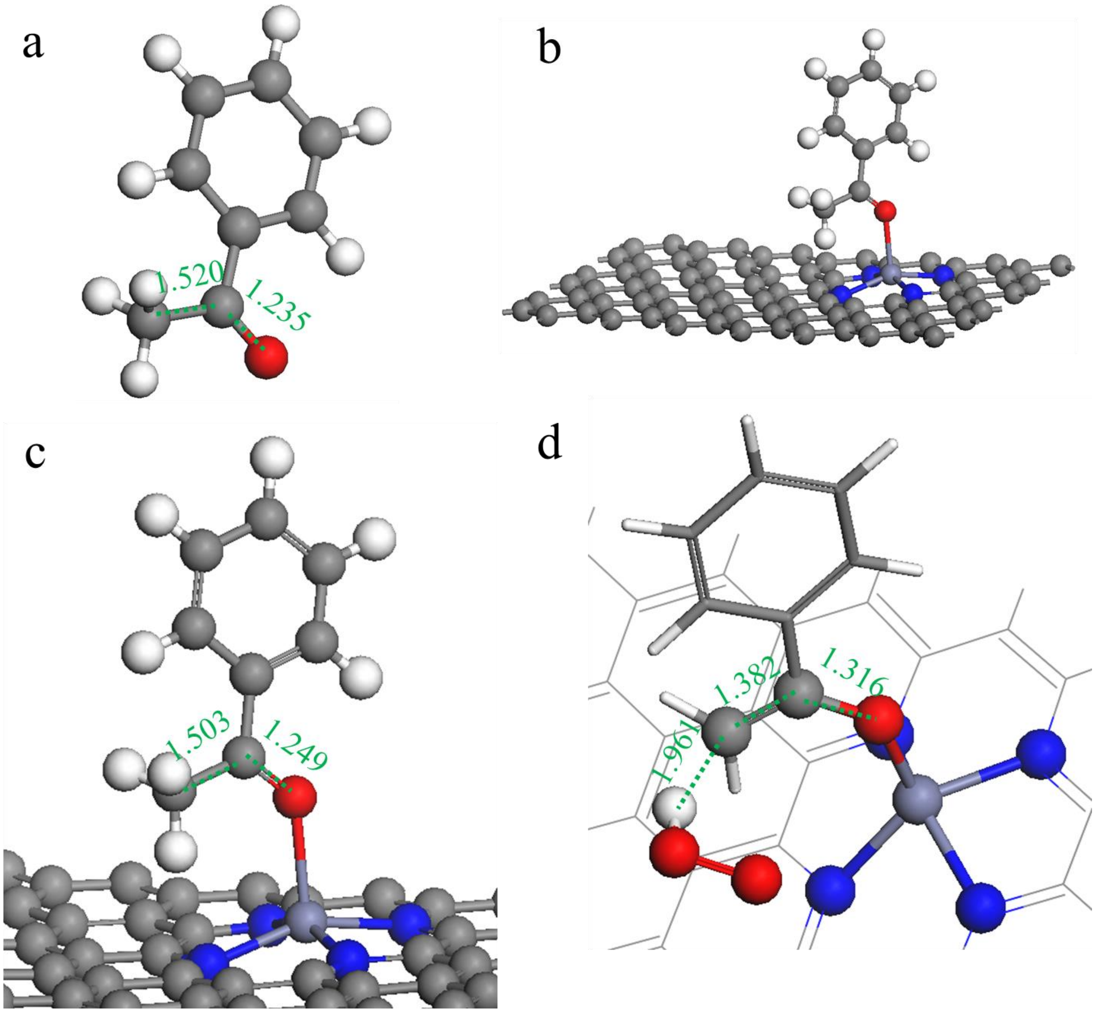

Supplementary Figure 10 The activation of acetophenone by ZnN_4 site. a The calculated bond length of acetophenone. **b** The adsorption of acetophenone on carbon supported ZnN_4 site. **c** The calculated bond length of acetophenone with the activation of carbon supported ZnN_4 site. **d** The abstraction of the α-H in acetophenone by the

superoxide radical.

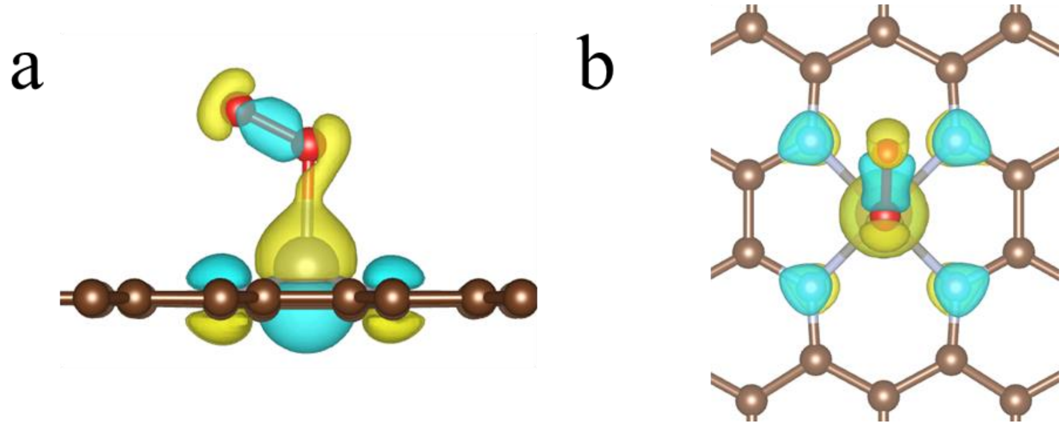

Supplementary Figure 11 The differential charge density diagram for the O_2 activation on carbon supported ZnN_4 site. a Front view. **b** Top view. The yellow and blue areas represent electron gains and losses, respectively.

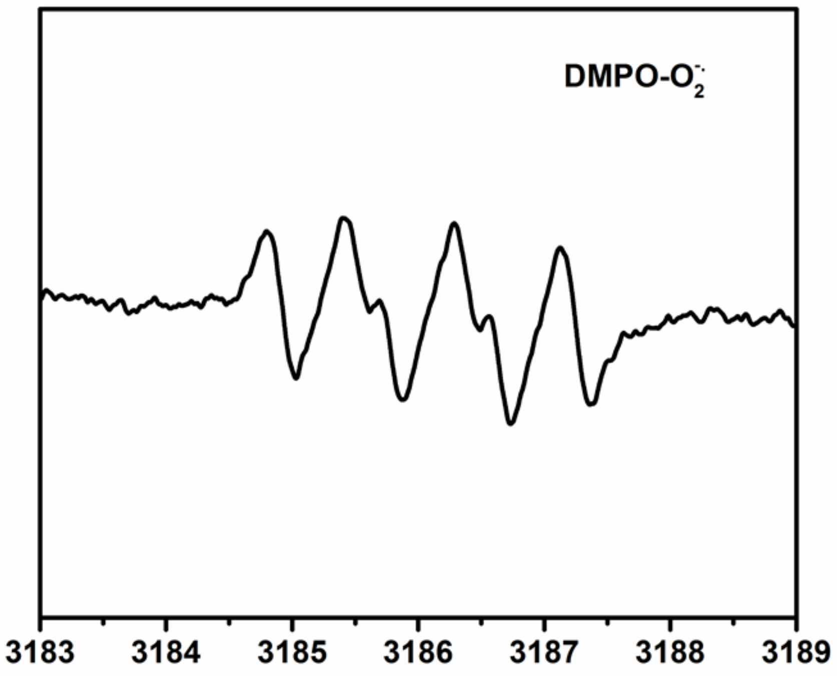

Supplementary Figure 12 DMPO spin-trapping EPR spectrum of the aerobic oxidation cleavage and functionalization of acetophenone with Zn/NC-950 in methanol.

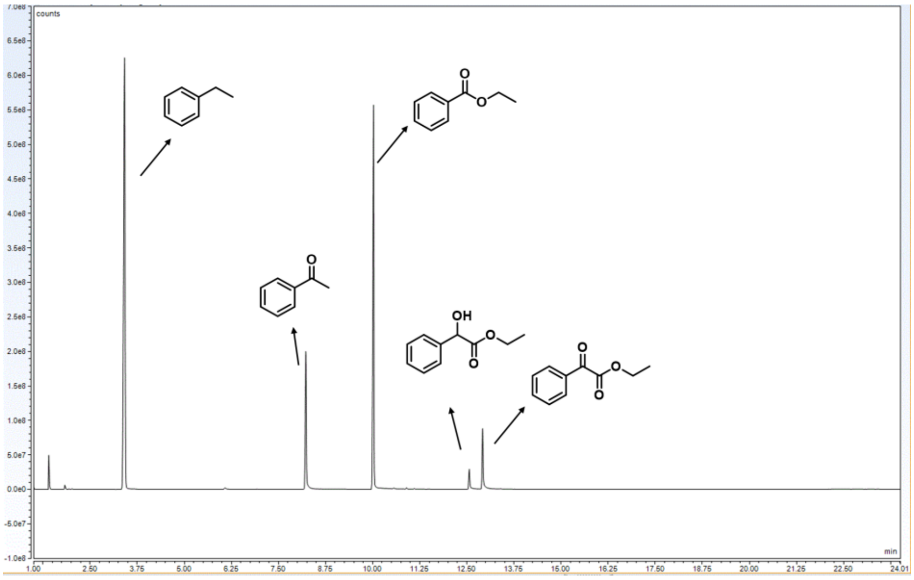

Supplementary Figure 13 GC-MS spectrum of the intermediate generated from the aerobic oxidation cleavage and functionalization of acetophenone in ethanol.

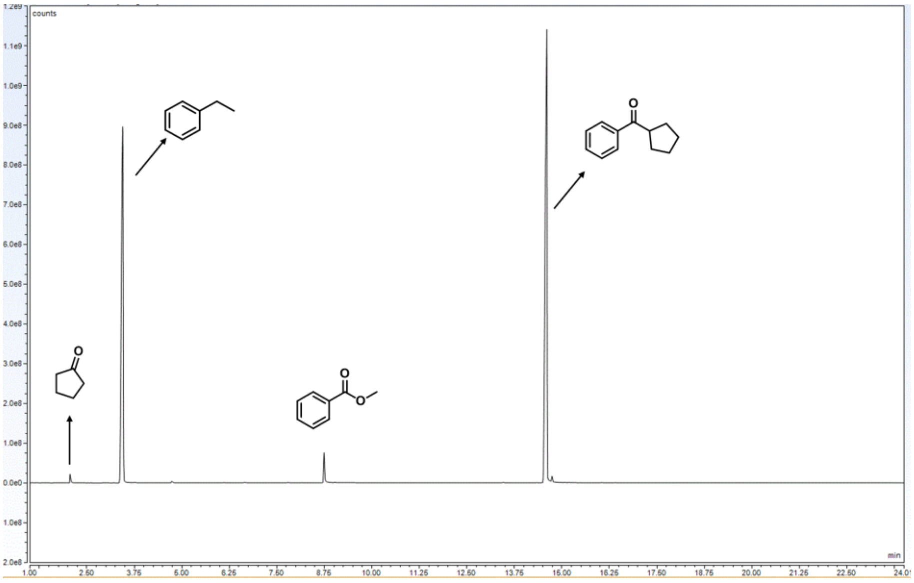

Supplementary Figure 14 GC-MS spectrum of the intermediate generated from the aerobic oxidation cleavage and functionalization of cyclopentyl(phenyl)methanone in methanol.

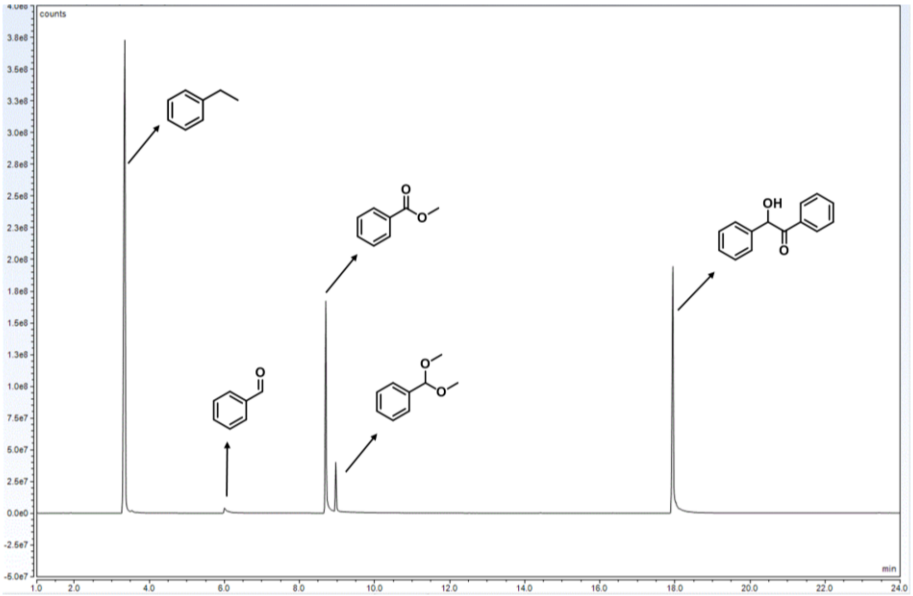

Supplementary Figure 15 GC-MS spectrum of the intermediate generated from the aerobic oxidation cleavage and functionalization of benzoin in methanol.

Supplementary Tables

Supplementary Table 1 Comparison of Zn/NC-950 with state-of-the-art catalysts in Cleavage and functionalization of acetophenone.

Ent	Cat (mol%)	Addit (mol%)	Oxid	Conditions	Prod	Y %	TOF/h^{-1}	Ref
1	Zn/NC-950 (1.1)	-	5 atm O_2	70 °C, 12 h	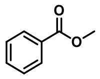	51	3.8	This work
2	Zn/NC-950 (1.1)	-	5 atm O_2	100 °C, 12 h	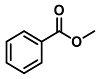	>99	7.5	This work
3	Co-NC-900 (7.5)	K_2CO_3 (10)	6 atm O_2	150 °C, 24 h	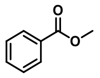	99	0.6	1
4	$CuCl_2$ (40)	1,10-phenanthroline (40), NIS (200), K_2CO_3 (500), 100 mg 4 Å MS	5 atm O_2	140 °C, 12 h	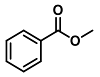	98	0.2	2
5	$CuCl_2·2H_2O$ (20)	LiBr (15)	10 atm O_2	130 °C, 10 h	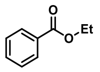	65	0.3	3
6	CuBr (10)	Pyridine (200) and $BF_3·Et_2O$ (100)	open air	130 °C, 10 h	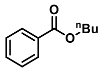	63	0.6	4
7	CuI (30)	-	O_2 (balloon)	120 °C, 15 h	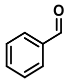	92	0.2	5
8	$CuCl_2$ (10)	TEMPO (20), H_2O (3000)	O_2 (1 atm)	120 °C, 16 h	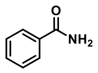	66	0.4	6
9	$Cu(OAc)_2$ (30)	TBAI (30)	O_2 (1 atm)	120 °C, 5 h	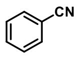	80	0.5	7
10	$Mn(OAc)_2$	-	O_2 (1 atm)	100 °C, 15 h	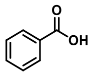	97	12.9	8

	(0.5)							
11	NH_4I (10)	-	3 equiv. oxone	60 °C, 15 h.	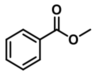	96	0.64	9
12	$AgNO_3$ (200)	-	1.2 equiv. I_2	R.T.,	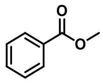	90	-	10
14	-	-	O_2	α,α,α-tribromoacetophenone, $h\nu<300$ nm, 2 h,	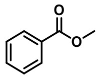	76	-	11

Ent for Entry, Cat for Catalyst, Addit for additives, Oxid for Oxidant, Prod for Product, R.T. for room temperature, Y for Yield and Ref for Reference.

Supplementary Table 2 EXAFS fitting parameters at the Zn K-edge for Zn/NC-950 （S_0^2=0.89）

Sample	Shell	CN	R(Å)	σ^2	ΔE_0	R factor
Zn foil	Zn-Zn	6	2.65±0.05	0.0215	3.6±1.6	0.0061
	Zn-Zn	6	2.65±0.05	0.0105		
ZnO	Zn-O	4	1.96±0.01	0.0046	2.6±0.7	0.0019
Zn/NC-950	Zn-N	4.4±0.2	2.03±0.01	0.0063	0.2±1.2	0.0110
	Zn-Zn	0.3±0.1	2.84±0.02	0.0031		

CN: coordination numbers; *R*: bond distance; σ^2: Debye-Waller factors; ΔE_0: the inner potential correction. *R* factor: goodness of fit. S_0^2 was set to 0.89, according to the experimental EXAFS fit of ZnO reference by fixing CN as the known crystallographic value; δ: percentage.

Supplementary Table 3 Concentration of surface nitrogen species in Zn/NC-950

before and after the repeated oxidation runs.

Catalyst	Total N	P-N	Zn-N	Py-N	G-N	O-N
Fresh	2.46	0.21	1.25	0.68	0.19	0.13
Reused	2.24	0.4	1.24	0.53	0.25	0.09

Supplementary data

Methyl benzoate (**1a**)

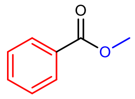

^{1}H NMR (300 MHz, $CDCl_3$) δ 8.08-7.99 (m, 2H), 7.55 (t, J = 7.3 Hz, 1H), 7.43 (t, J = 7.6 Hz, 2H), 3.91 (s, 3H). ^{13}C NMR (75 MHz, $CDCl_3$) δ 167.40 (s), 133.19 (s), 130.47 (s), 129.86 (s), 128.65 (s), 52.37 (s).

Ethyl benzoate (**1b**)

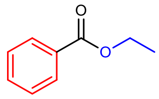

^{1}H NMR (300 MHz, $CDCl_3$) δ 8.10-8.00 (m, 2H), 7.54 (t, J = 7.4 Hz, 1H), 7.42 (t, J = 7.4 Hz, 2H), 4.37 (q, J = 7.1 Hz, 2H), 1.39 (t, J = 7.1 Hz, 3H). ^{13}C NMR (75 MHz, $CDCl_3$) δ 166.88 (s), 133.06 (s), 130.80 (s), 129.80 (s), 128.58 (s), 61.20 (s), 14.60 (s).

Propyl benzoate (**1c**)

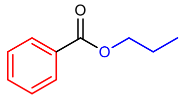

^{1}H NMR (300 MHz, $CDCl_3$) δ 8.05 (d, J = 7.2 Hz, 2H), 7.53 (t, J = 7.4 Hz, 1H), 7.41 (t, J = 7.5 Hz, 2H), 4.27 (t, J = 6.7 Hz, 2H), 1.86-1.71 (m, 2H), 1.02 (t, J = 7.4 Hz, 3H). ^{13}C NMR (75 MHz, $CDCl_3$) δ 166.83 (s), 132.99 (s), 130.76 (s), 129.74 (s), 128.52 (s), 66.70 (s), 22.34 (s), 10.72 (s).

Butyl benzoate (**1d**)

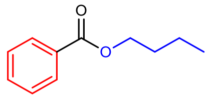

^{1}H NMR (400 MHz, $CDCl_3$) δ 8.04 (d, J = 7.7 Hz, 2H), 7.49 (dd, J = 6.0, 4.2 Hz, 1H), 7.40 (dd, J = 7.0, 3.2 Hz, 2H), 4.35-4.26 (m, 2H), 1.78-1.65 (m, 2H), 1.52-1.38 (m, 2H),

0.96 (t, J = 8.4 Hz, 3H). ^{13}C NMR (101 MHz, $CDCl_3$) δ 166.64 (s), 132.86 (s), 130.73 (s), 129.65 (s), 128.41 (s), 64.85 (s), 30.94 (s), 19.42 (s), 13.85 (s).

Isopropyl benzoate (**1e**)

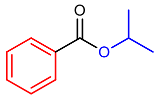

^{1}H NMR (300 MHz, $CDCl_3$) δ 8.04 (dd, J = 8.4, 1.3 Hz, 2H), 7.53 (t, J = 7.4 Hz, 1H), 7.42 (t, J = 7.5 Hz, 2H), 5.26 (dt, J = 12.5, 6.3 Hz, 1H), 1.37 (d, J = 6.3 Hz, 6H). ^{13}C NMR (75 MHz, $CDCl_3$) δ 166.37 (s), 132.96 (s), 131.22 (s), 129.78 (s), 128.53 (s), 68.59 (s), 22.23 (s).

Methyl 4-methoxybenzoate (**1f**)

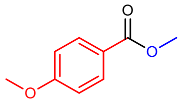

^{1}H NMR (300 MHz, $CDCl_3$) δ 7.99 (d, J = 8.9 Hz, 2H), 6.92 (d, J = 8.9 Hz, 2H), 3.87 (d, J = 8.0 Hz, 6H). ^{13}C NMR (75 MHz, $CDCl_3$) δ 167.20 (s), 163.66 (s), 131.92 (s), 122.95 (s), 113.93 (s), 55.75 (s), 52.19 (s).

Methyl 3-methoxybenzoate (**1g**)

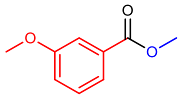

^{1}H NMR (300 MHz, $CDCl_3$) δ 7.62 (d, J = 7.6 Hz, 1H), 7.55 (s, 1H), 7.31 (t, J = 8.0 Hz, 1H), 7.08 (d, J = 9.1 Hz, 1H), 3.89 (s, 3H), 3.81 (s, 3H). ^{13}C NMR (75 MHz, $CDCl_3$) δ 167.10 (s), 159.75 (s), 131.63 (s), 129.56 (s), 122.13 (s), 119.60 (s), 114.17 (s), 55.53 (s), 52.29 (s).

Methyl 2-methoxybenzoate (**1h**)

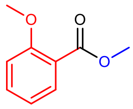

^1H NMR (300 MHz, $CDCl_3$) δ 7.78 (d, J = 7.9 Hz, 1H), 7.45 (t, J = 7.9 Hz, 1H), 6.96 (dd, J = 7.9, 3.7 Hz, 2H), 3.88 (s, 6H). ^{13}C NMR (75 MHz, $CDCl_3$) δ 166.92 (s), 159.30 (s), 133.73 (s), 131.83 (s), 120.26 (d, J = 9.1 Hz), 112.21 (s), 56.15 (s), 52.18 (s).

Methyl 4-methylbenzoate (**1i**)

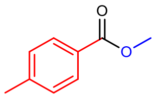

^1H NMR (300 MHz, $CDCl_3$) δ 7.93 (d, J = 8.2 Hz, 2H), 7.24 (d, J = 8.0 Hz, 2H), 3.90 (s, 3H), 2.41 (s, 3H). ^{13}C NMR (75 MHz, $CDCl_3$) δ 167.50 (s), 143.87 (s), 129.92 (s), 129.39 (s), 127.76 (s), 52.26 (s), 21.96 (s).

Methyl 4-cyanobenzoate (**1j**)

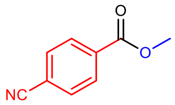

^1H NMR (600 MHz, $CDCl_3$) δ 8.15 (d, J = 8.5 Hz, 2H), 7.75 (d, J = 8.5 Hz, 2H), 3.97 (s, 3H). ^{13}C NMR (151 MHz, $CDCl_3$) δ 165.76 (s), 134.23 (s), 132.55 (s), 130.42 (s), 118.30 (s), 116.70 (s), 53.07 (s).

Methyl 4-fluorobenzoate (**1k**)

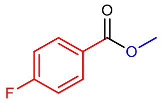

^1H NMR (300 MHz, $CDCl_3$) δ 8.12-7.96 (m, 2H), 7.10 (t, J = 8.4 Hz, 2H), 3.91 (s, 3H). ^{13}C NMR (75 MHz, $CDCl_3$) δ 167.72 (s), 166.40 (s), 164.36 (s), 132.41 (d, J = 9.3 Hz), 126.72 (d, J = 3.0 Hz), 115.93 (s), 115.64 (s), 52.45 (s).

Methyl 4-chlorobenzoate (**1l**)

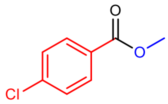

^{1}H NMR (600 MHz, $CDCl_3$) δ 7.97 (d, J = 8.6 Hz, 1H), 7.41 (d, J = 8.6 Hz, 1H), 3.91 (s, 2H).^{13}C NMR (151 MHz, $CDCl_3$) δ 166.56 (s), 139.68 (s), 131.29 (s), 129.03 (s), 128.88 (s), 52.61 (s).

Methyl 4-bromobenzoate (**1m**)

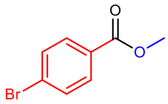

^{1}H NMR (300 MHz, $CDCl_3$) δ 7.90 (d, J = 8.6 Hz, 2H), 7.58 (d, J = 8.5 Hz, 2H), 3.91 (s, 3H). ^{13}C NMR (75 MHz, $CDCl_3$) δ 166.69 (s), 132.05 (s), 131.45 (s), 129.38 (s), 128.37 (s), 52.63 (s).

Methyl 4-iodobenzoate (**1n**)

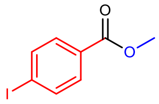

^{1}H NMR (600 MHz, $CDCl_3$) δ 7.80 (d, J = 8.5 Hz, 2H), 7.74 (d, J = 8.5 Hz, 2H), 3.91 (s, 3H). ^{13}C NMR (151 MHz, $CDCl_3$) δ 166.93 (s), 138.04 (s), 131.35 (s), 129.90 (s), 101.07 (s), 52.63 (s).

Methyl 4-(methylsulfonyl)benzoate (**1o**)

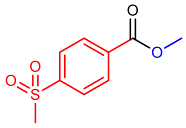

^{1}H NMR (600 MHz, $CDCl_3$) δ 8.24 (d, J = 7.9 Hz, 2H), 8.04 (d, J = 7.9 Hz, 2H), 3.98 (s, 3H), 3.10 (s, 3H). ^{13}C NMR (151 MHz, $CDCl_3$) δ 165.72 (s), 144.50 (s), 135.13 (s), 130.83 (s), 127.79 (s), 53.06 (s), 44.59 (s).

Methyl 4-nitrobenzoate (**1p**)

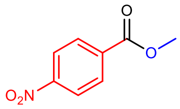

^{1}H NMR (600 MHz, $CDCl_3$) δ 8.30 (d, J = 8.8 Hz, 2H), 8.22 (d, J = 8.8 Hz, 2H), 3.99 (s, 3H). ^{13}C NMR (151 MHz, $CDCl_3$) δ 165.49 (s), 150.82 (s), 135.78 (s), 131.03 (s), 123.86 (s), 53.16 (s).

Methyl 4-trifluoromethylbenzoate (**1q**)

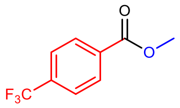

^{1}H NMR (300 MHz, $CDCl_3$) δ 8.15 (d, J = 8.1 Hz, 2H), 7.70 (d, J = 8.2 Hz, 2H), 3.96 (s, 3H). ^{13}C NMR (75 MHz, $CDCl_3$) δ 166.15 (s), 134.95 (s), 134.52 (s), 133.69 (d, J = 1.1 Hz), 130.29 (s), 125.70 (dd, J = 7.5, 3.7 Hz), 122.16 (s), 52.78 (s).

Methyl 4-phenylbenzoate (**1r**)

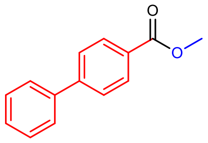

^{1}H NMR (600 MHz, $CDCl_3$) δ 8.12 -8.07 (m, 2H), 7.67-7.63 (m, 2H), 7.61 (dd, J = 8.0, 0.9 Hz, 2H), 7.45 (dd, J = 10.5, 4.7 Hz, 2H), 7.38 (t, J = 7.4 Hz, 1H), 3.92 (s, 3H). ^{13}C NMR (151 MHz, $CDCl_3$) δ 167.28 (s), 145.89 (s), 140.25 (s), 130.39 (s), 129.18 (d, J = 9.7 Hz), 128.43 (s), 127.55 (s), 127.32 (s), 52.42 (s).

Mthetyl 2-naphthoate (**1s**)

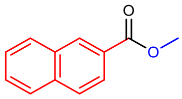

^{1}H NMR (600 MHz, $CDCl_3$) δ 8.61 (s, 1H), 8.06 (dd, J = 8.6, 1.5 Hz, 1H), 7.94 (d, J = 8.1 Hz, 1H), 7.87 (dd, J = 8.3, 2.7 Hz, 2H), 7.61-7.56 (m, 1H), 7.56-7.51 (m, 1H), 3.98 (s, 3H). ^{13}C NMR (151 MHz, $CDCl_3$) δ 167.58 (s), 135.80 (s), 132.78 (s), 131.37 (s), 129.65 (s), 128.50 (d, J = 12.8 Hz), 128.06 (s), 127.67 (s), 126.94 (s), 125.52 (s), 52.55 (s).

Methyl 1,3-benzodioxole-5-carboxylate (**1t**)

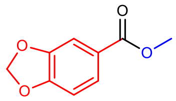

^{1}H NMR (600 MHz, $CDCl_3$) δ 7.64 (dd, J = 8.2, 1.7 Hz, 1H), 7.45 (d, J = 1.6 Hz, 1H), 6.83 (d, J = 8.2 Hz, 1H), 6.03 (s, 2H), 3.87 (s, 3H). ^{13}C NMR (151 MHz, $CDCl_3$) δ 166.70 (s), 151.82 (s), 147.94 (s), 125.54 (s), 124.36 (s), 109.71 (s), 108.18 (s), 102.05 (s), 52.29 (s).

Methyl 1,4-Benzodioxan-6-carboxylate (**1u**)

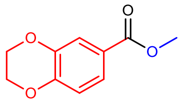

^{1}H NMR (600 MHz, $CDCl_3$) δ 7.64 – 7.50 (m, 2H), 6.88 (d, J = 8.1 Hz, 1H), 4.32-4.29 (m, 2H), 4.28-4.24 (m, 2H), 3.87 (s, 3H). ^{13}C NMR (151 MHz, $CDCl_3$) δ 166.86 (s), 148.00 (s), 143.39 (s), 123.62 (d, J = 2.1 Hz), 119.22 (s), 117.34 (s), 64.85 (s), 64.31 (s), 52.19 (s).

Methyl benzo[b]furan-2-carboxylate (**1v**)

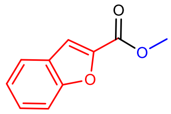

^{1}H NMR (600 MHz, $CDCl_3$) δ 7.68 (d, J = 7.9 Hz, 1H), 7.59 (dd, J = 8.4, 0.7 Hz, 1H), 7.53 (d, J = 0.8 Hz, 1H), 7.45 (ddd, J = 8.4, 7.3, 1.2 Hz, 1H), 7.33-7.28 (m, 1H), 3.98 (s, 3H). ^{13}C NMR (151 MHz, $CDCl_3$) δ 160.29 (s), 155.99 (s), 145.65 (s), 127.99 (s), 127.20 (s), 124.12 (s), 123.15 (s), 114.32 (s), 112.65 (s), 52.70 (s).

Methyl isonicotinate (**1w**)

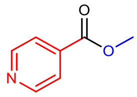

^{1}H NMR (400 MHz, $CDCl_3$) δ 8.51 (d, J = 6.0 Hz, 2H), 7.55 (d, J = 6.0 Hz, 2H), 3.68 (s, 3H). ^{13}C NMR (101 MHz, $CDCl_3$) δ 165.19 (s), 150.38 (s), 136.97 (s), 122.52 (s), 52.34 (s).

2-(Methoxycarbonyl)thiophene (**1x**)

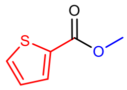

^{1}H NMR (400 MHz, $CDCl_3$) δ 7.78 (d, J = 2.2 Hz, 1H), 7.53 (t, J = 4.7 Hz, 1H), 7.12-6.99 (m, 1H), 3.92-3.79 (m, 3H). ^{13}C NMR (101 MHz, $CDCl_3$) δ 162.62 (s), 133.55 (d, J = 17.7 Hz), 132.42 (s), 127.79 (s), 52.08 (d, J = 1.5 Hz).

Methyl 2-furoate (**1y**)

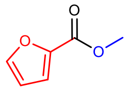

^{1}H NMR (400 MHz, $CDCl_3$) δ 7.61 (d, J = 0.8 Hz, 1H), 7.18 (d, J = 3.5 Hz, 1H), 6.52 (dd, J = 3.5, 1.7 Hz, 1H), 3.89 (s, 3H). ^{13}C NMR (101 MHz, $CDCl_3$) δ 158.89 (s), 146.27 (s), 144.51 (s), 117.75 (s), 111.72 (s), 51.60 (s).

Dimethyl phthalate (**2a**)

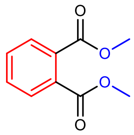

^{1}H NMR (400 MHz, $CDCl_3$) δ 7.68 (dd, J = 9.0, 4.8 Hz, 1H), 7.49 (dd, J = 9.0, 3.4 Hz, 1H), 3.87 (dd, J = 10.2, 4.4 Hz, 3H). ^{13}C NMR (101 MHz, $CDCl_3$) δ 167.90 (s), 131.93 (s), 131.08 (s), 128.80 (s), 52.46 (s).

Methyl cinnamate (**2b**)

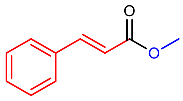

^{1}H NMR (400 MHz, $CDCl_3$) δ 7.69 (d, J = 16.0 Hz, 1H), 7.51 (dd, J = 6.5, 3.0 Hz, 2H), 7.40-7.31 (m, 3H), 6.44 (d, J = 16.0 Hz, 1H), 3.79 (s, 3H). ^{13}C NMR (101 MHz, $CDCl_3$) δ 167.64 (s), 145.11 (s), 134.67 (s), 130.54 (s), 129.15 (s), 128.33 (s), 118.09 (s), 51.91 (s).

Methyl 3-(2-furyl)acrylate (**2c**)

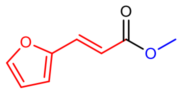

^{1}H NMR (600 MHz, $CDCl_3$) δ 7.48 (s, 1H), 7.44 (d, J = 15.7 Hz, 1H), 6.61 (d, J = 3.3 Hz, 1H), 6.47 (dd, J = 3.1, 1.7 Hz, 1H), 6.32 (d, J = 15.7 Hz, 1H), 3.79 (s, 3H). ^{13}C NMR (151 MHz, $CDCl_3$) δ 167.83 (s), 151.17 (s), 145.07 (s), 131.53 (s), 115.72 (s), 115.16 (s), 112.59 (s), 52.00 (s).

Methyl 3-(2,6,6-trimethyl-1-cyclohexen-1-yl)-2-propenoate (**2d**)

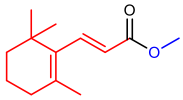

^{1}H NMR (600 MHz, $CDCl_3$) δ 6.80 (dd, J = 15.5, 9.9 Hz, 1H), 5.80 (dd, J = 15.5, 0.6 Hz, 1H), 5.49 (s, 1H), 3.73 (s, 3H), 2.28 (d, J = 9.8 Hz, 1H), 2.03 (s, 2H), 1.57 (d, J = 1.7 Hz, 3H), 1.51-1.43 (m, 1H), 1.20 (dt, J = 13.4, 4.7 Hz, 1H), 0.92 (s, 3H), 0.85 (s, 3H). ^{13}C NMR (151 MHz, $CDCl_3$) δ 167.27 (s), 150.55 (s), 132.17 (s), 122.87 (s), 122.17 (s), 54.33 (s), 51.71 (s), 32.72 (s), 31.38 (s), 28.06 (s), 27.06 (s), 23.31 (s), 23.07 (s).

Methyl 3-(2,6,6-trimethyl-2-cyclohexen-1-yl)-2-propenoate (**2e**)

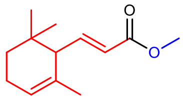

^{1}H NMR (600 MHz, $CDCl_3$) δ 7.43 (d, J = 16.2 Hz, 1H), 5.83 (d, J = 16.2 Hz, 1H), 3.76 (s, 3H), 2.06 (t, J = 6.3 Hz, 2H), 1.76 (s, 3H), 1.64-1.60 (m, 2H), 1.49-1.46 (m, 2H), 1.06 (s, 6H). ^{13}C NMR (151 MHz, $CDCl_3$) δ 168.10 (s), 144.90 (s), 136.16 (s), 135.87 (s), 121.73 (s), 51.79 (s), 40.04 (s), 34.34 (s), 33.80 (s), 29.06 (s), 21.99 (s), 19.24 (s).

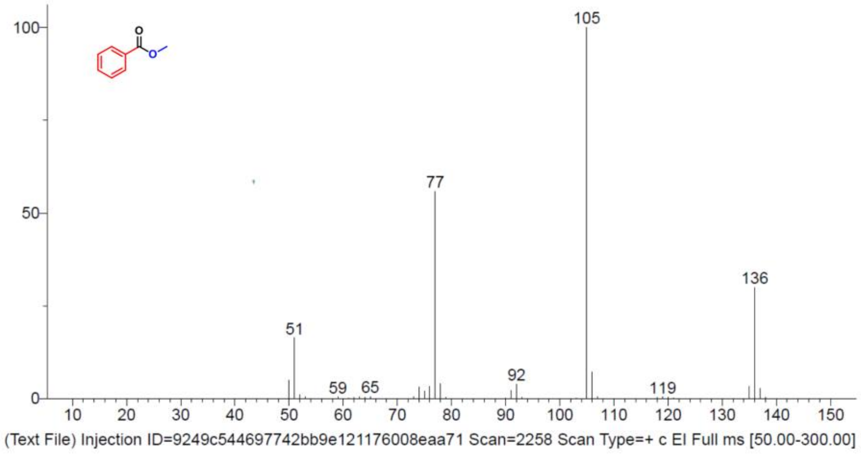
100
50
0
105
77
136
51
59
65
92
119
10 20 30 40 50 60 70 80 90 100 110 120 130 140 150
(Text File) Injection ID=9249c544697742bb9e121176008eaa71 Scan=2258 Scan Type=+ c EI Full ms [50.00-300.00]

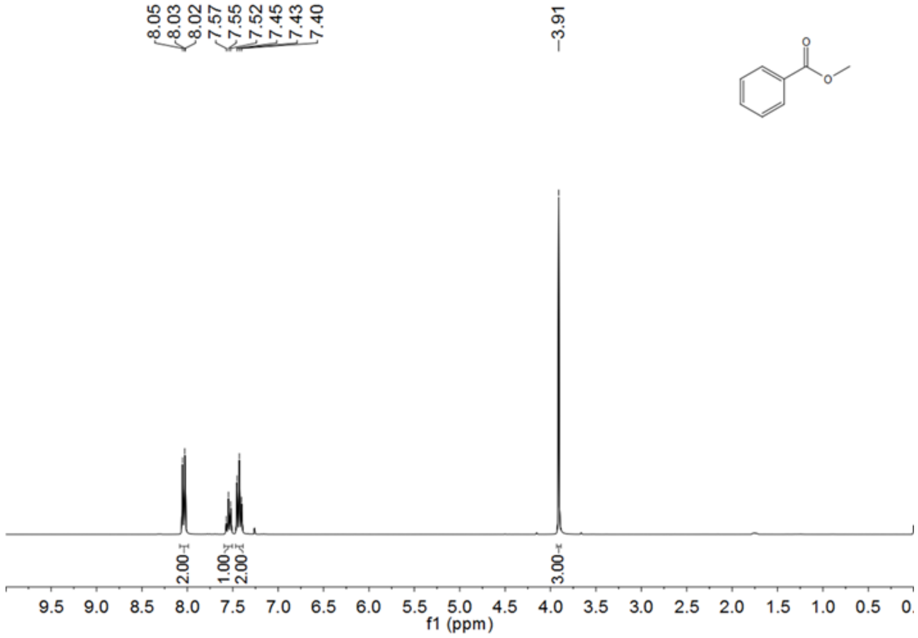
8.05
8.03
8.02
7.57
7.55
7.52
7.45
7.43
7.40
3.91
2.00
1.00
2.00
3.00
9.5 9.0 8.5 8.0 7.5 7.0 6.5 6.0 5.5 5.0 4.5 4.0 3.5 3.0 2.5 2.0 1.5 1.0 0.5 0.
f1 (ppm)

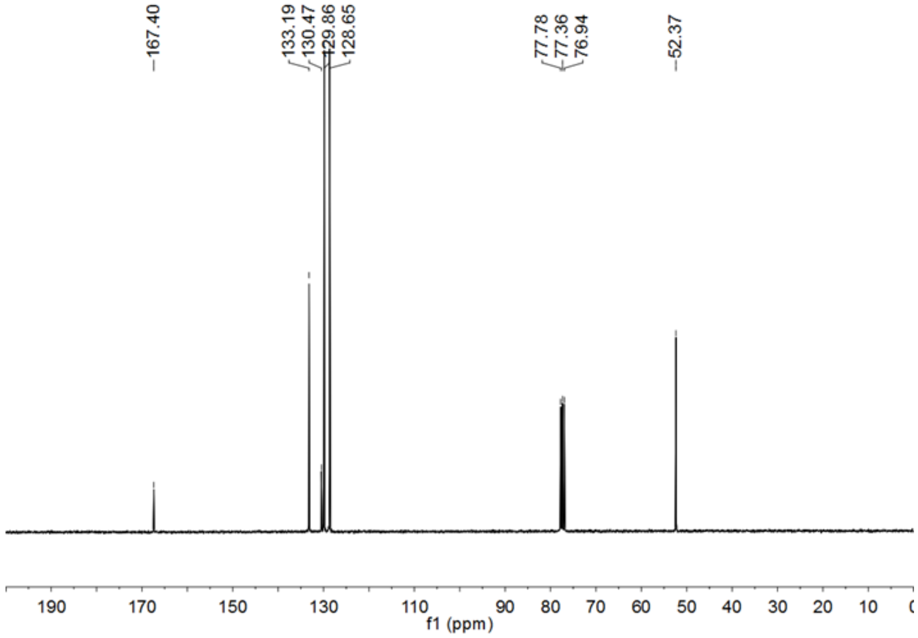
167.40
133.19
130.47
129.86
128.65
77.78
77.36
76.94
52.37
190 170 150 130 110 90 80 70 60 50 40 30 20 10
f1 (ppm)

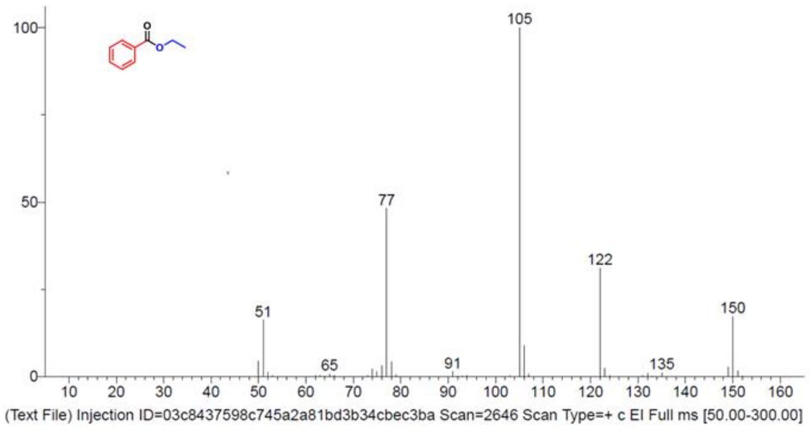
100
50
0
105
77
122
51
150
65
91
135
10 20 30 40 50 60 70 80 90 100 110 120 130 140 150 160
(Text File) Injection ID=03c8437598c745a2a81bd3b34cbec3ba Scan=2646 Scan Type=+ c EI Full ms [50.00-300.00]

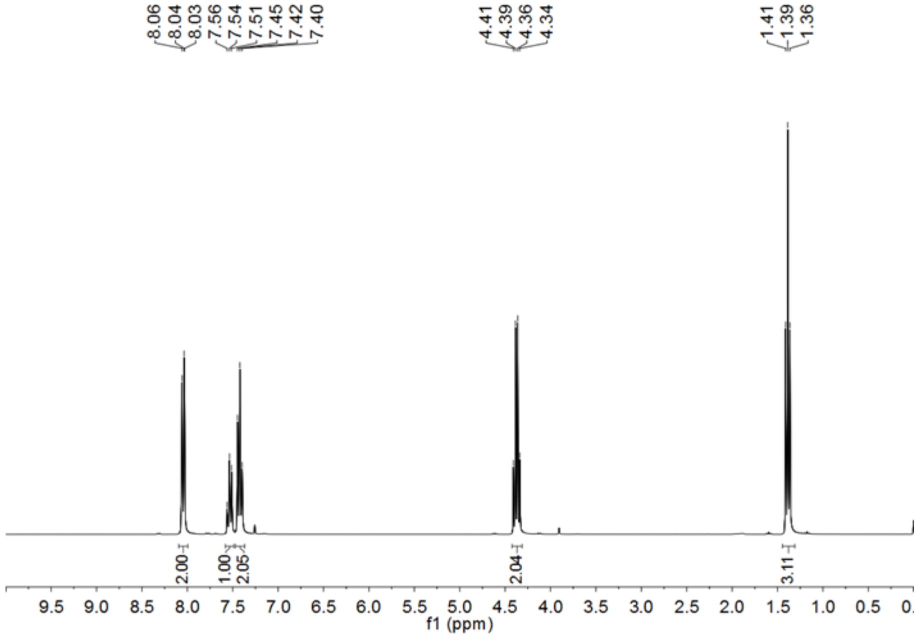
8.06
8.04
8.03
7.56
7.54
7.51
7.45
7.42
7.40
4.41
4.39
4.36
4.34
1.41
1.39
1.36
2.00
1.00
2.05
2.04
3.11
9.5 9.0 8.5 8.0 7.5 7.0 6.5 6.0 5.5 5.0 4.5 4.0 3.5 3.0 2.5 2.0 1.5 1.0 0.5 0.
f1 (ppm)

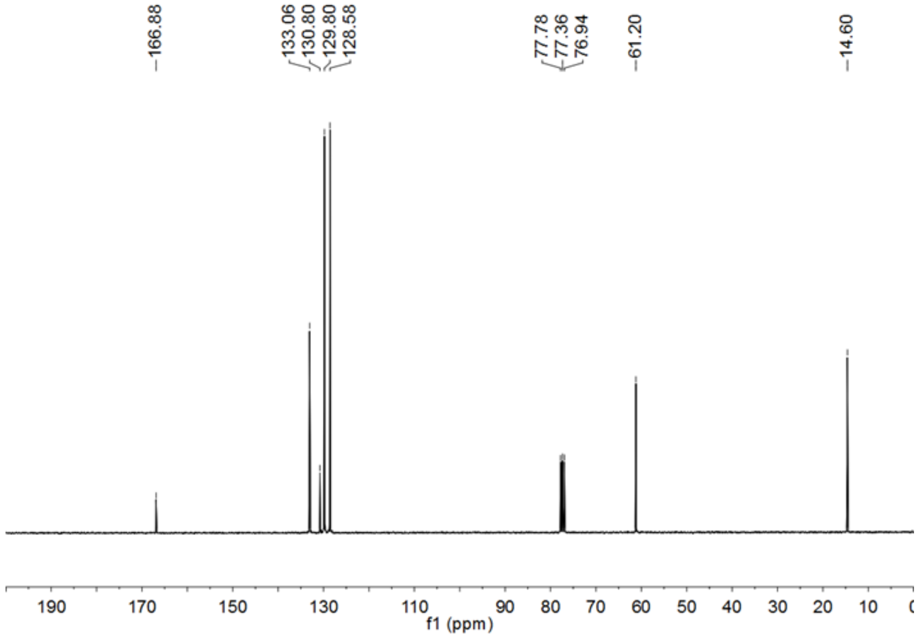
166.88
133.06
130.80
129.80
128.58
77.78
77.36
76.94
61.20
14.60
190 170 150 130 110 90 80 70 60 50 40 30 20 10
f1 (ppm)

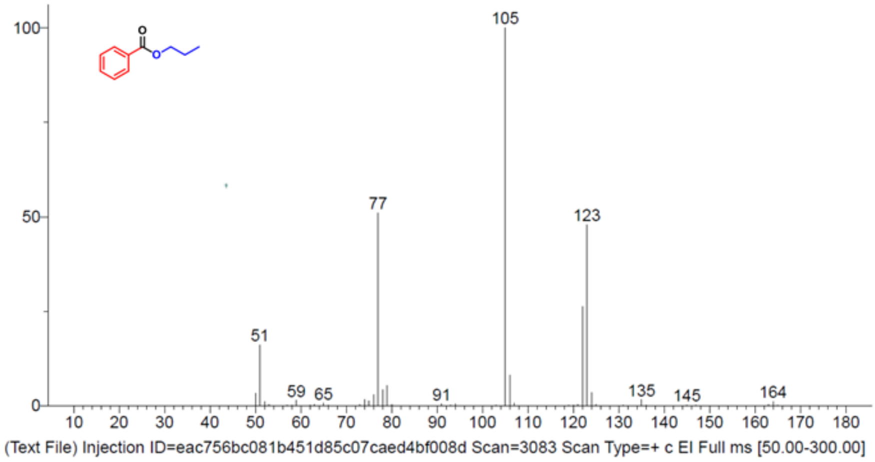
100
50
0
105
77
123
51
59 65
91
135 145
164
10 20 30 40 50 60 70 80 90 100 110 120 130 140 150 160 170 180
(Text File) Injection ID=eac756bc081b451d85c07caed4bf008d Scan=3083 Scan Type=+ c EI Full ms [50.00-300.00]

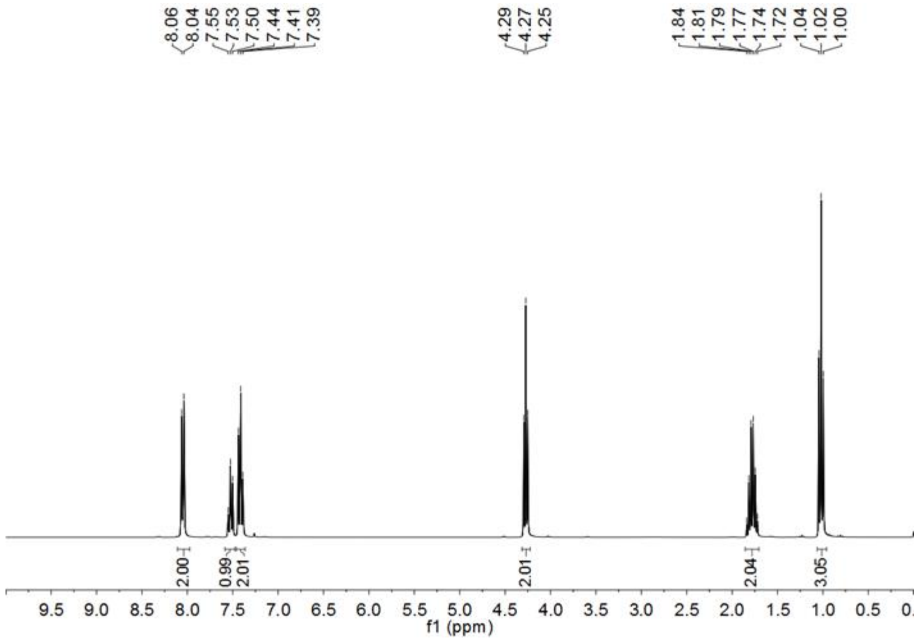
8.06
8.04
7.55
7.53
7.50
7.44
7.41
7.39
4.29
4.27
4.25
1.84
1.81
1.79
1.77
1.74
1.72
1.04
1.02
1.00
2.00
0.99
2.01
2.01
2.04
3.05
9.5 9.0 8.5 8.0 7.5 7.0 6.5 6.0 5.5 5.0 4.5 4.0 3.5 3.0 2.5 2.0 1.5 1.0 0.5
f1 (ppm)

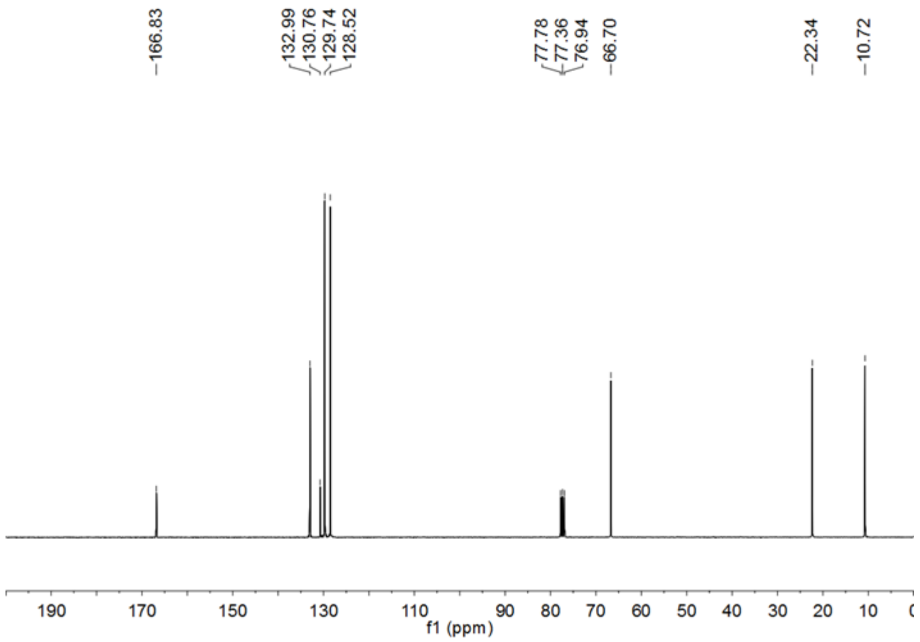
166.83
132.99
130.76
129.74
128.52
77.78
77.36
76.94
66.70
22.34
10.72
190 170 150 130 110 90 80 70 60 50 40 30 20 10
f1 (ppm)

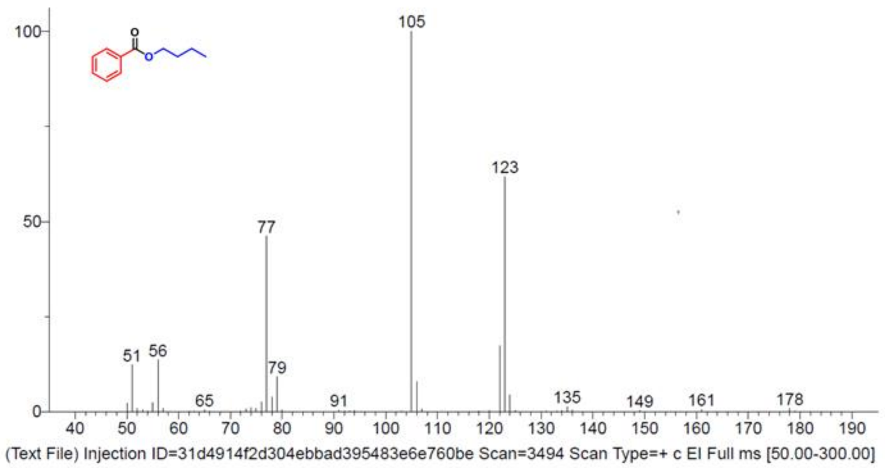
100
50
0
105
123
77
51
56
79
65
91
135
149
161
178
40 50 60 70 80 90 100 110 120 130 140 150 160 170 180 190
(Text File) Injection ID=31d4914f2d304ebbad395483e6e760be Scan=3494 Scan Type=+ c EI Full ms [50.00-300.00]

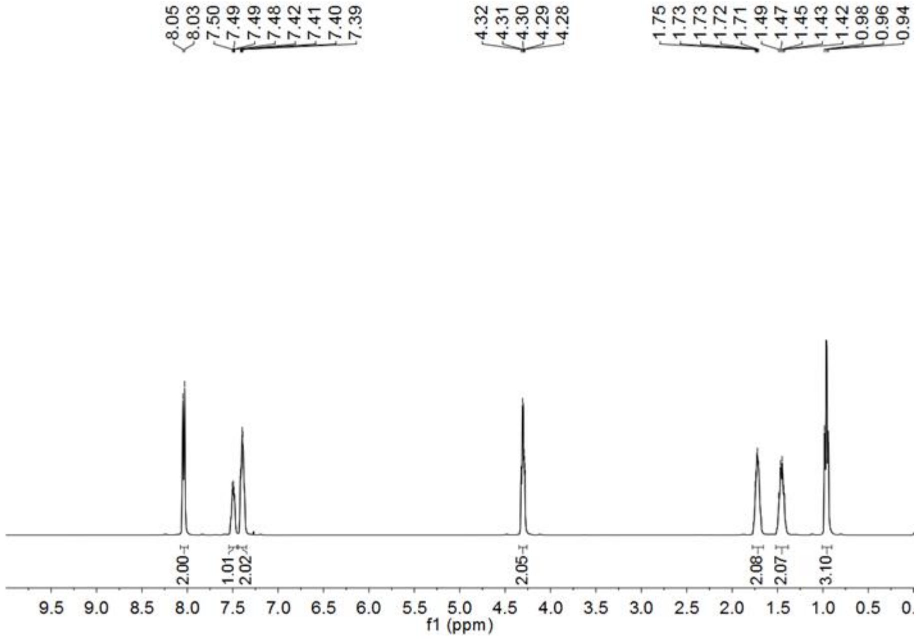
8.05 8.03 7.50 7.49 7.49 7.48 7.42 7.41 7.40 7.39
4.32 4.31 4.30 4.29 4.28
1.75 1.73 1.73 1.72 1.71 1.49 1.47 1.45 1.43 1.42 0.98 0.96 0.94
2.00
1.01
2.02
2.05
2.08
2.07
3.10
9.5 9.0 8.5 8.0 7.5 7.0 6.5 6.0 5.5 5.0 4.5 4.0 3.5 3.0 2.5 2.0 1.5 1.0 0.5
f1 (ppm)

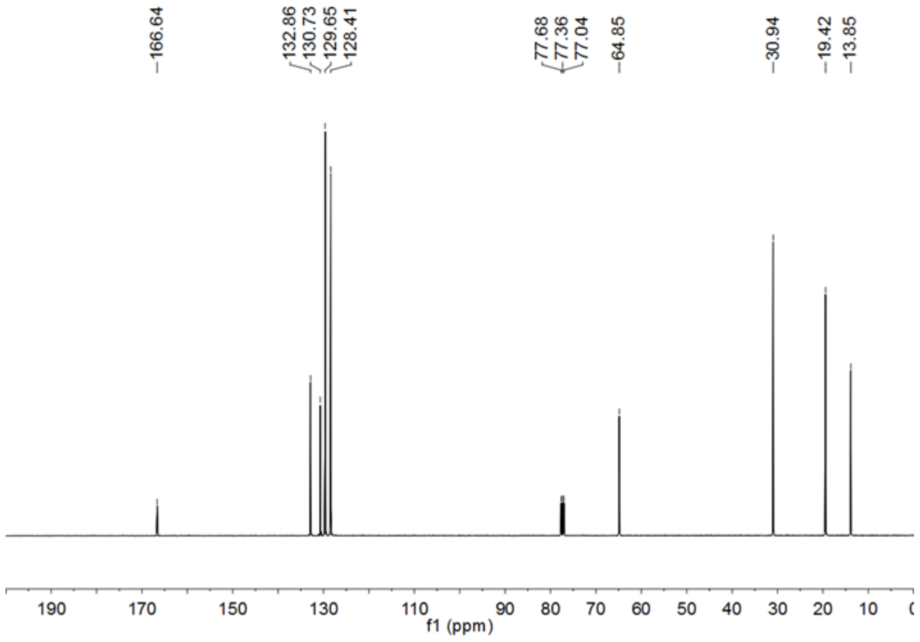
166.64
132.86 130.73 129.65 128.41
77.68 77.36 77.04
64.85
30.94
19.42
13.85
190 170 150 130 110 90 80 70 60 50 40 30 20 10
f1 (ppm)

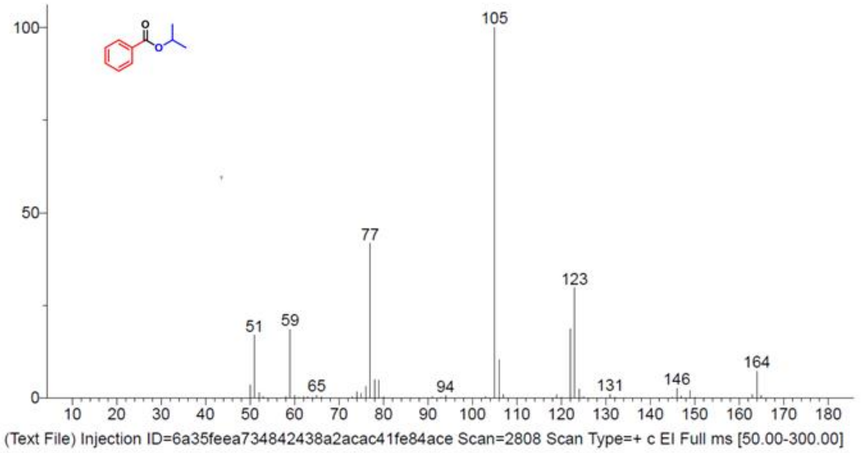

100
50
0
105
77
123
51
59
65
94
131
146
164
10 20 30 40 50 60 70 80 90 100 110 120 130 140 150 160 170 180
(Text File) Injection ID=6a35feea734842438a2acac41fe84ace Scan=2808 Scan Type=+ c EI Full ms [50.00-300.00]

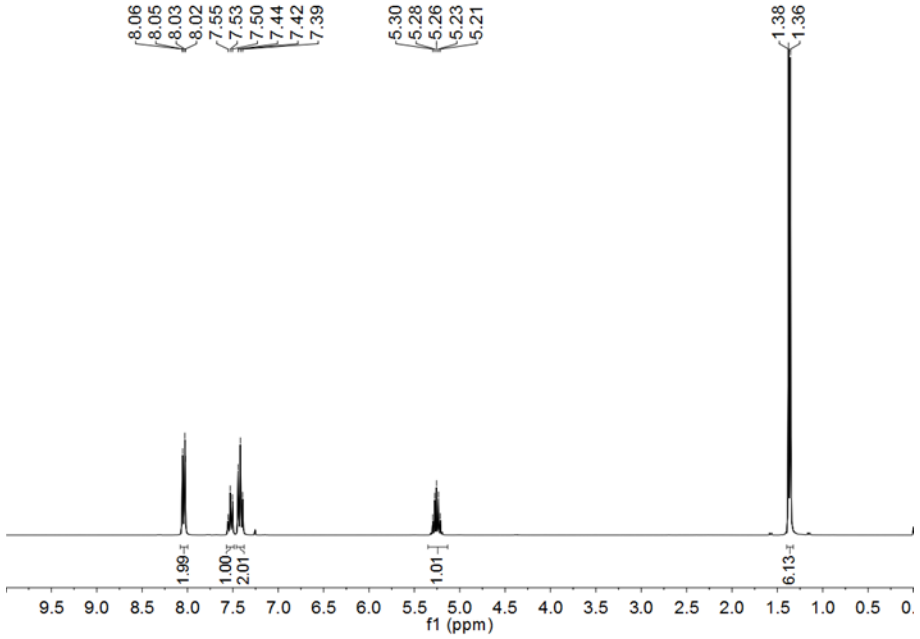

8.06 8.05 8.03 8.02 7.55 7.53 7.50 7.44 7.42 7.39
5.30 5.28 5.26 5.23 5.21
1.38 1.36
1.99
1.00
2.01
1.01
6.13
9.5 9.0 8.5 8.0 7.5 7.0 6.5 6.0 5.5 5.0 4.5 4.0 3.5 3.0 2.5 2.0 1.5 1.0 0.5
f1 (ppm)

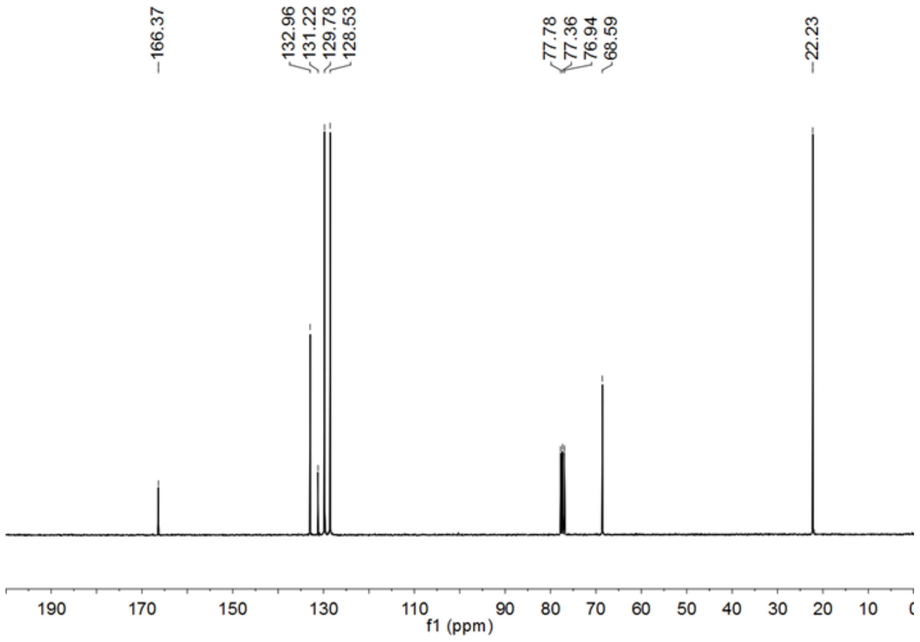

166.37
132.96 131.22 129.78 128.53
77.78 77.36 76.94 68.59
22.23
190 170 150 130 110 90 80 70 60 50 40 30 20 10
f1 (ppm)

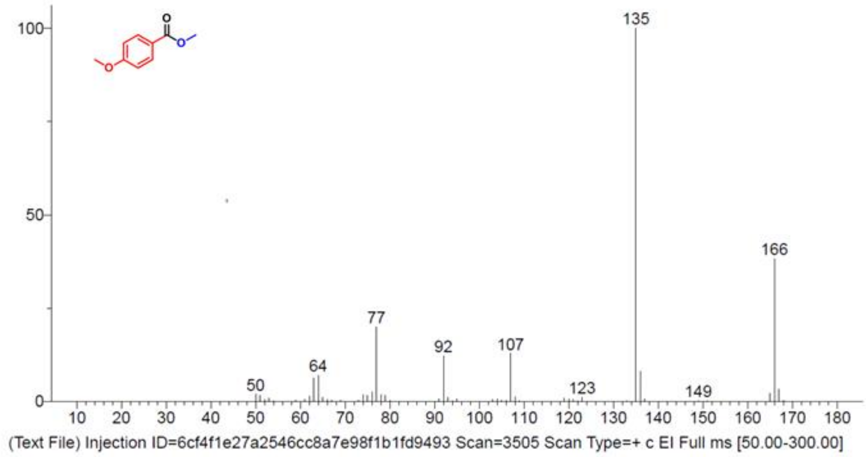
135
166
77
92
107
64
50
123
149
100
50
0
10 20 30 40 50 60 70 80 90 100 110 120 130 140 150 160 170 180
(Text File) Injection ID=6cf4f1e27a2546cc8a7e98f1b1fd9493 Scan=3505 Scan Type=+ c EI Full ms [50.00-300.00]

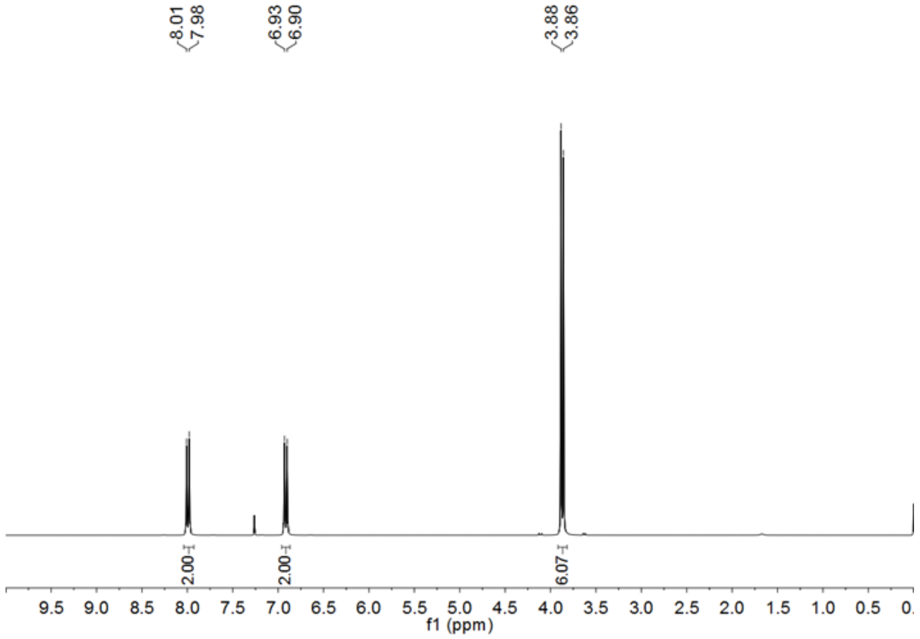
8.01
7.98
6.93
6.90
3.88
3.86
2.00
2.00
6.07
9.5 9.0 8.5 8.0 7.5 7.0 6.5 6.0 5.5 5.0 4.5 4.0 3.5 3.0 2.5 2.0 1.5 1.0 0.5 0.
f1 (ppm)

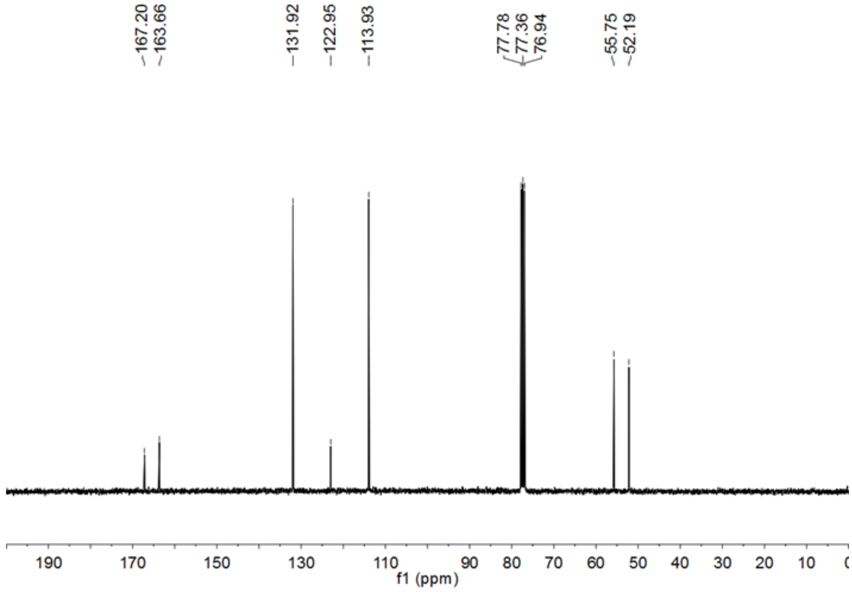
167.20
163.66
131.92
122.95
113.93
77.78
77.36
76.94
55.75
52.19
190 170 150 130 110 90 80 70 60 50 40 30 20 10
f1 (ppm)

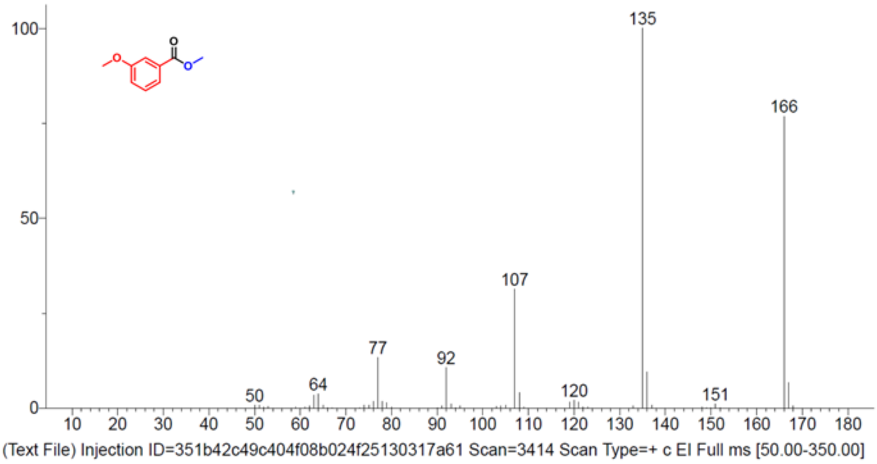
100
50
0
135
166
107
77
92
64
50
120
151
10 20 30 40 50 60 70 80 90 100 110 120 130 140 150 160 170 180
(Text File) Injection ID=351b42c49c404f08b024f25130317a61 Scan=3414 Scan Type=+ c EI Full ms [50.00-350.00]

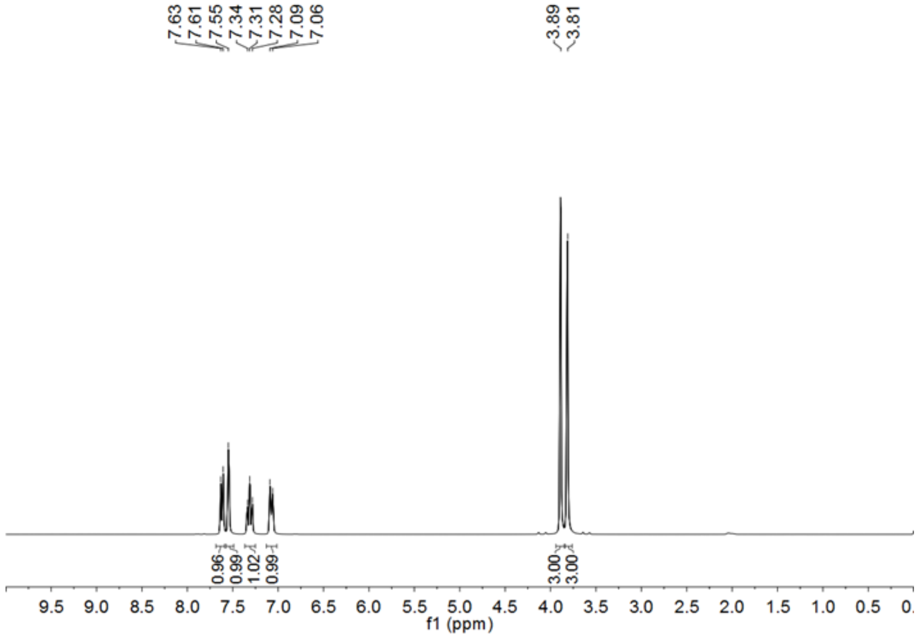
7.63
7.61
7.55
7.34
7.31
7.28
7.09
7.06
3.89
3.81
0.96
0.99
1.02
0.99
3.00
3.00
9.5 9.0 8.5 8.0 7.5 7.0 6.5 6.0 5.5 5.0 4.5 4.0 3.5 3.0 2.5 2.0 1.5 1.0 0.5 0.
f1 (ppm)

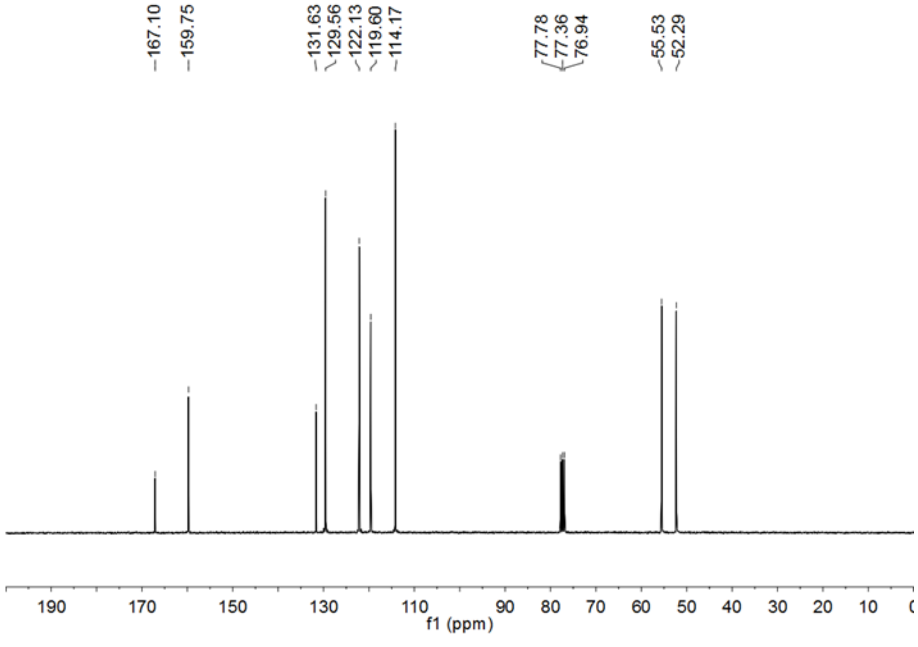
167.10
159.75
131.63
129.56
122.13
119.60
114.17
77.78
77.36
76.94
55.53
52.29
190 170 150 130 110 90 80 70 60 50 40 30 20 10
f1 (ppm)

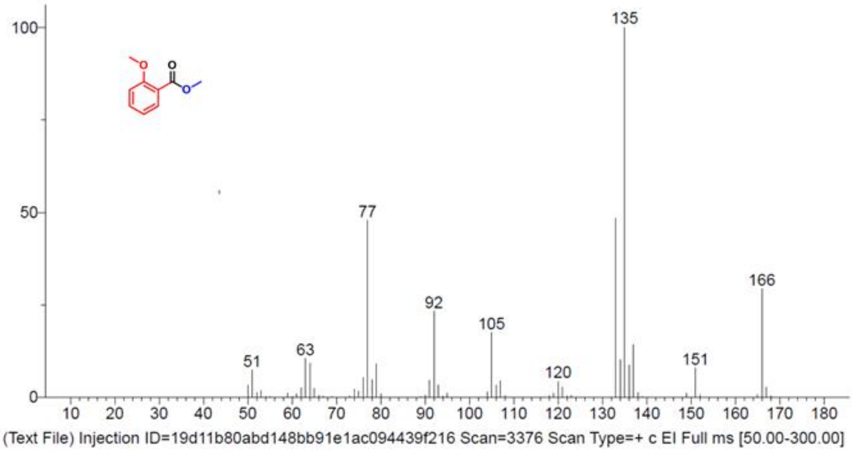

100
50
0
135
77
166
92
105
63
51
151
120
10 20 30 40 50 60 70 80 90 100 110 120 130 140 150 160 170 180
(Text File) Injection ID=19d11b80abd148bb91e1ac094439f216 Scan=3376 Scan Type=+ c EI Full ms [50.00-300.00]

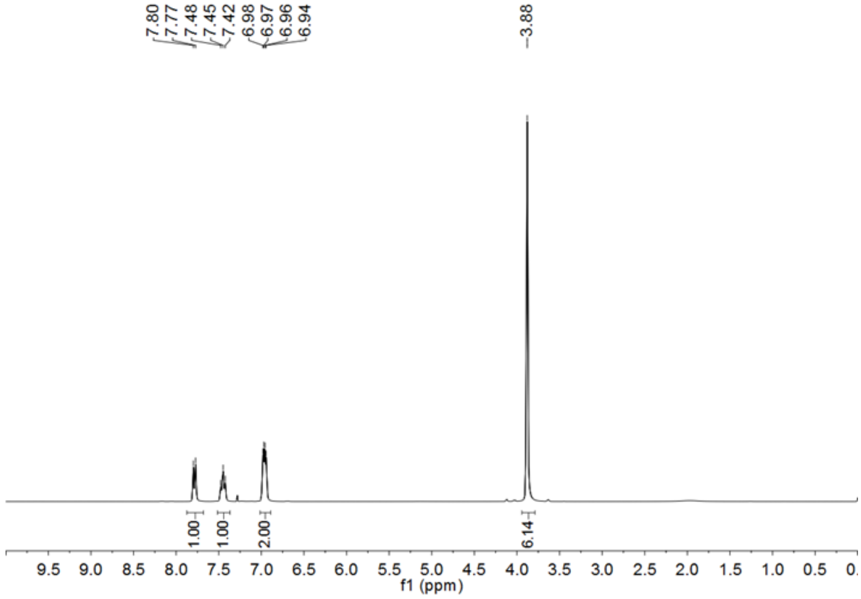

7.80
7.77
7.48
7.45
7.42
6.98
6.97
6.96
6.94
3.88
1.00
1.00
2.00
6.14
9.5 9.0 8.5 8.0 7.5 7.0 6.5 6.0 5.5 5.0 4.5 4.0 3.5 3.0 2.5 2.0 1.5 1.0 0.5 0.
f1 (ppm)

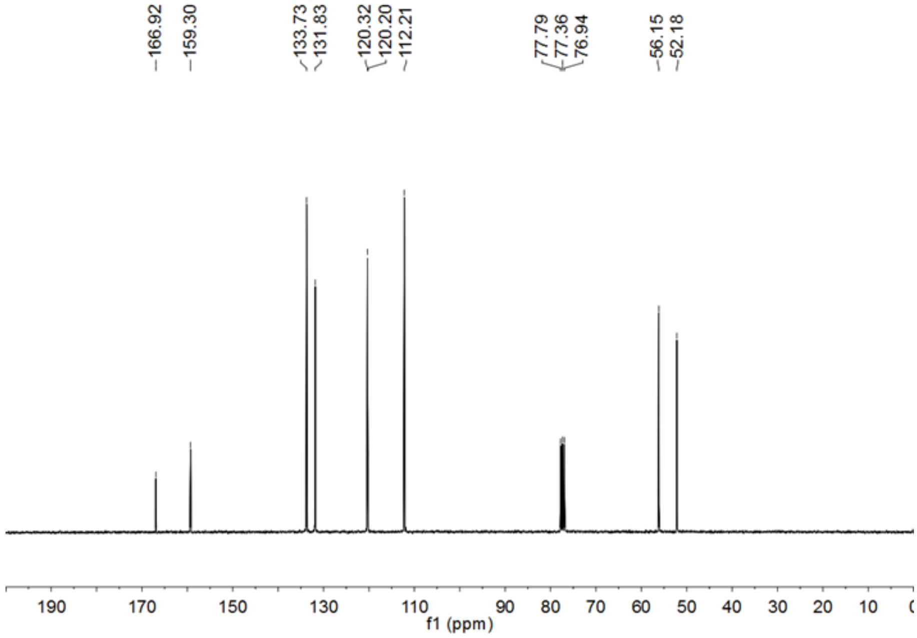

166.92
159.30
133.73
131.83
120.32
120.20
112.21
77.79
77.36
76.94
56.15
52.18
190 170 150 130 110 90 80 70 60 50 40 30 20 10
f1 (ppm)

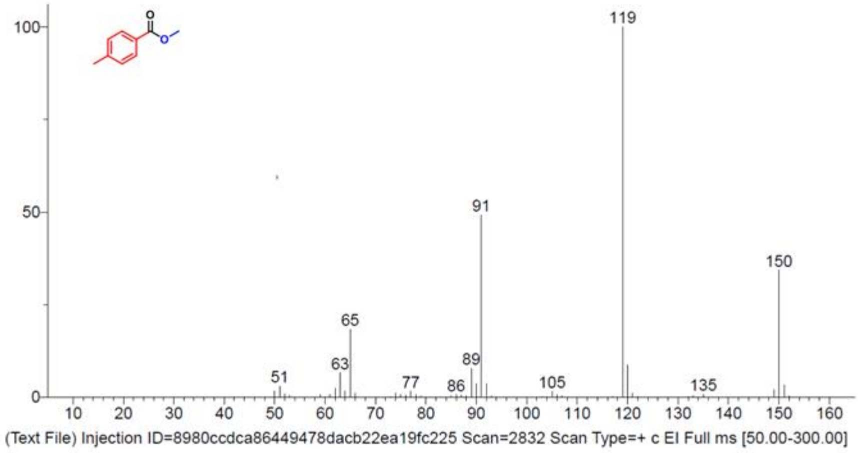

119
91
150
65
63
89
51
77
86
105
135
0 10 20 30 40 50 60 70 80 90 100 110 120 130 140 150 160
(Text File) Injection ID=8980ccdca86449478dacb22ea19fc225 Scan=2832 Scan Type=+ c EI Full ms [50.00-300.00]

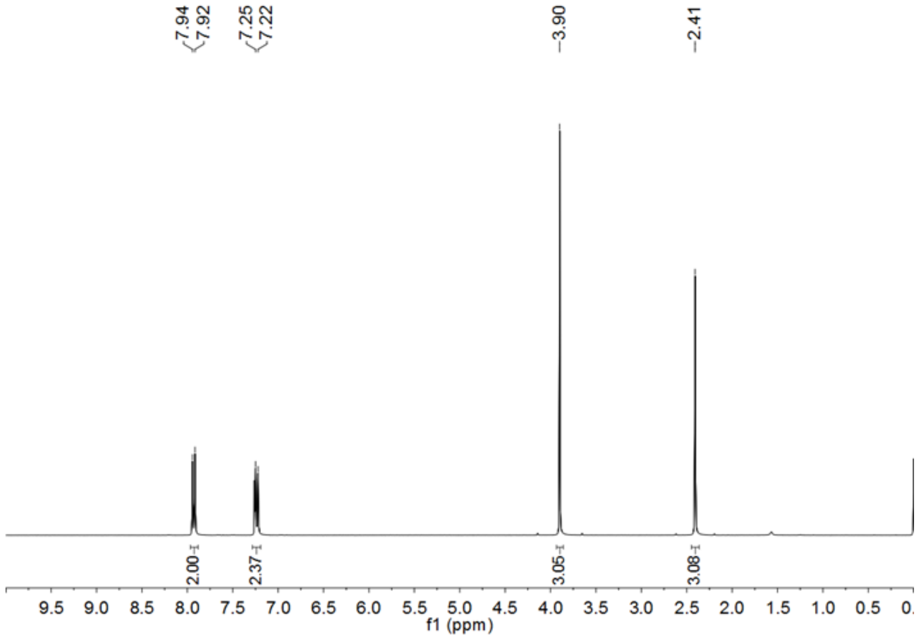

7.94
7.92
7.25
7.22
3.90
2.41
2.00
2.37
3.05
3.08
9.5 9.0 8.5 8.0 7.5 7.0 6.5 6.0 5.5 5.0 4.5 4.0 3.5 3.0 2.5 2.0 1.5 1.0 0.5 0.
f1 (ppm)

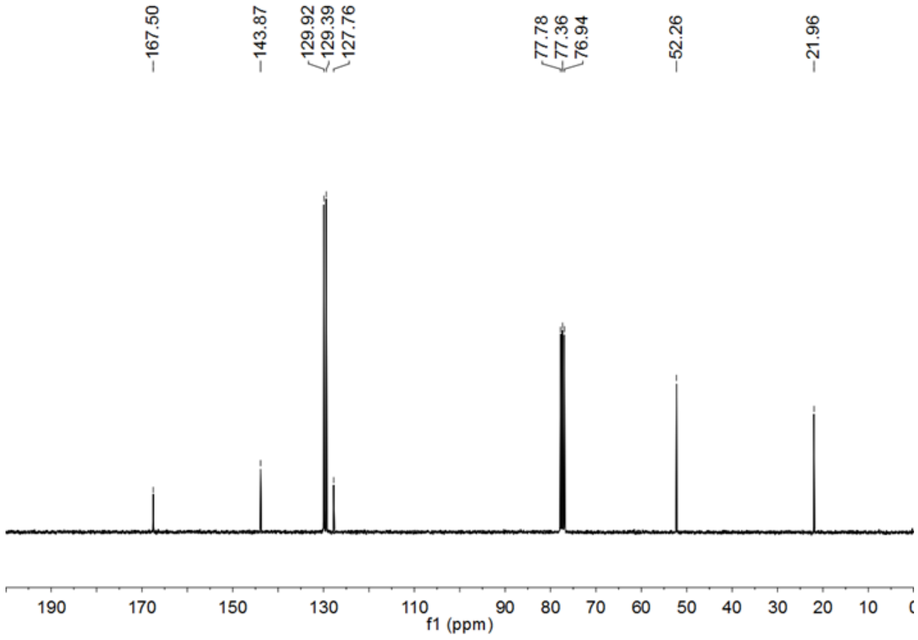

167.50
143.87
129.92
129.39
127.76
77.78
77.36
76.94
52.26
21.96
190 170 150 130 110 90 80 70 60 50 40 30 20 10
f1 (ppm)

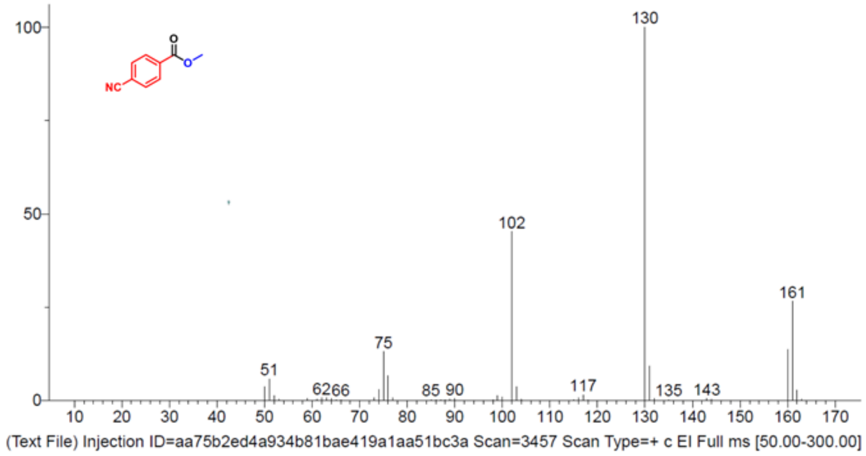
NC
O
O
100
50
0
130
102
161
75
51
6266
85 90
117
135 143
10 20 30 40 50 60 70 80 90 100 110 120 130 140 150 160 170
(Text File) Injection ID=aa75b2ed4a934b81bae419a1aa51bc3a Scan=3457 Scan Type=+ c EI Full ms [50.00-300.00]

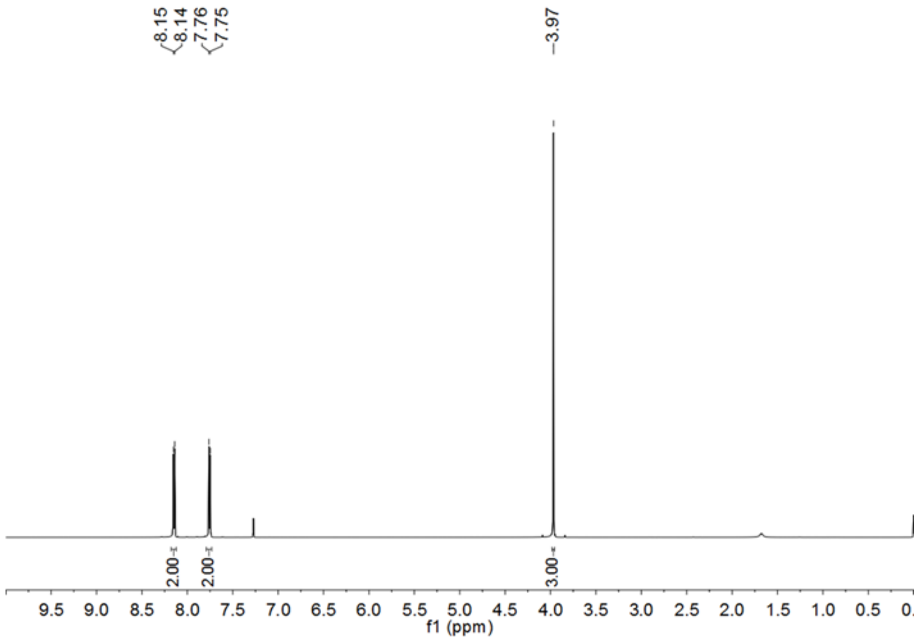
8.15
8.14
7.76
7.75
3.97
2.00
2.00
3.00
9.5 9.0 8.5 8.0 7.5 7.0 6.5 6.0 5.5 5.0 4.5 4.0 3.5 3.0 2.5 2.0 1.5 1.0 0.5 0.
f1 (ppm)

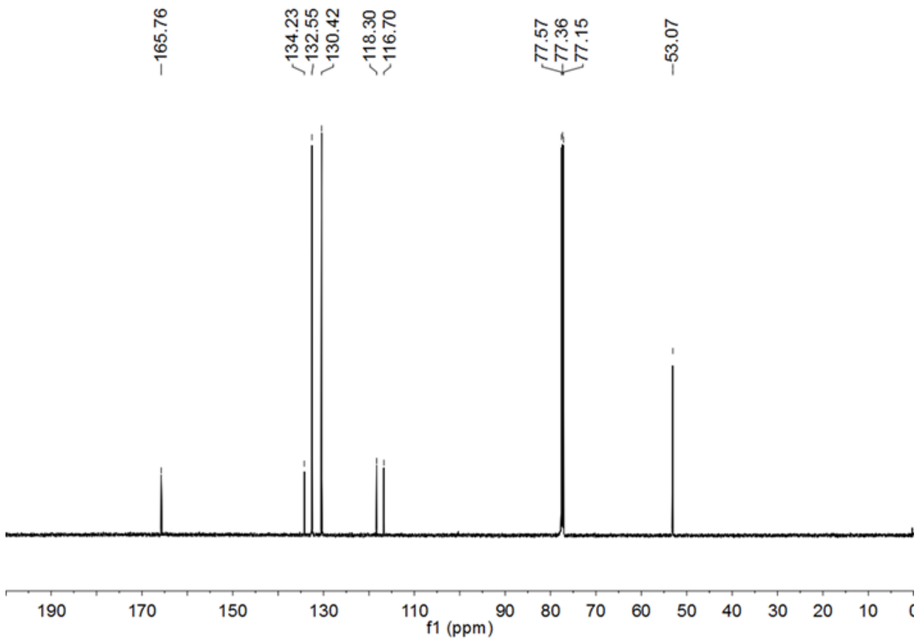
165.76
134.23
132.55
130.42
118.30
116.70
77.57
77.36
77.15
53.07
190 170 150 130 110 90 80 70 60 50 40 30 20 10
f1 (ppm)

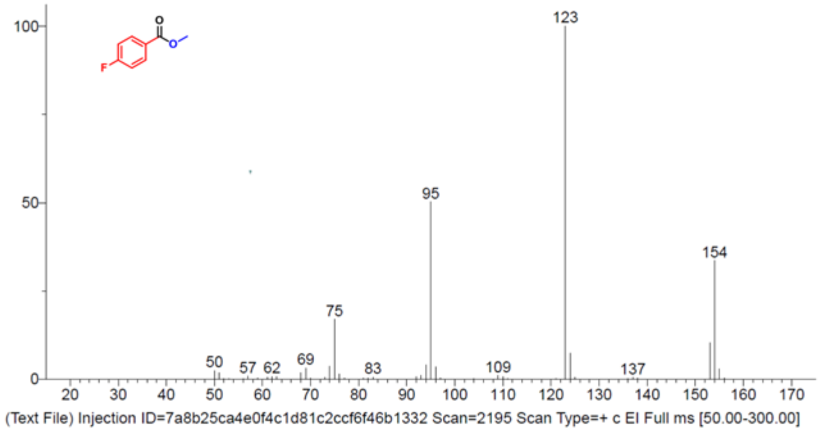
100
50
0
123
95
154
75
50
57
62
69
83
109
137
F
O
O
20
30
40
50
60
70
80
90
100
110
120
130
140
150
160
170
(Text File) Injection ID=7a8b25ca4e0f4c1d81c2ccf6f46b1332 Scan=2195 Scan Type=+ c EI Full ms [50.00-300.00]

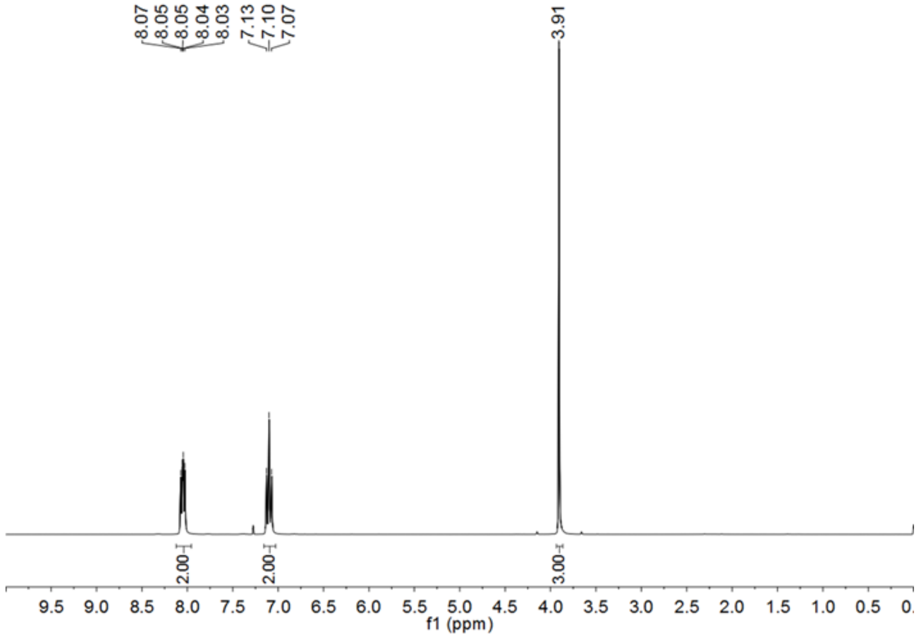
8.07
8.05
8.05
8.04
8.03
7.13
7.10
7.07
3.91
2.00
2.00
3.00
9.5
9.0
8.5
8.0
7.5
7.0
6.5
6.0
5.5
5.0
4.5
4.0
3.5
3.0
2.5
2.0
1.5
1.0
0.5
f1 (ppm)

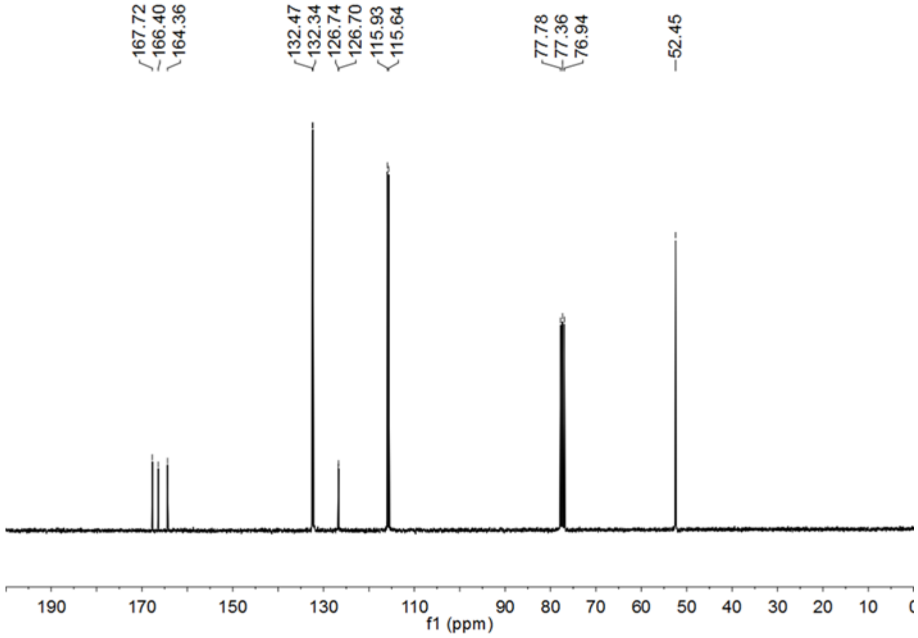
167.72
166.40
164.36
132.47
132.34
126.74
126.70
115.93
115.64
77.78
77.36
76.94
52.45
190
170
150
130
110
90
80
70
60
50
40
30
20
10
f1 (ppm)

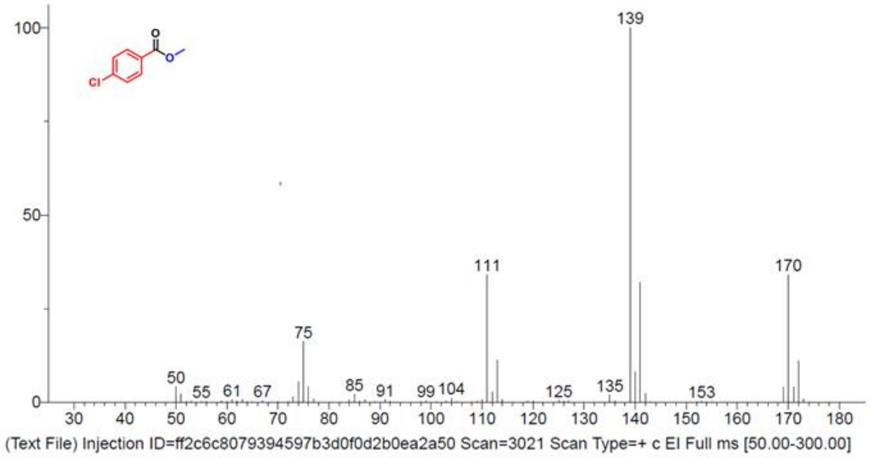
139
111
170
75
50
55
61
67
85
91
99
104
125
135
153
(Text File) Injection ID=ff2c6c8079394597b3d0f0d2b0ea2a50 Scan=3021 Scan Type=+ c EI Full ms [50.00-300.00]

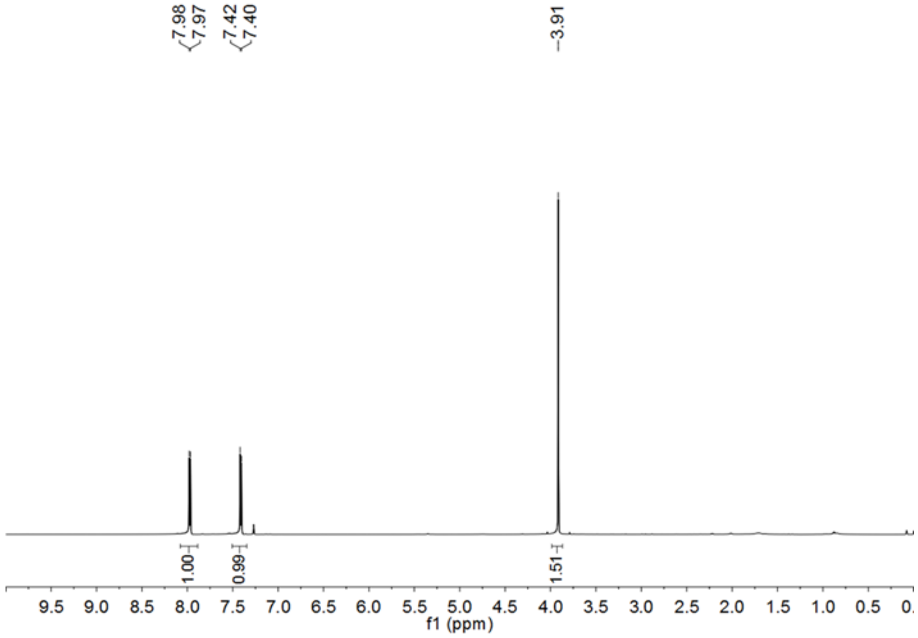
7.98
7.97
7.42
7.40
3.91
1.00
0.99
1.51
f1 (ppm)

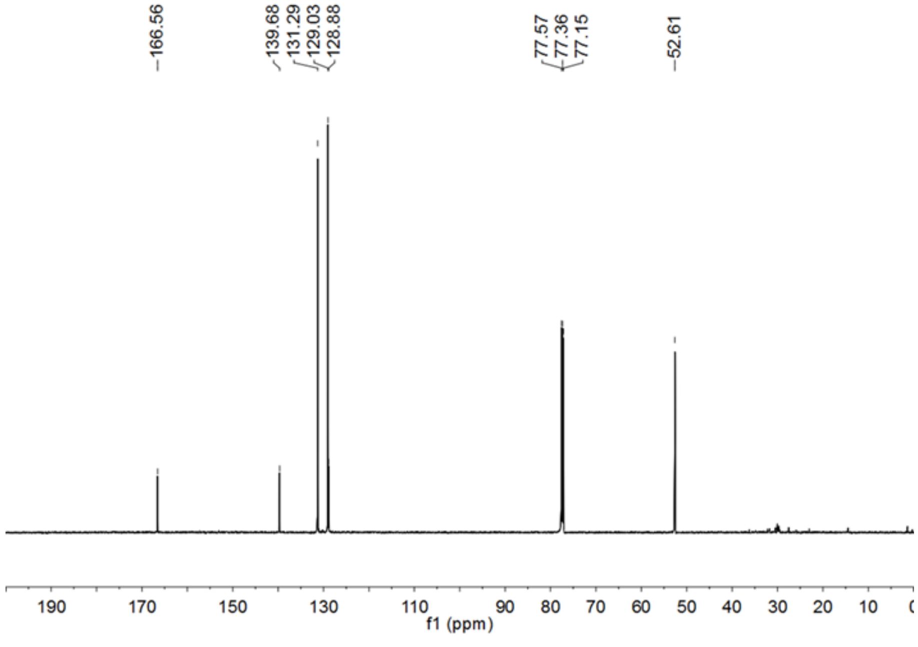
166.56
139.68
131.29
129.03
128.88
77.57
77.36
77.15
52.61
f1 (ppm)

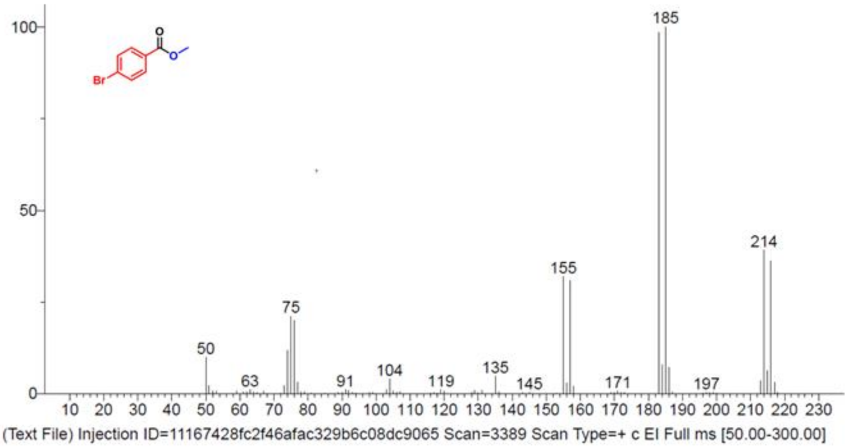
Br
O
O
100
50
0
185
214
155
75
50
63
91
104
119
135
145
171
197
10 20 30 40 50 60 70 80 90 100 110 120 130 140 150 160 170 180 190 200 210 220 230
(Text File) Injection ID=11167428fc2f46afac329b6c08dc9065 Scan=3389 Scan Type=+ c EI Full ms [50.00-300.00]

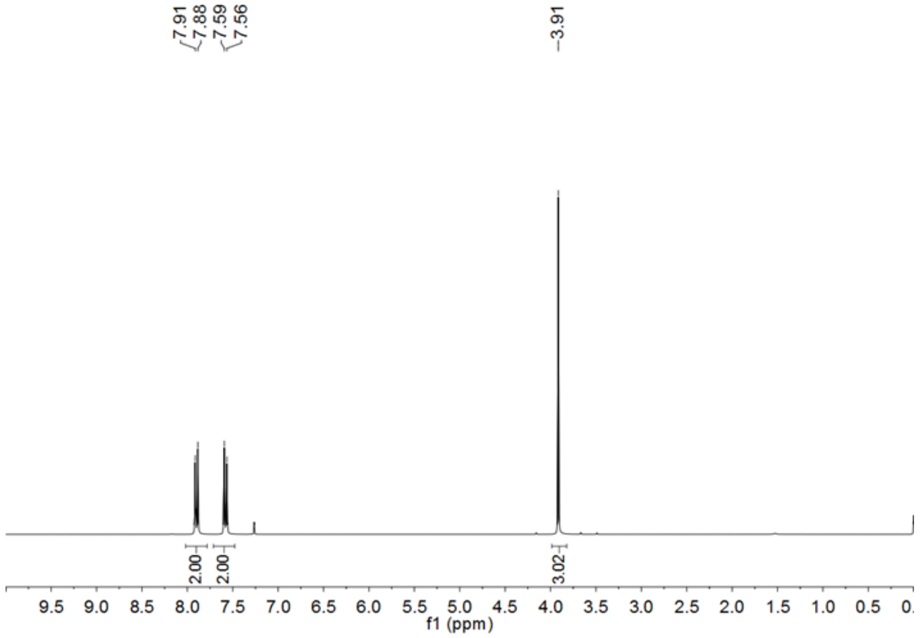
7.91
7.88
7.59
7.56
3.91
2.00
2.00
3.02
9.5 9.0 8.5 8.0 7.5 7.0 6.5 6.0 5.5 5.0 4.5 4.0 3.5 3.0 2.5 2.0 1.5 1.0 0.5 0.
f1 (ppm)

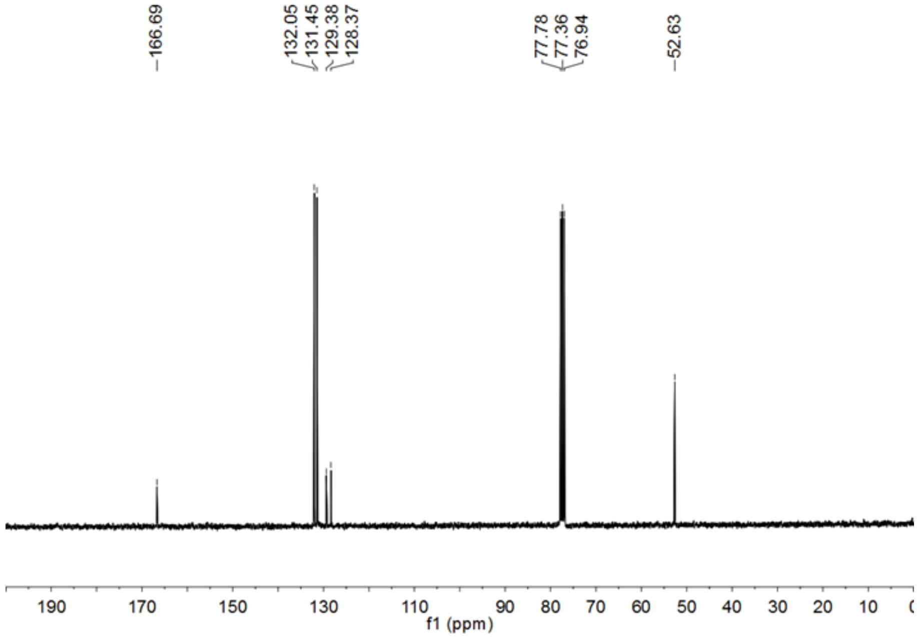
166.69
132.05
131.45
129.38
128.37
77.78
77.36
76.94
52.63
190 170 150 130 110 90 80 70 60 50 40 30 20 10 0
f1 (ppm)

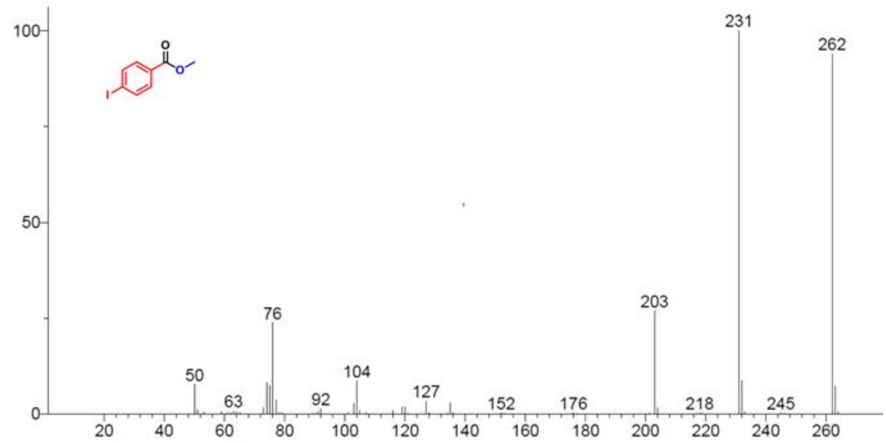

(Text File) Injection ID=99b978bb004a4c39a53059cbf3d3ffa3 Scan=3837 Scan Type=+ c EI Full ms [50.00-300.00]

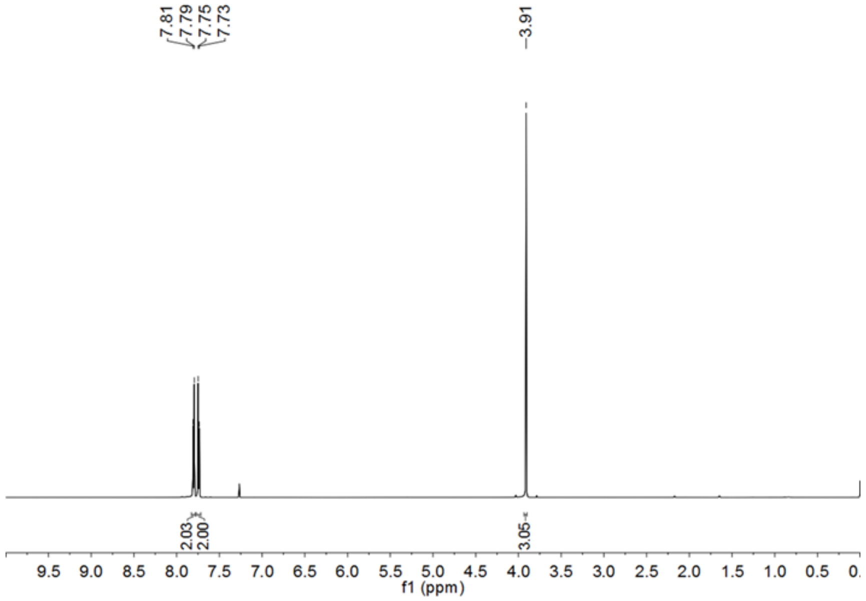

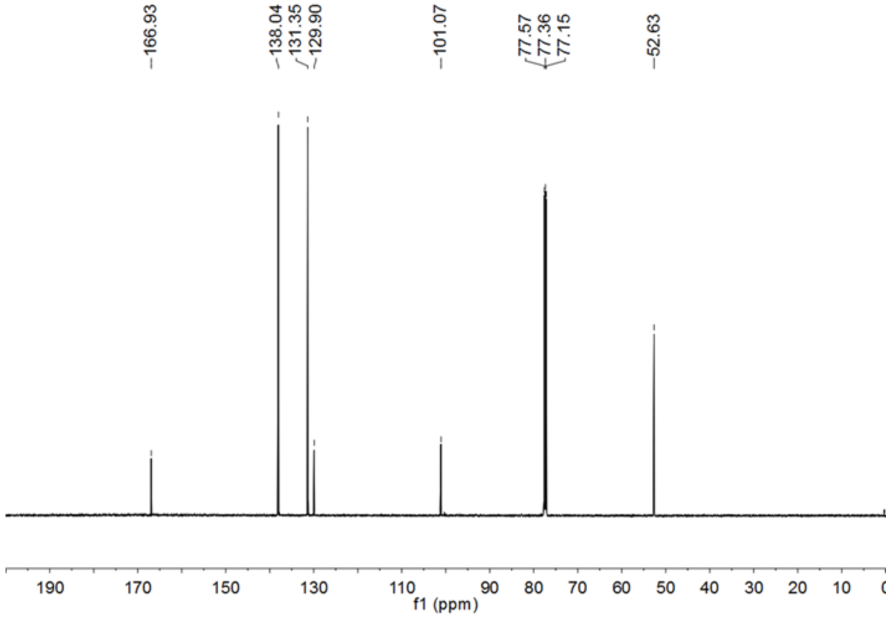

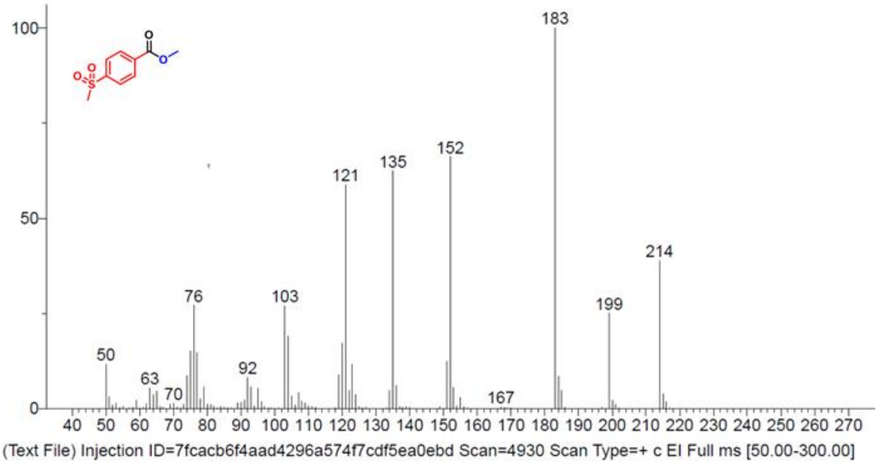

100
50
0
183
152
135
121
214
199
103
76
92
50
63
70
167
40 50 60 70 80 90 100 110 120 130 140 150 160 170 180 190 200 210 220 230 240 250 260 270
(Text File) Injection ID=7fcacb6f4aad4296a574f7cdf5ea0ebd Scan=4930 Scan Type=+ c EI Full ms [50.00-300.00]

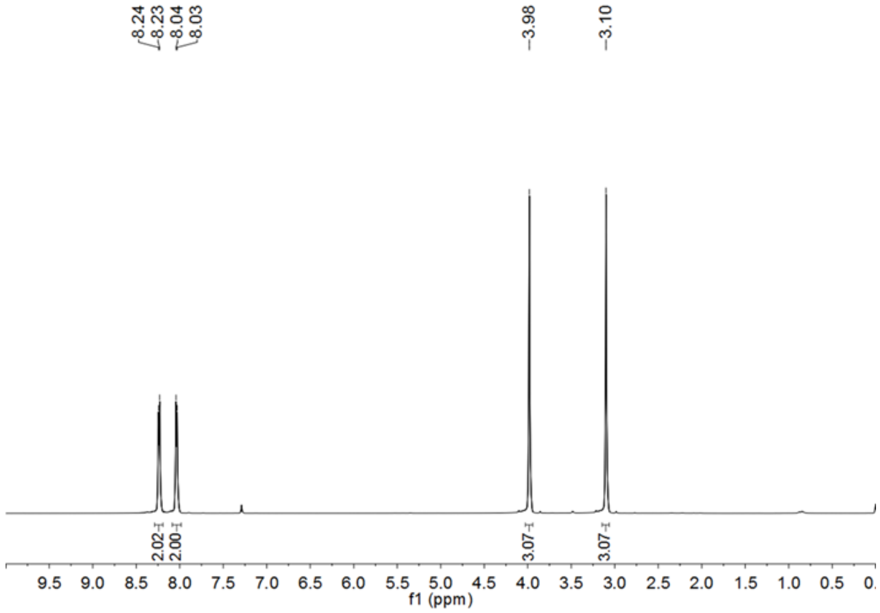

8.24
8.23
8.04
8.03
3.98
3.10
2.02
2.00
3.07
3.07
9.5 9.0 8.5 8.0 7.5 7.0 6.5 6.0 5.5 5.0 4.5 4.0 3.5 3.0 2.5 2.0 1.5 1.0 0.5 0.
f1 (ppm)

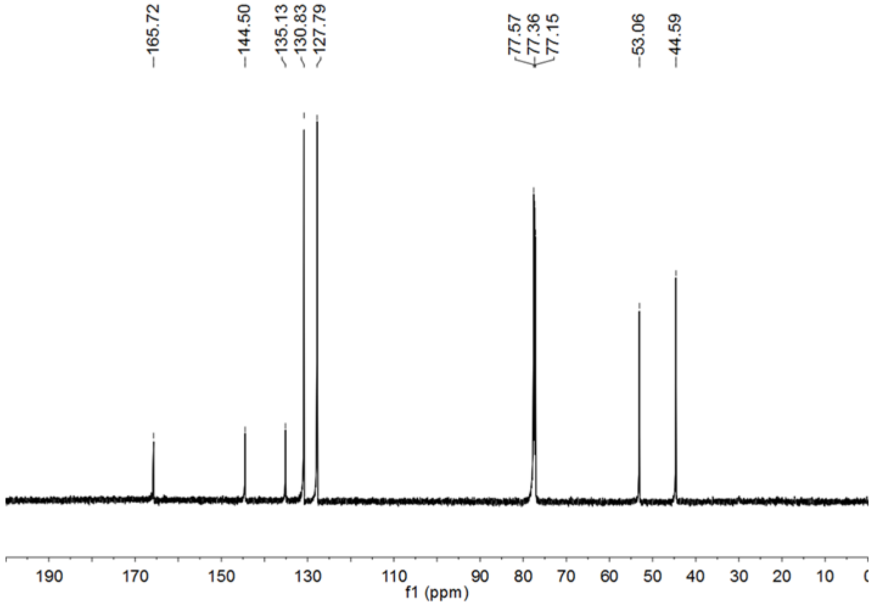

165.72
144.50
135.13
130.83
127.79
77.57
77.36
77.15
53.06
44.59
190 170 150 130 110 90 80 70 60 50 40 30 20 10
f1 (ppm)

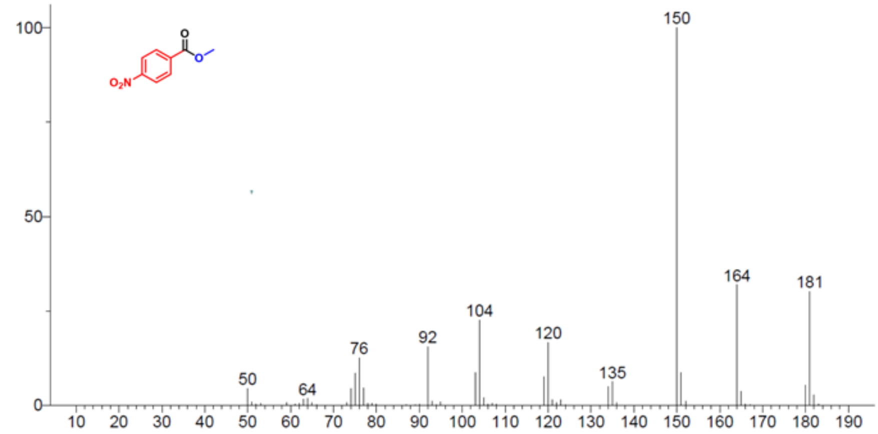

(Text File) Injection ID=3893b34016b7477f853b15829c55f785 Scan=3859 Scan Type=+ c EI Full ms [50.00-300.00]

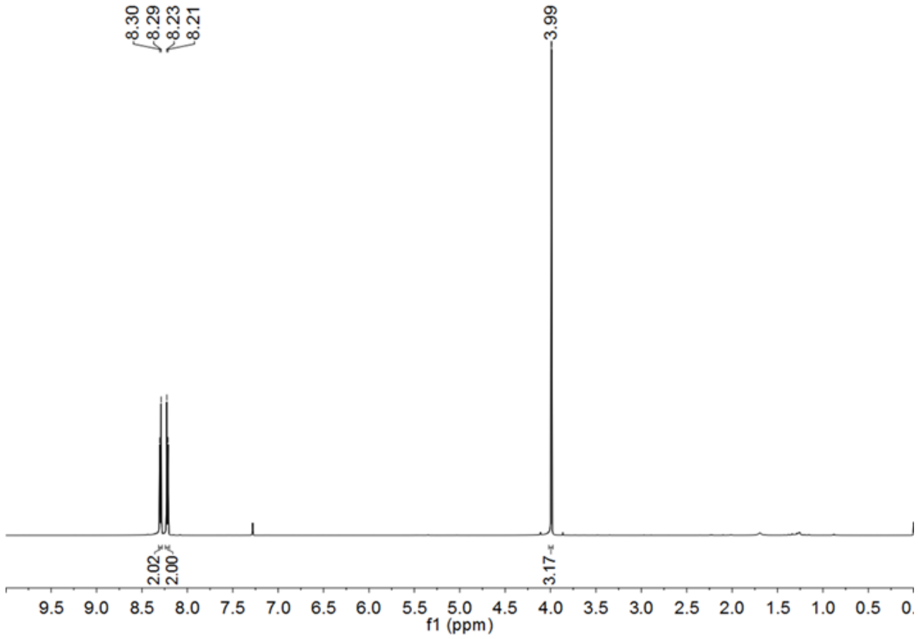

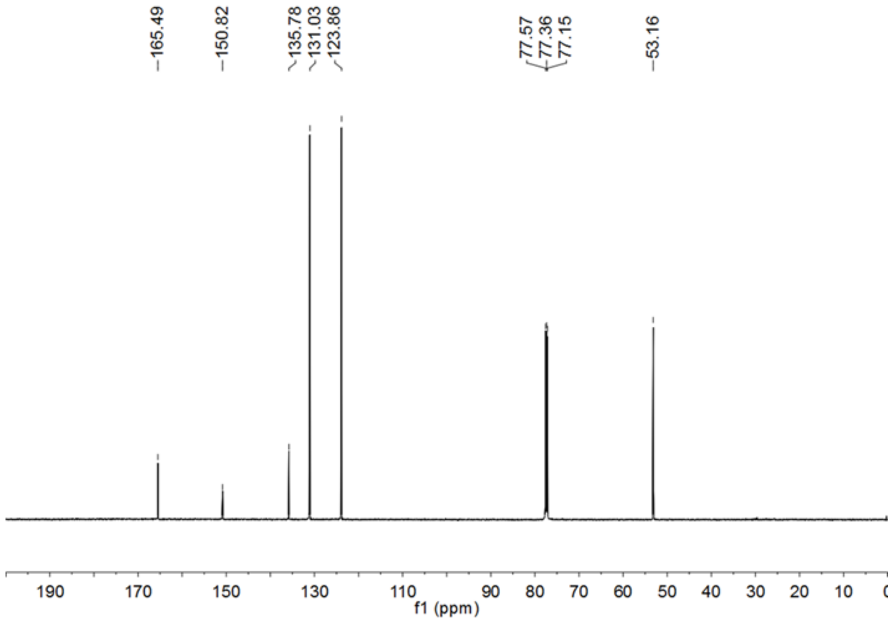

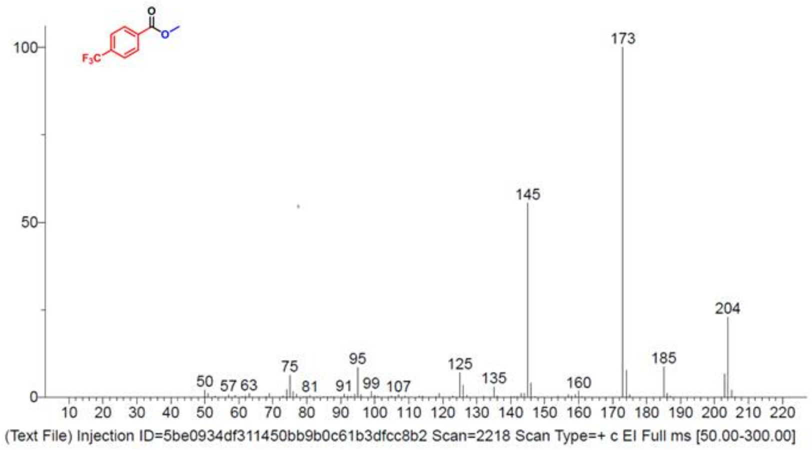
F3C
173
145
204
185
125
135
160
95
75
50 57 63
81
91
99
107
100
50
0
10 20 30 40 50 60 70 80 90 100 110 120 130 140 150 160 170 180 190 200 210 220
(Text File) Injection ID=5be0934df311450bb9b0c61b3dfcc8b2 Scan=2218 Scan Type=+ c EI Full ms [50.00-300.00]

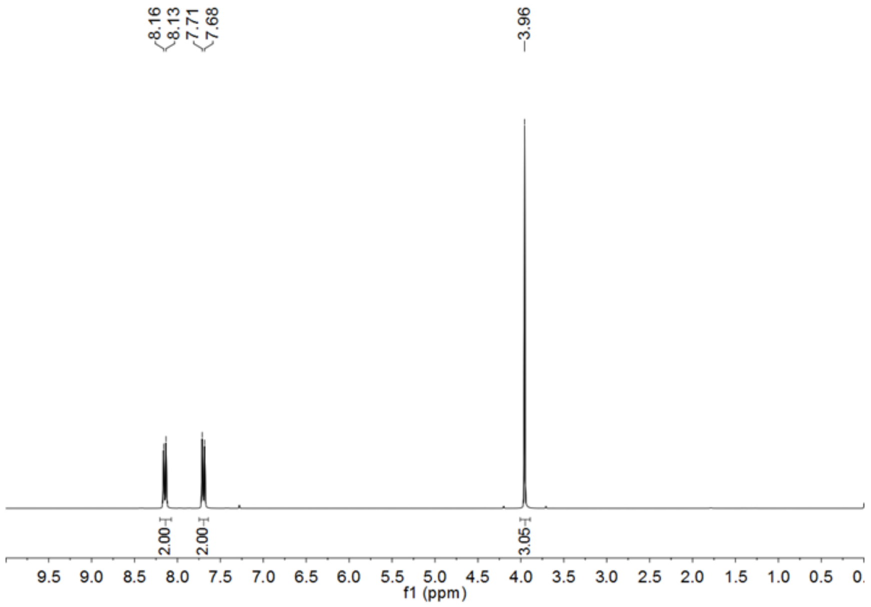
8.16
8.13
7.71
7.68
3.96
2.00
2.00
3.05
9.5 9.0 8.5 8.0 7.5 7.0 6.5 6.0 5.5 5.0 4.5 4.0 3.5 3.0 2.5 2.0 1.5 1.0 0.5 0.
f1 (ppm)

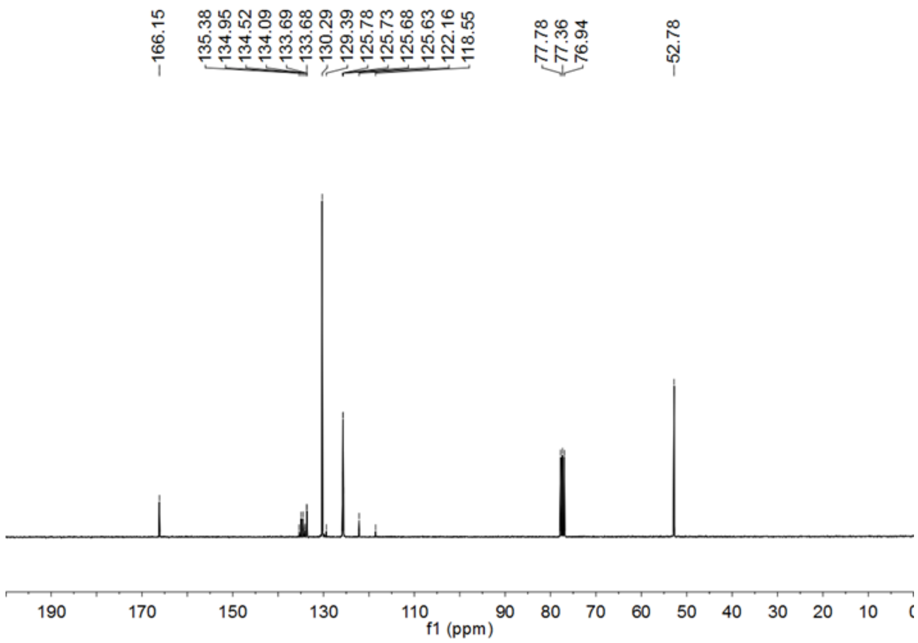
166.15
135.38
134.95
134.52
134.09
133.69
133.68
130.29
129.39
125.78
125.73
125.68
125.63
122.16
118.55
77.78
77.36
76.94
52.78
190 170 150 130 110 90 80 70 60 50 40 30 20 10
f1 (ppm)

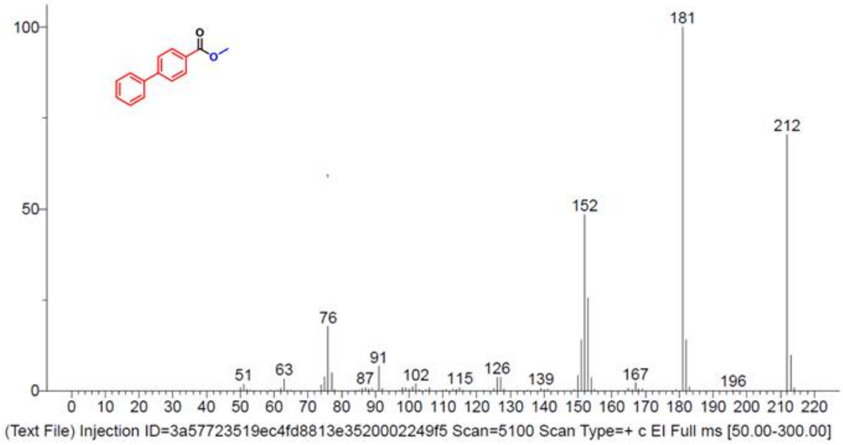
100
50
0
181
212
152
76
91
51
63
87
102
115
126
139
167
196
0 10 20 30 40 50 60 70 80 90 100 110 120 130 140 150 160 170 180 190 200 210 220
(Text File) Injection ID=3a57723519ec4fd8813e3520002249f5 Scan=5100 Scan Type=+ c EI Full ms [50.00-300.00]

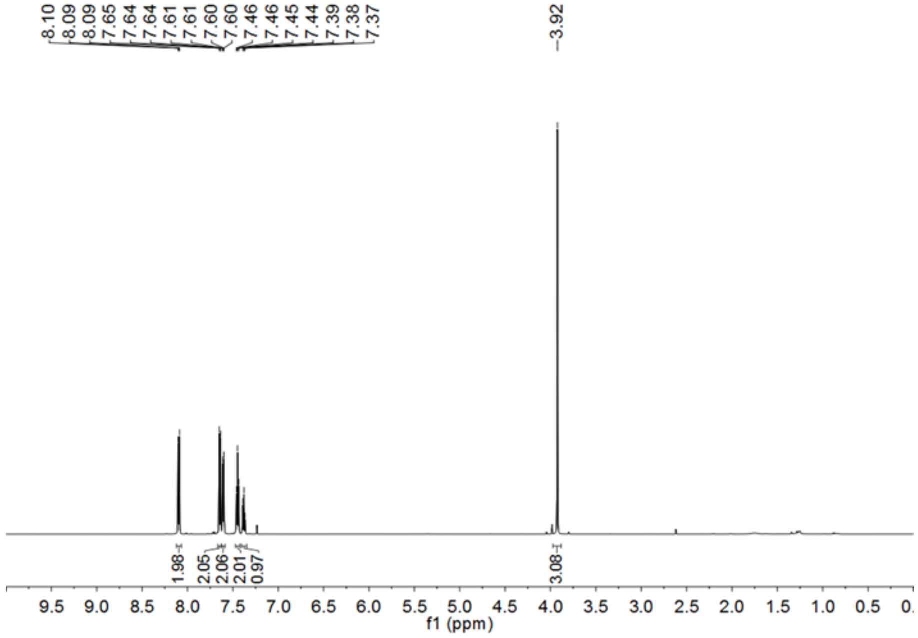
8.10
8.09
8.09
7.65
7.64
7.64
7.61
7.61
7.60
7.60
7.46
7.46
7.45
7.44
7.39
7.38
7.37
3.92
1.98
2.05
2.06
2.01
0.97
3.08
9.5 9.0 8.5 8.0 7.5 7.0 6.5 6.0 5.5 5.0 4.5 4.0 3.5 3.0 2.5 2.0 1.5 1.0 0.5
f1 (ppm)

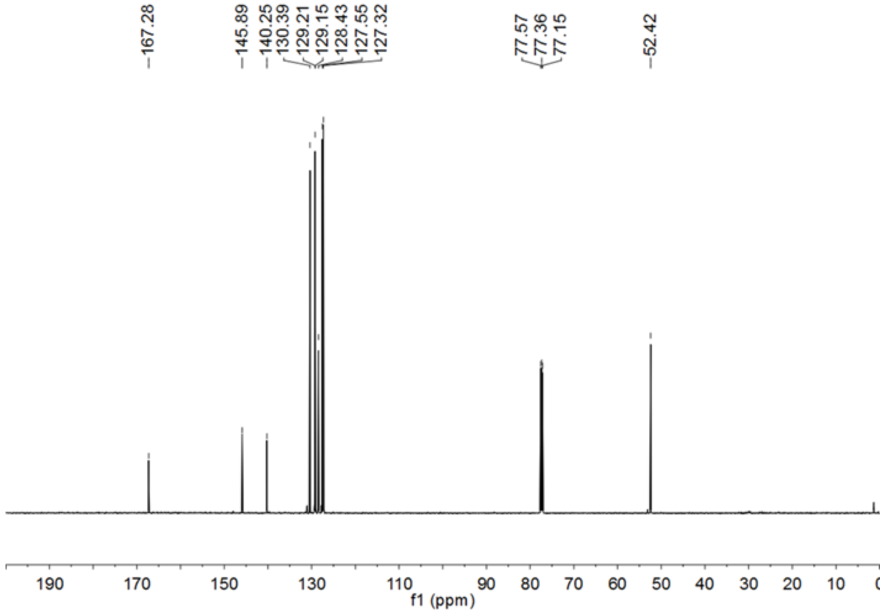
167.28
145.89
140.25
130.39
129.21
129.15
128.43
127.55
127.32
77.57
77.36
77.15
52.42
190 170 150 130 110 90 80 70 60 50 40 30 20 10
f1 (ppm)

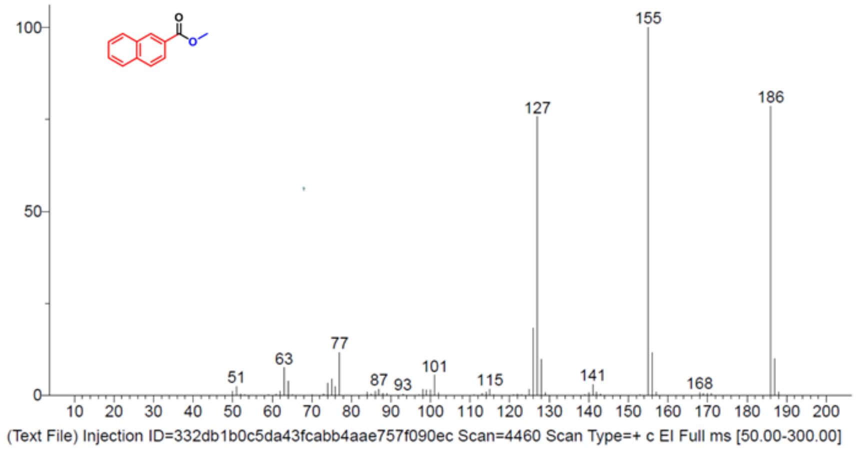
100
50
0
155
127
186
51
63
77
87
93
101
115
141
168
10 20 30 40 50 60 70 80 90 100 110 120 130 140 150 160 170 180 190 200
(Text File) Injection ID=332db1b0c5da43fcabb4aae757f090ec Scan=4460 Scan Type=+ c EI Full ms [50.00-300.00]

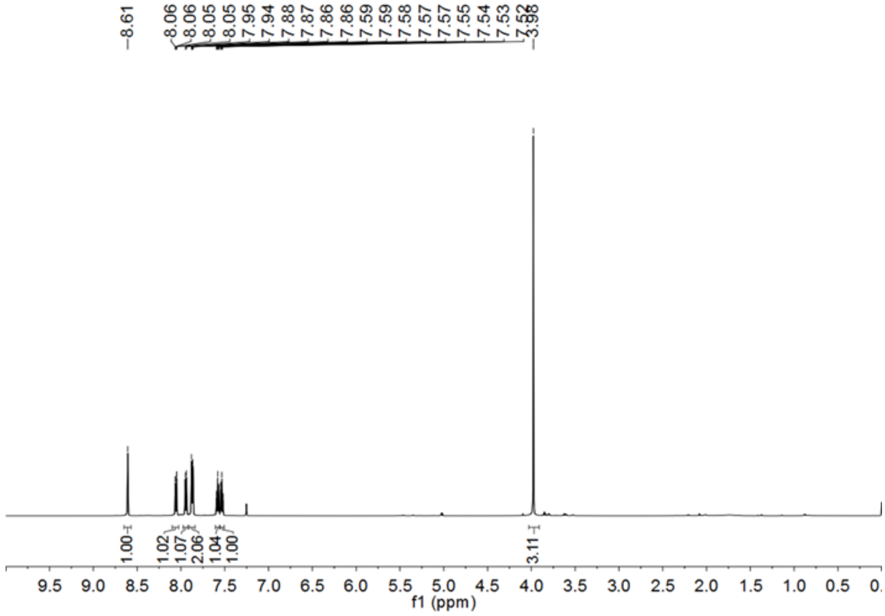
8.61
8.06 8.06 8.05 8.05 7.95 7.94 7.88 7.87 7.86 7.86 7.59 7.59 7.58 7.57 7.57 7.55 7.54 7.53 7.52
3.98
1.00
1.02
1.07
2.06
1.04
1.00
3.11
9.5 9.0 8.5 8.0 7.5 7.0 6.5 6.0 5.5 5.0 4.5 4.0 3.5 3.0 2.5 2.0 1.5 1.0 0.5 0.
f1 (ppm)

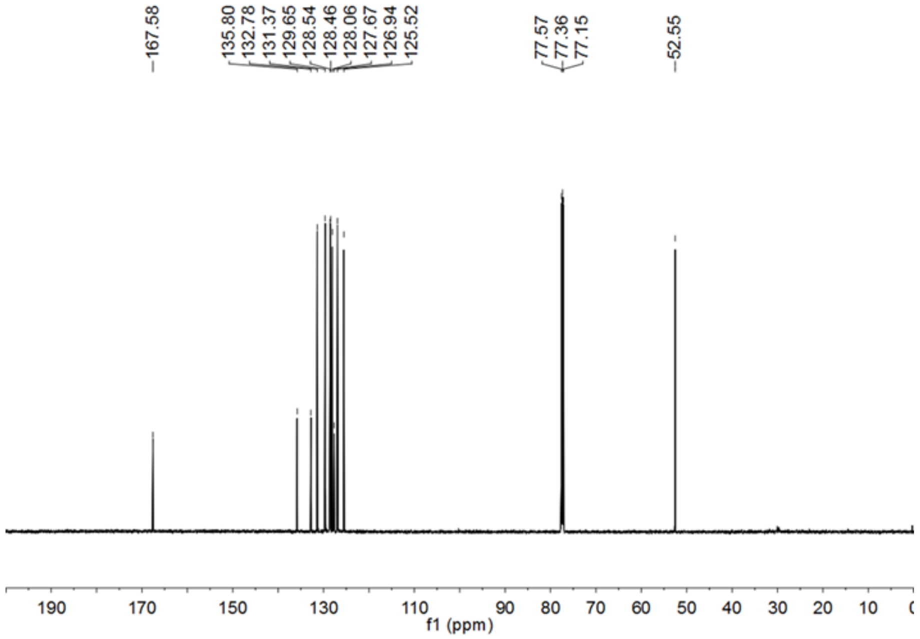
167.58
135.80 132.78 131.37 129.65 128.54 128.46 128.06 127.67 126.94 125.52
77.57 77.36 77.15
52.55
190 170 150 130 110 90 80 70 60 50 40 30 20 10
f1 (ppm)

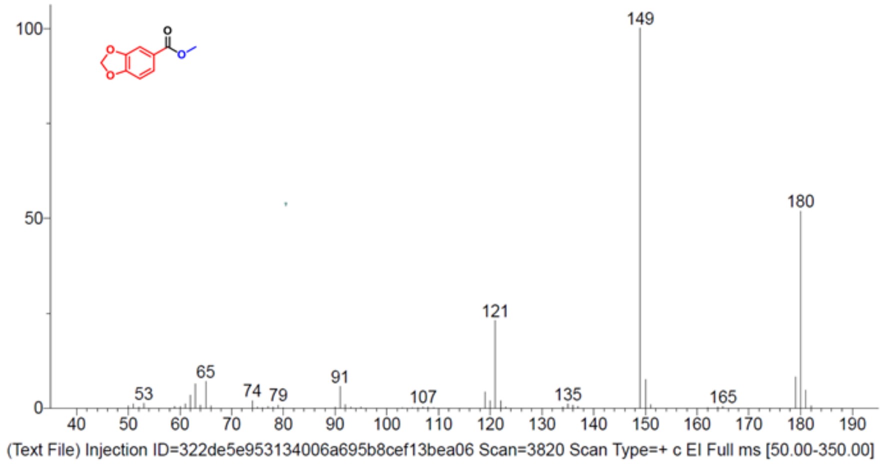
100
50
0
149
180
121
65
53
74 79
91
107
135
165
40 50 60 70 80 90 100 110 120 130 140 150 160 170 180 190
(Text File) Injection ID=322de5e953134006a695b8cef13bea06 Scan=3820 Scan Type=+ c EI Full ms [50.00-350.00]

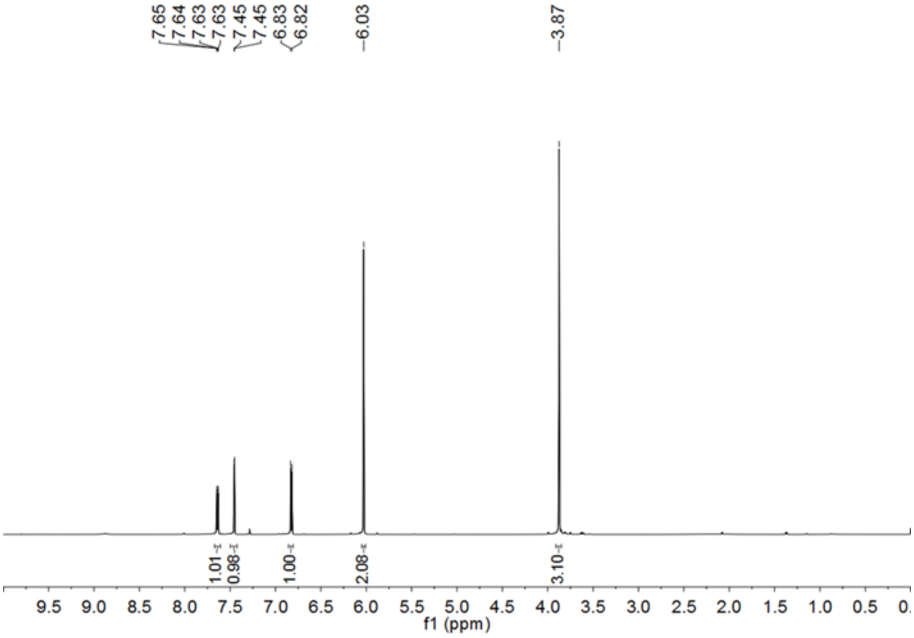
7.65
7.64
7.63
7.63
7.45
7.45
6.83
6.82
6.03
3.87
1.01
0.98
1.00
2.08
3.10
9.5 9.0 8.5 8.0 7.5 7.0 6.5 6.0 5.5 5.0 4.5 4.0 3.5 3.0 2.5 2.0 1.5 1.0 0.5 0.
f1 (ppm)

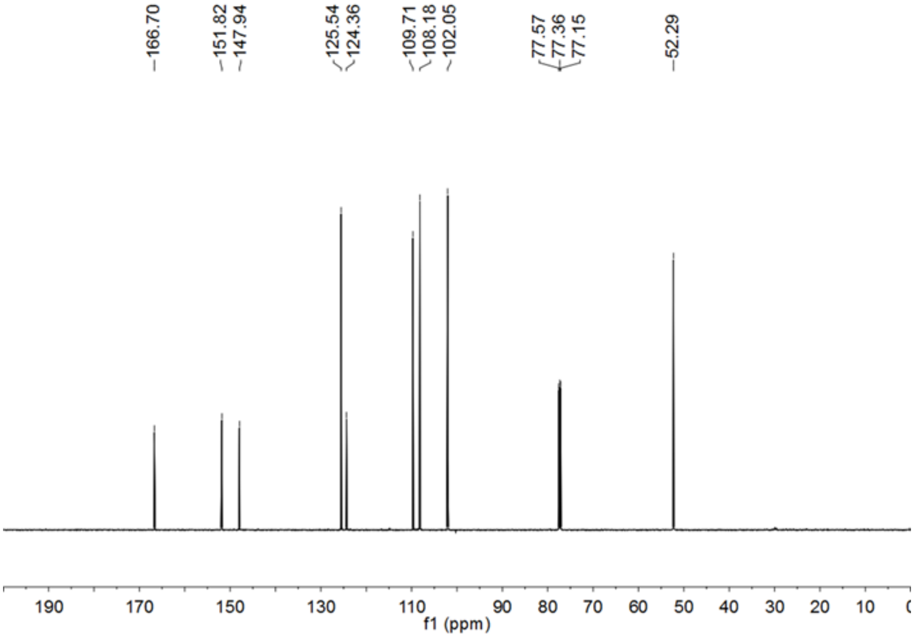
166.70
151.82
147.94
125.54
124.36
109.71
108.18
102.05
77.57
77.36
77.15
52.29
190 170 150 130 110 90 80 70 60 50 40 30 20 10
f1 (ppm)

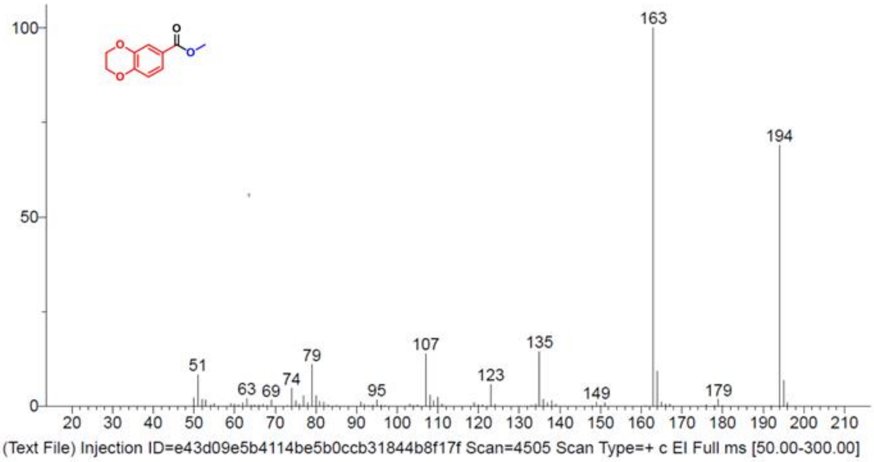
100
50
0
163
194
51
63
69
74
79
95
107
123
135
149
179
20 30 40 50 60 70 80 90 100 110 120 130 140 150 160 170 180 190 200 210
(Text File) Injection ID=e43d09e5b4114be5b0ccb31844b8f17f Scan=4505 Scan Type=+ c EI Full ms [50.00-300.00]

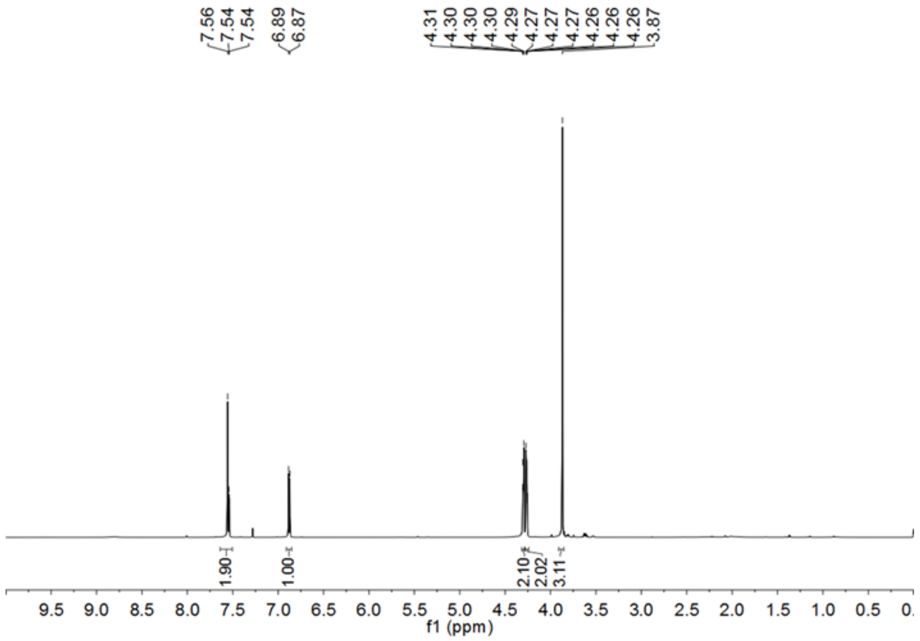
7.56
7.54
7.54
6.89
6.87
4.31
4.30
4.30
4.30
4.29
4.27
4.27
4.27
4.26
4.26
4.26
3.87
1.90
1.00
2.10
2.02
3.11
9.5 9.0 8.5 8.0 7.5 7.0 6.5 6.0 5.5 5.0 4.5 4.0 3.5 3.0 2.5 2.0 1.5 1.0 0.5 0.
f1 (ppm)

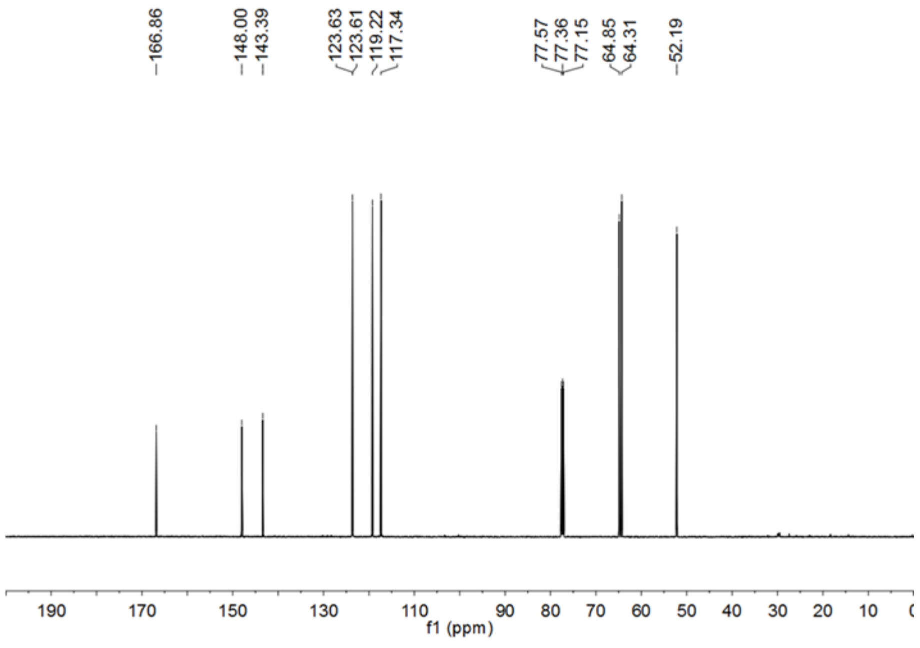
166.86
148.00
143.39
123.63
123.61
119.22
117.34
77.57
77.36
77.15
64.85
64.31
52.19
190 170 150 130 110 90 80 70 60 50 40 30 20 10
f1 (ppm)

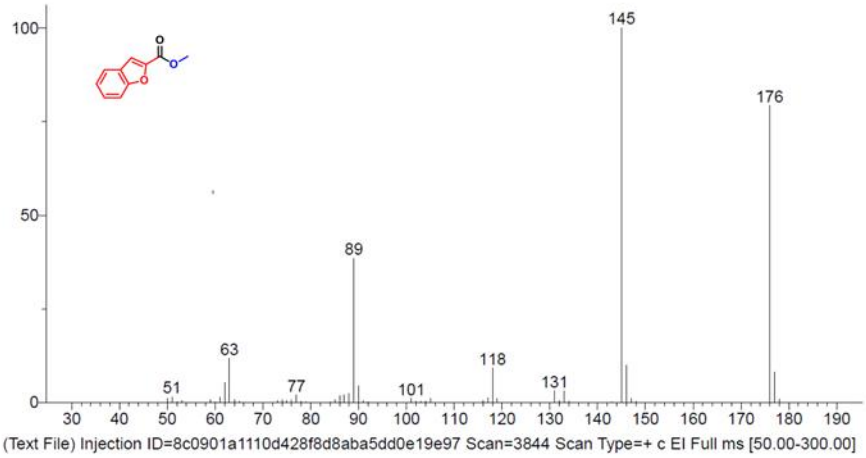
145
176
89
63
118
131
51
77
101
100
50
0
30 40 50 60 70 80 90 100 110 120 130 140 150 160 170 180 190
(Text File) Injection ID=8c0901a1110d428f8d8aba5dd0e19e97 Scan=3844 Scan Type=+ c EI Full ms [50.00-300.00]

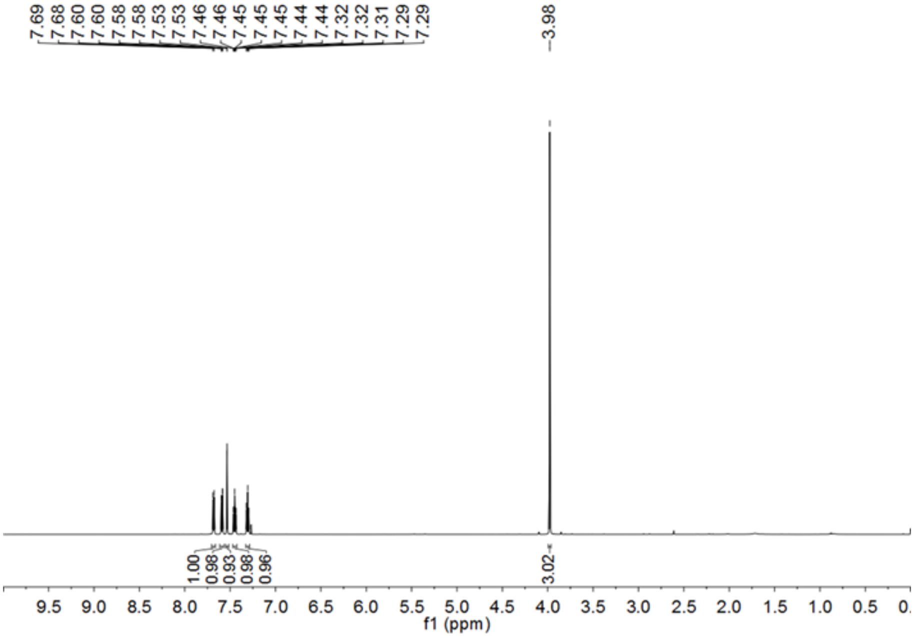
7.69 7.68 7.60 7.60 7.58 7.58 7.53 7.53 7.46 7.45 7.45 7.45 7.44 7.44 7.32 7.32 7.31 7.29 7.29
3.98
1.00 0.98 0.93 0.98 0.96
3.02
9.5 9.0 8.5 8.0 7.5 7.0 6.5 6.0 5.5 5.0 4.5 4.0 3.5 3.0 2.5 2.0 1.5 1.0 0.5 0.
f1 (ppm)

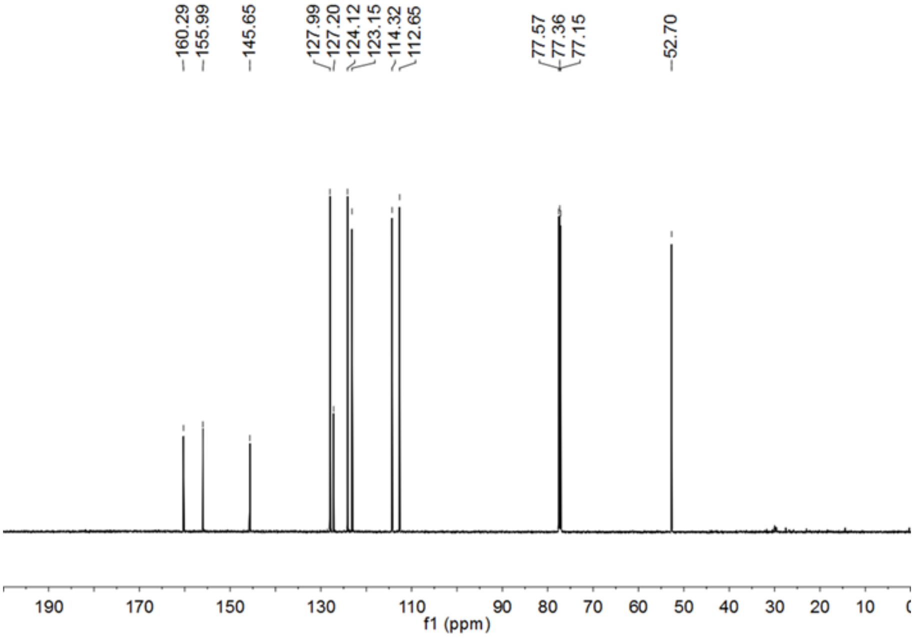
160.29 155.99 145.65 127.99 127.20 124.12 123.15 114.32 112.65 77.57 77.36 77.15 52.70
190 170 150 130 110 90 80 70 60 50 40 30 20 10
f1 (ppm)

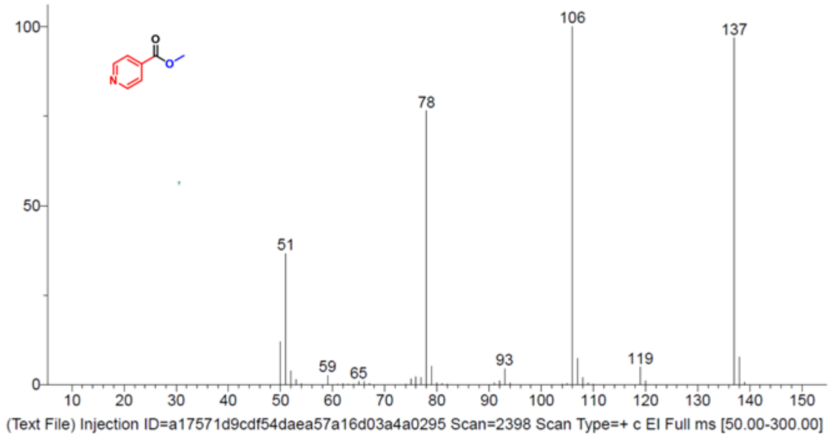
106
137
78
51
59
65
93
119
100
50
0
10 20 30 40 50 60 70 80 90 100 110 120 130 140 150
(Text File) Injection ID=a17571d9cdf54daea57a16d03a4a0295 Scan=2398 Scan Type=+ c EI Full ms [50.00-300.00]

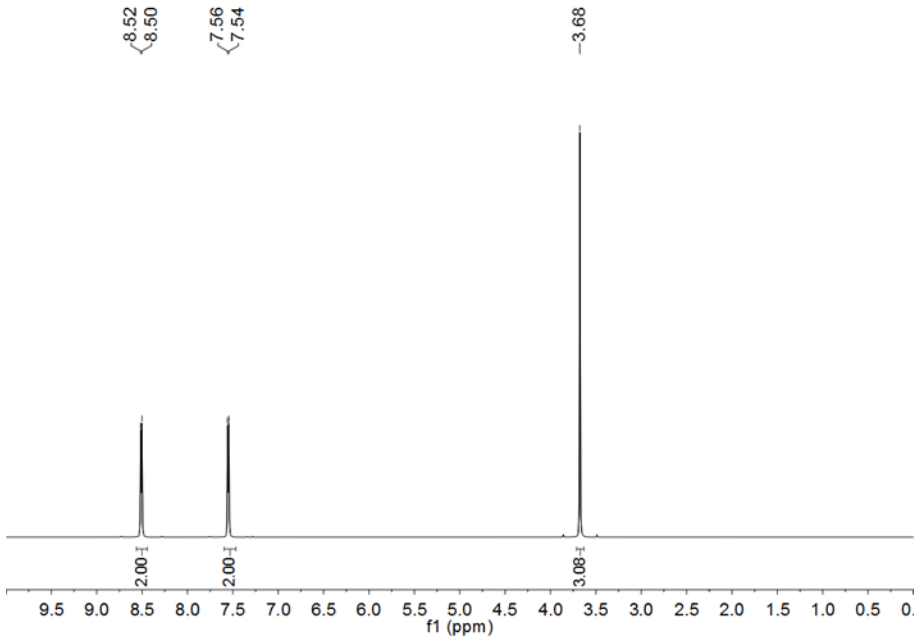
8.52
8.50
7.56
7.54
3.68
2.00
2.00
3.08
9.5 9.0 8.5 8.0 7.5 7.0 6.5 6.0 5.5 5.0 4.5 4.0 3.5 3.0 2.5 2.0 1.5 1.0 0.5
f1 (ppm)

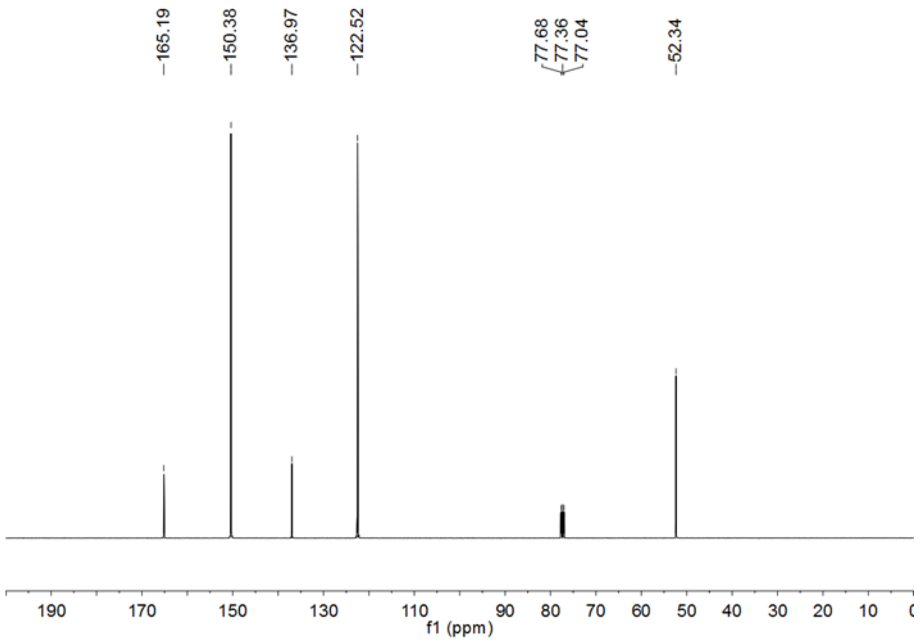
165.19
150.38
136.97
122.52
77.68
77.36
77.04
52.34
190 170 150 130 110 90 80 70 60 50 40 30 20 10
f1 (ppm)

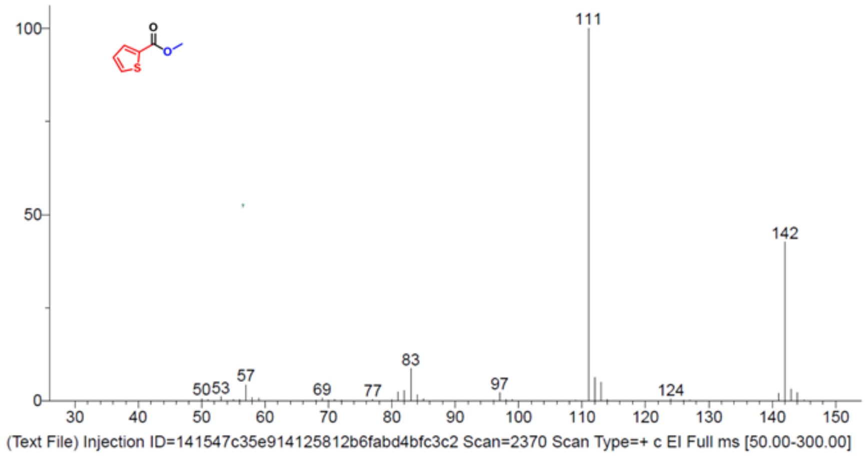
111
142
50 53
57
69
77
83
97
124
(Text File) Injection ID=141547c35e914125812b6fabd4bfc3c2 Scan=2370 Scan Type=+ c EI Full ms [50.00-300.00]

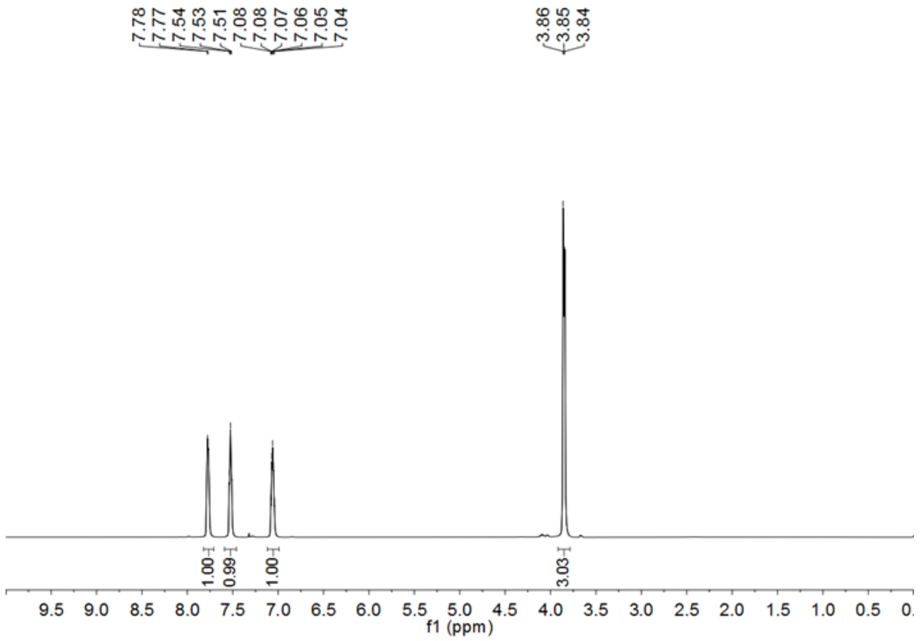
7.78
7.77
7.54
7.53
7.51
7.08
7.08
7.07
7.06
7.05
7.04
3.86
3.85
3.84
1.00
0.99
1.00
3.03
f1 (ppm)

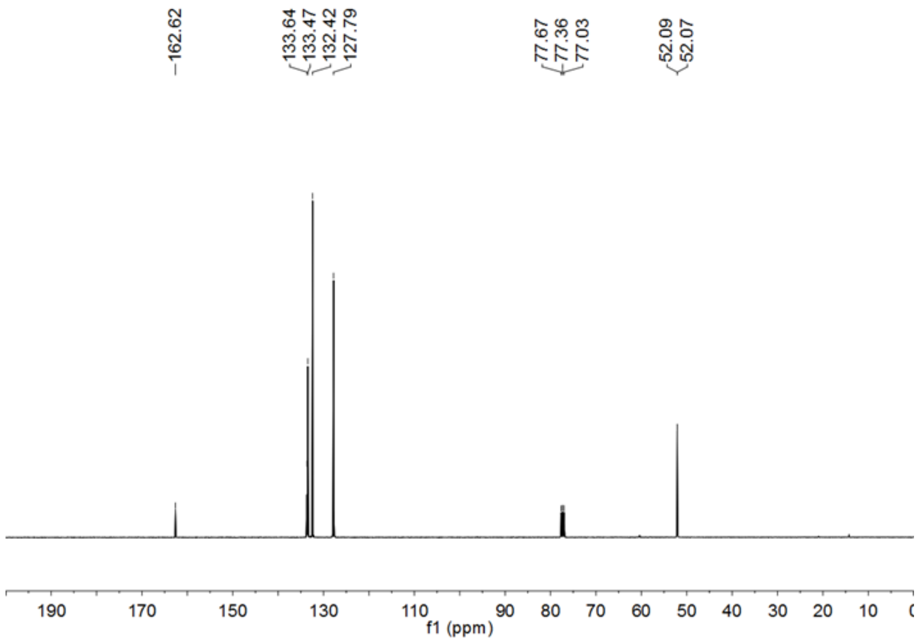
162.62
133.64
133.47
132.42
127.79
77.67
77.36
77.03
52.09
52.07
f1 (ppm)

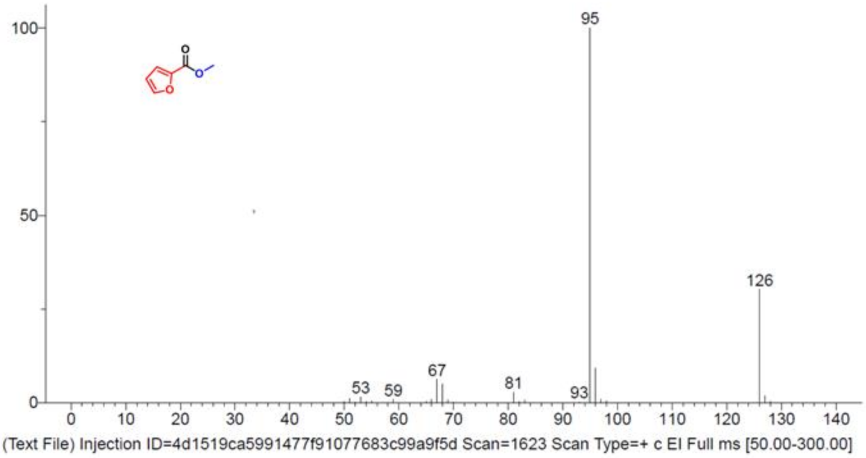

100
50
0
95
126
67
81
53
59
93
0
10
20
30
40
50
60
70
80
90
100
110
120
130
140
(Text File) Injection ID=4d1519ca5991477f91077683c99a9f5d Scan=1623 Scan Type=+ c EI Full ms [50.00-300.00]

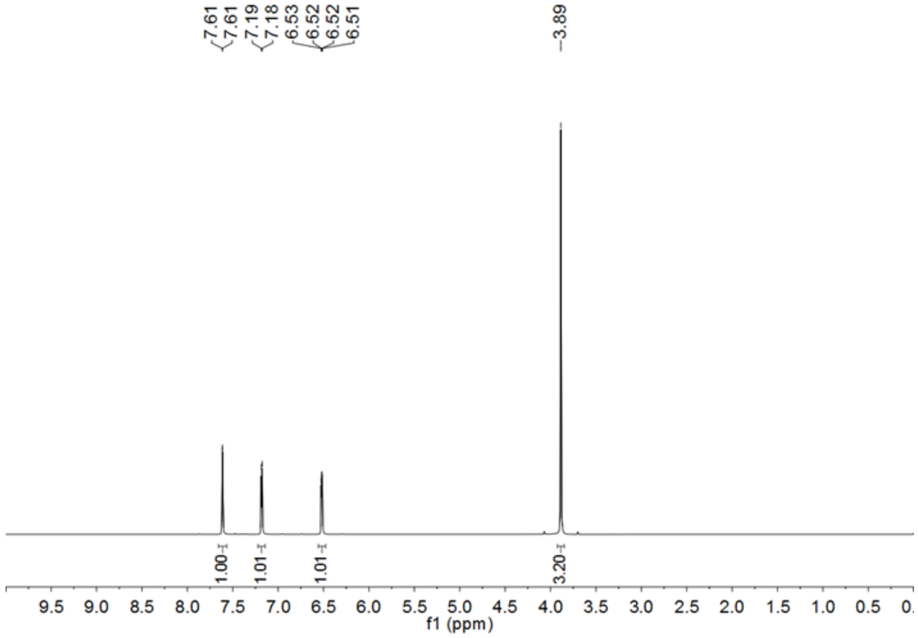

7.61
7.61
7.19
7.18
6.53
6.52
6.52
6.51
3.89
1.00
1.01
1.01
3.20
9.5
9.0
8.5
8.0
7.5
7.0
6.5
6.0
5.5
5.0
4.5
4.0
3.5
3.0
2.5
2.0
1.5
1.0
0.5
f1 (ppm)

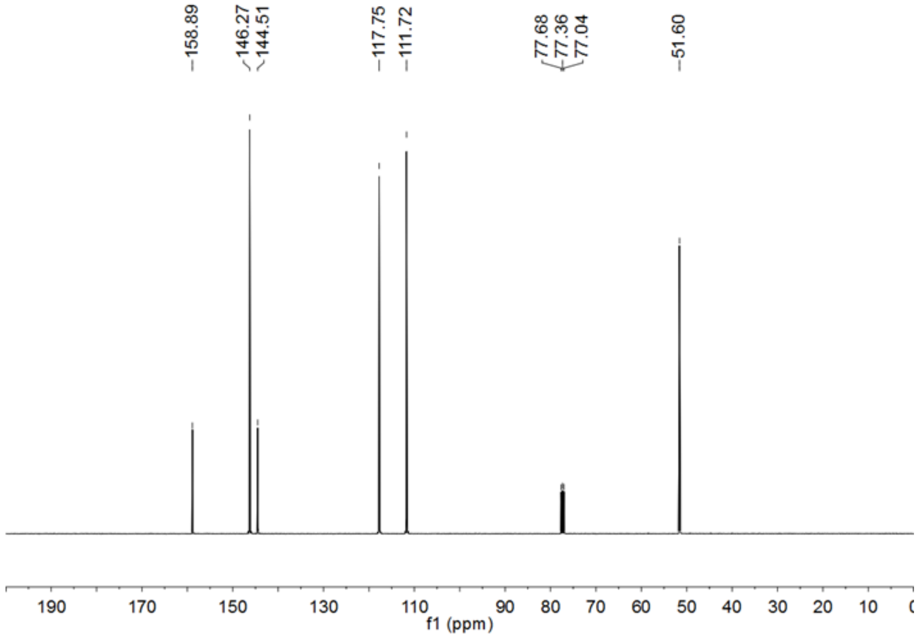

158.89
146.27
144.51
117.75
111.72
77.68
77.36
77.04
51.60
190
170
150
130
110
90
80
70
60
50
40
30
20
10
f1 (ppm)

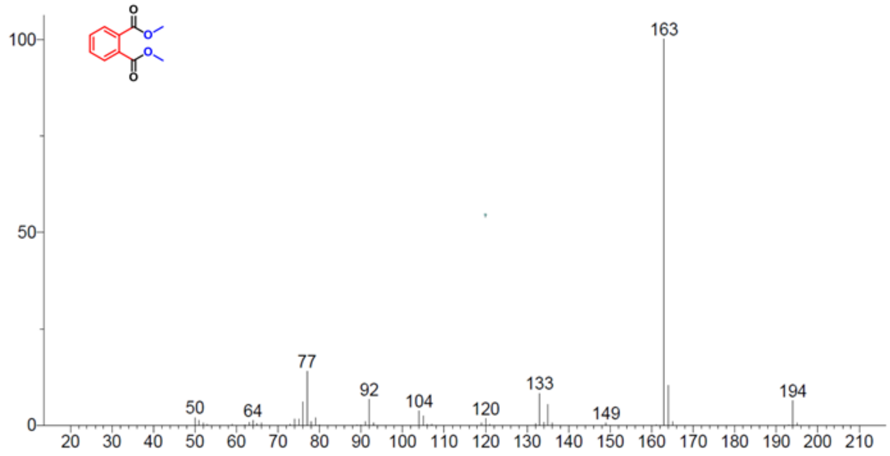
100
50
0
163
77
92
104
120
133
149
194
50
64
20 30 40 50 60 70 80 90 100 110 120 130 140 150 160 170 180 190 200 210

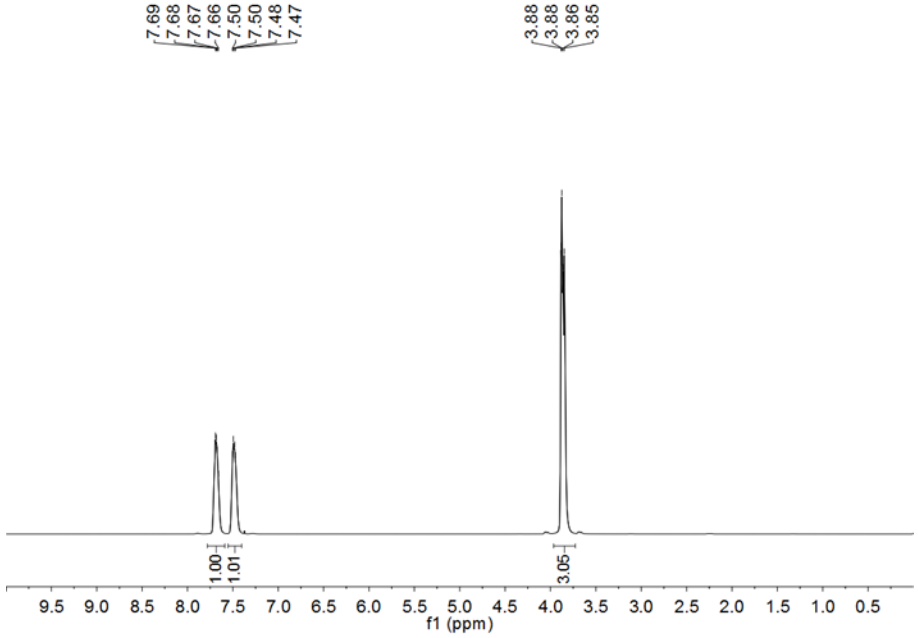
7.69
7.68
7.67
7.66
7.50
7.50
7.48
7.47
3.88
3.88
3.86
3.85
1.00
1.01
3.05
9.5 9.0 8.5 8.0 7.5 7.0 6.5 6.0 5.5 5.0 4.5 4.0 3.5 3.0 2.5 2.0 1.5 1.0 0.5
f1 (ppm)

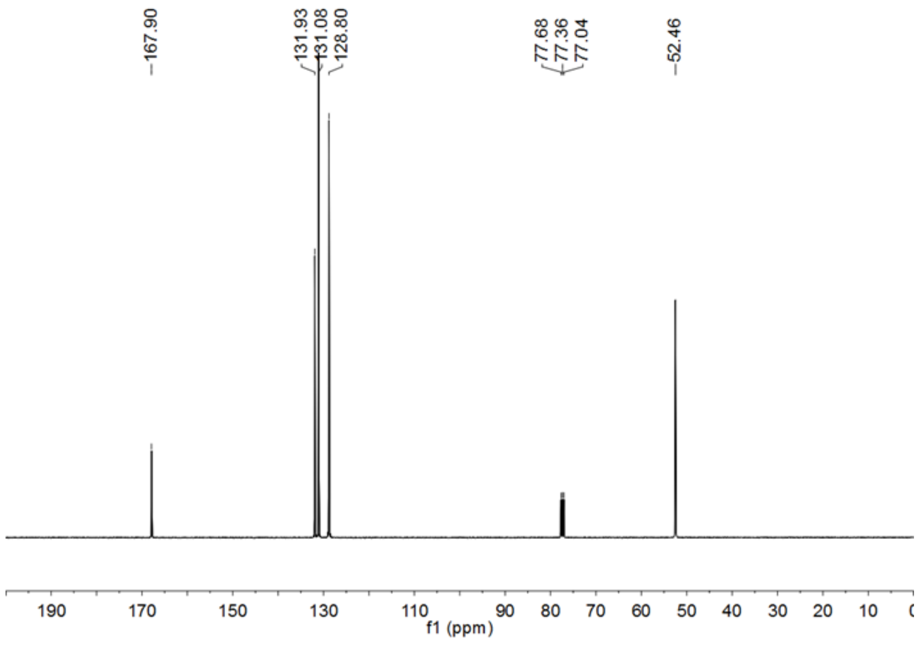
167.90
131.93
131.08
128.80
77.68
77.36
77.04
52.46
190 170 150 130 110 90 80 70 60 50 40 30 20 10
f1 (ppm)

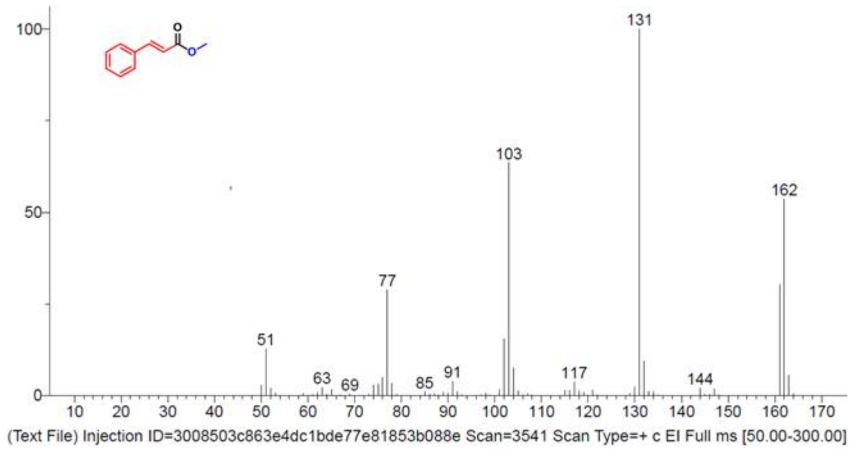

100
50
0
131
103
162
77
51
63
69
85
91
117
144
10 20 30 40 50 60 70 80 90 100 110 120 130 140 150 160 170
(Text File) Injection ID=3008503c863e4dc1bde77e81853b088e Scan=3541 Scan Type=+ c EI Full ms [50.00-300.00]

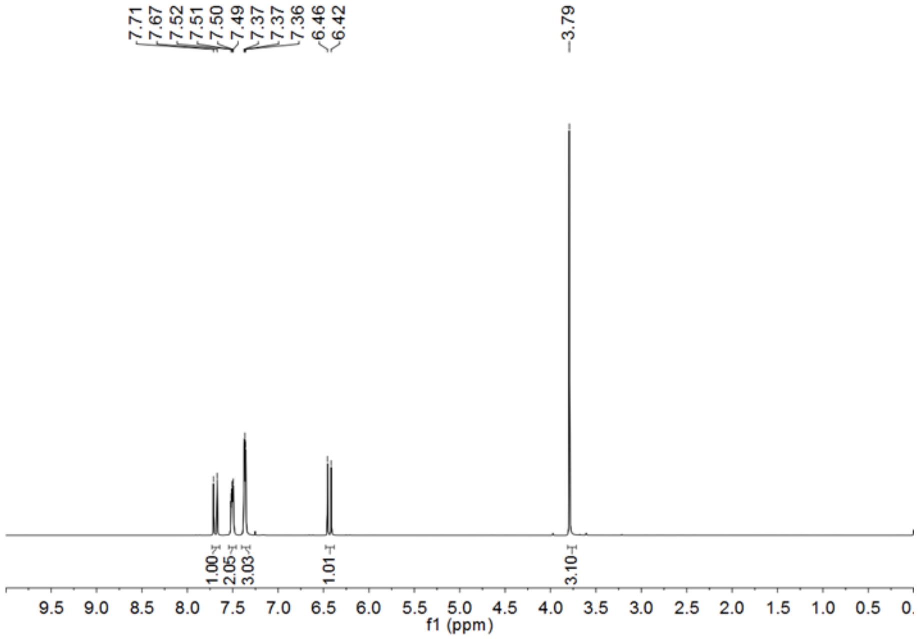

7.71
7.67
7.52
7.51
7.50
7.49
7.37
7.37
7.36
6.46
6.42
3.79
1.00
2.05
3.03
1.01
3.10
9.5 9.0 8.5 8.0 7.5 7.0 6.5 6.0 5.5 5.0 4.5 4.0 3.5 3.0 2.5 2.0 1.5 1.0 0.5 0.
f1 (ppm)

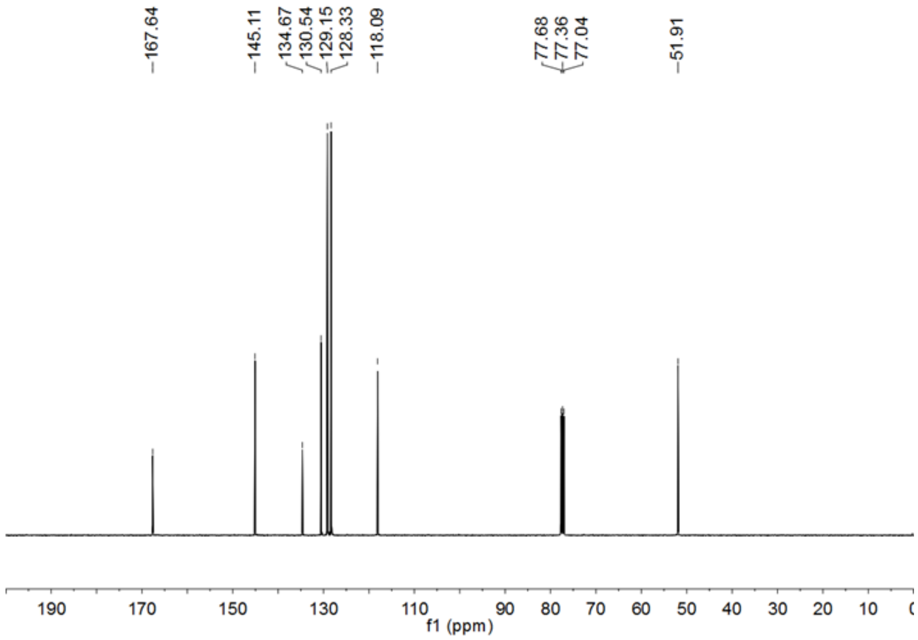

167.64
145.11
134.67
130.54
129.15
128.33
118.09
77.68
77.36
77.04
51.91
190 170 150 130 110 90 80 70 60 50 40 30 20 10
f1 (ppm)

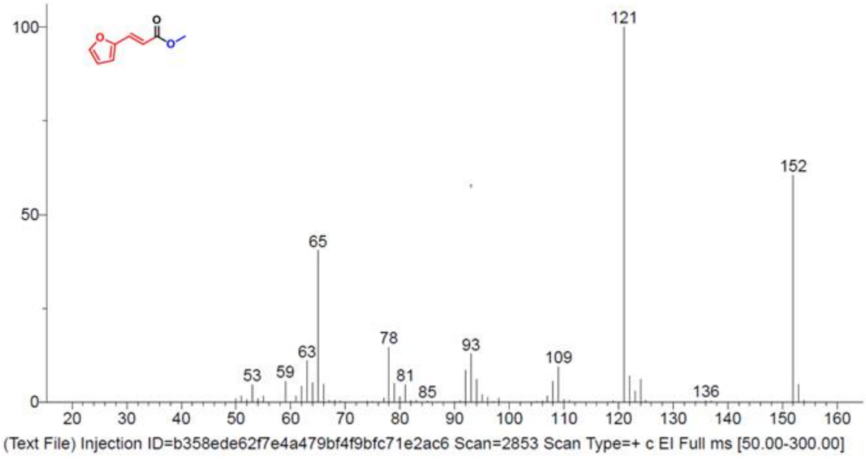
121
152
65
78
93
63
109
59
53
81
85
136
(Text File) Injection ID=b358ede62f7e4a479bf4f9bfc71e2ac6 Scan=2853 Scan Type=+ c EI Full ms [50.00-300.00]

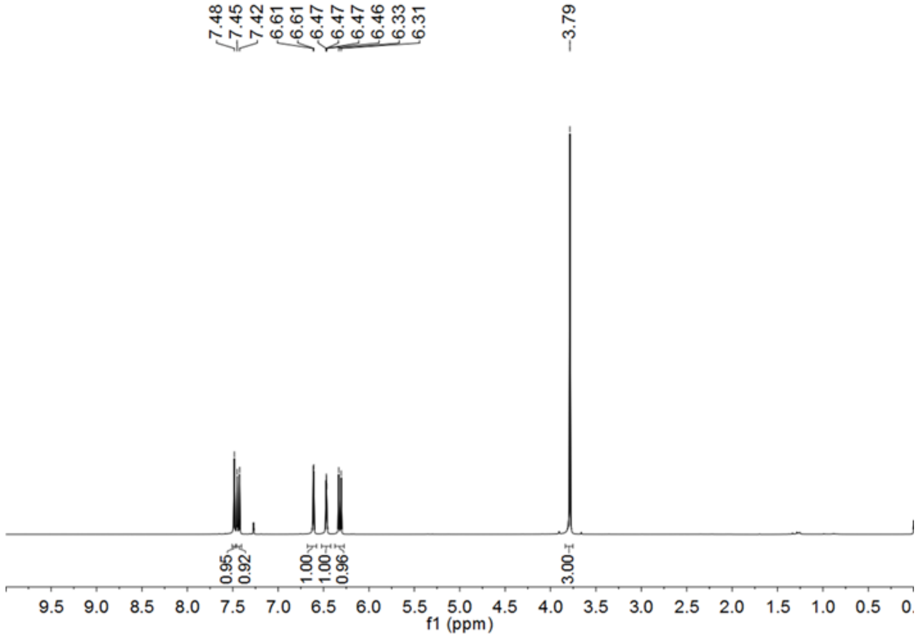
7.48
7.45
7.42
6.61
6.61
6.47
6.47
6.46
6.33
6.31
3.79
0.95
0.92
1.00
1.00
0.96
3.00
f1 (ppm)

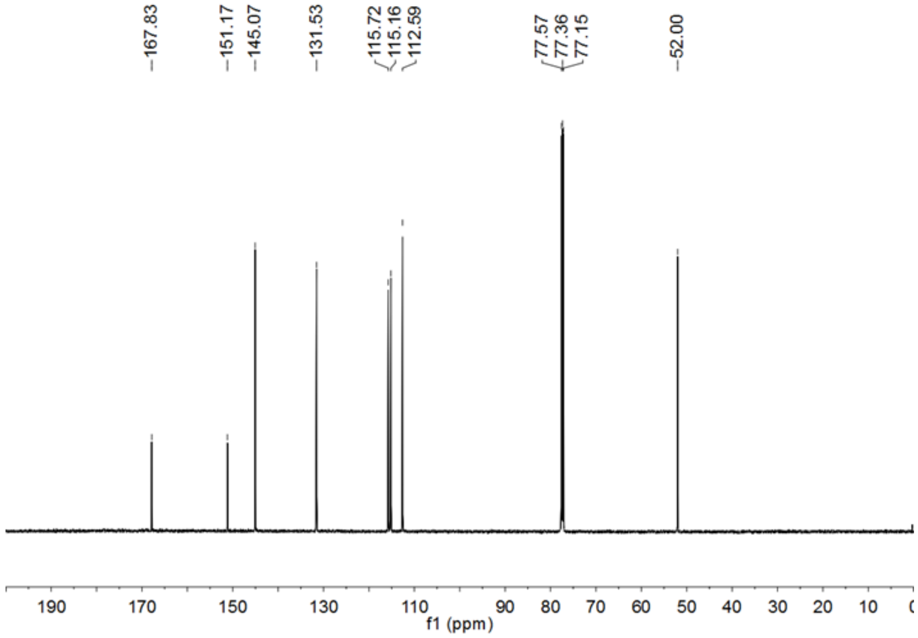
167.83
151.17
145.07
131.53
115.72
115.16
112.59
77.57
77.36
77.15
52.00
f1 (ppm)

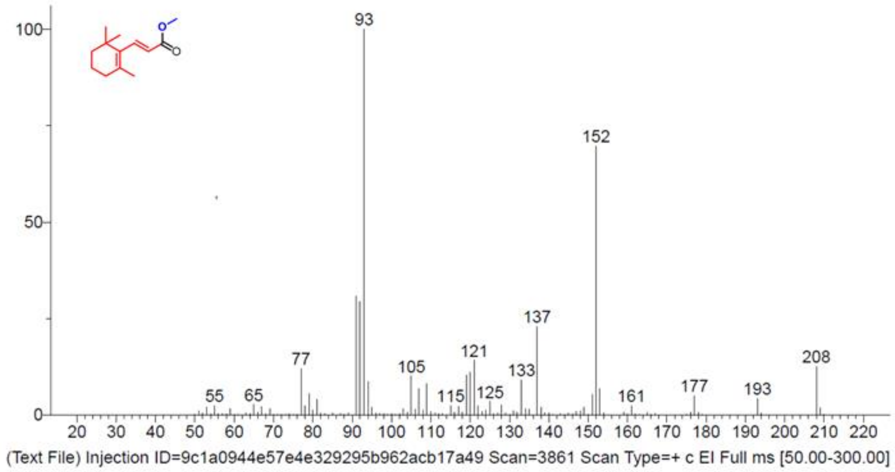
93
152
137
121
77
105
133
208
177
193
55
65
115
125
161
(Text File) Injection ID=9c1a0944e57e4e329295b962acb17a49 Scan=3861 Scan Type=+ c EI Full ms [50.00-300.00]

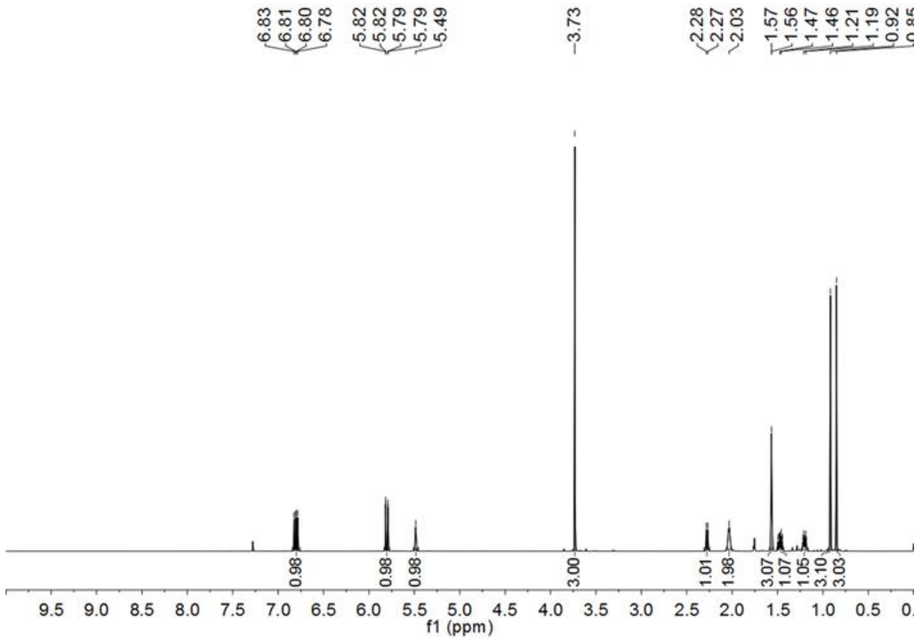
6.83
6.81
6.80
6.78
5.82
5.82
5.79
5.79
5.49
3.73
2.28
2.27
2.03
1.57
1.56
1.47
1.46
1.21
1.19
0.92
0.98
0.98
0.98
3.00
1.01
1.98
3.07
1.07
1.05
3.10
3.03
f1 (ppm)

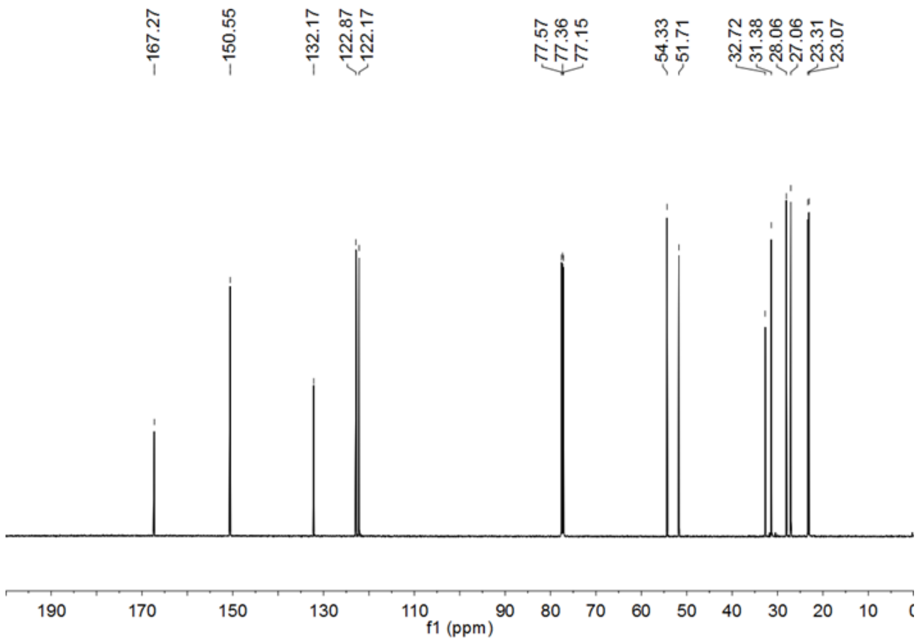
167.27
150.55
132.17
122.87
122.17
77.57
77.36
77.15
54.33
51.71
32.72
31.38
28.06
27.06
23.31
23.07
f1 (ppm)

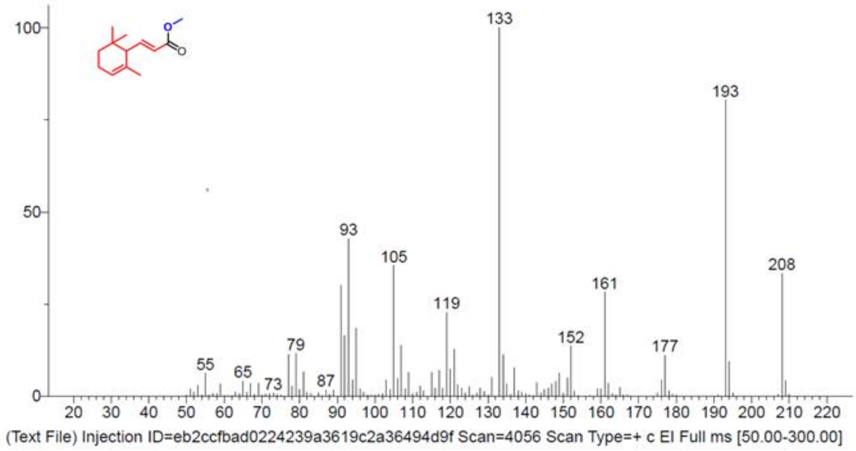
133
193
208
161
93
105
119
152
177
79
55
65
73
87
(Text File) Injection ID=eb2ccfbad0224239a3619c2a36494d9f Scan=4056 Scan Type=+ c EI Full ms [50.00-300.00]

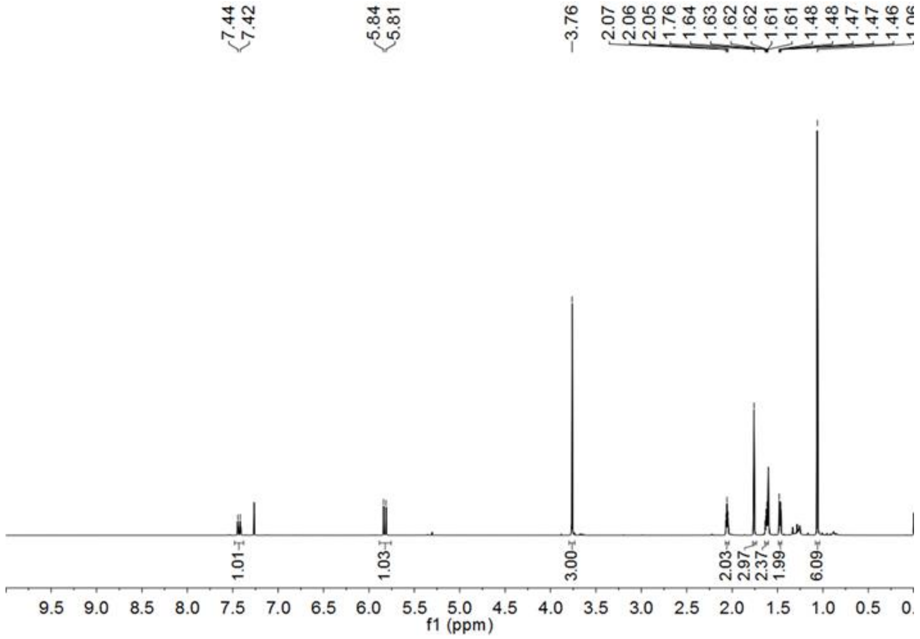
7.44
7.42
5.84
5.81
3.76
2.07
2.06
2.05
1.76
1.64
1.63
1.62
1.62
1.61
1.61
1.48
1.48
1.47
1.46
1.01
1.03
3.00
2.03
2.97
2.37
1.99
6.09
f1 (ppm)

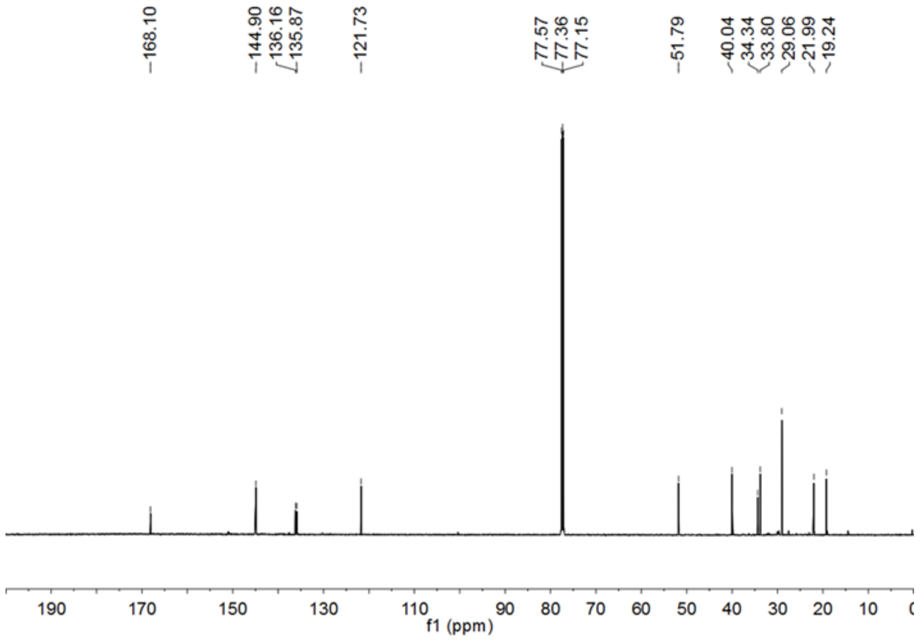
168.10
144.90
136.16
135.87
121.73
77.57
77.36
77.15
51.79
40.04
34.34
33.80
29.06
21.99
19.24
f1 (ppm)

Supplementary References

1. Luo, H. et. al. Cobalt nanoparticles-catalyzed widely applicable successive C-C bond cleavage in alcohols to access esters. Angew. Chem. Int. Ed. **59**, 19268-19274 (2020).
2. Liu, M. et. al. Aerobic oxidative cleavage and esterification of C(OH)-C bonds. *Chem* **6**, 3288-3296 (2020).
3. Ma, R.; He, L.-N.; Liu, A.-H.; Song, Q.-W. Cu(II)-catalyzed esterification reaction via aerobic oxidative cleavage of C(CO)-C(alkyl) bonds. *Chem. Commun.* **52**, 2145-2148 (2016).
4. Huang, X. et al. From ketones to esters by a Cu-catalyzed highly selective C(CO)-C(alkyl) bond cleavage: aerobic oxidation and oxygenation with air. *J. Am. Chem. Soc.* **136**, 14858-14865 (2014).
5. Zhang, L.; Bi, X.; Guan, X.; Li, X.; Liu, Q.; Barry, B.-D.; Liao, P. Chemoselective oxidative C(CO)-C(methyl) bond cleavage of methyl ketones to aldehydes catalyzed by CuI with molecular oxygen. *Angew. Chem., Int. Ed.*, **52**, 11303-11307 (2013).
6. Tang, C.; Jiao, N. Copper-catalyzed aerobic oxidative C-C bond cleavage for C-N bond formation: From ketones to amides. *Angew. Chem., Int. Ed.* **53**, 6528-6532 (2014).
7. Xu, B. et al. Copper-catalyzed aerobic conversion of the C=O bond of ketones to a C≡N bond using ammonium salts as the nitrogen source. *Chem. Commun.* **51**, 11264-11267 (2015).
8. Nakamura, R.; Obora, Y.; Ishii, Y. Selective oxidation of acetophenones bearing various functional groups to benzoic acid derivatives with molecular oxygen. *Adv. Synth. Catal.* **351**, 1677-1684 (2009).
9. Rammurthy, B.; Peraka, S.; Vasu, A.; Krishna Sai, G.; Divya Rohini, Y.; Narender, N. Metal-free catalytic esterification of aryl alkyl ketones with alcohols via free-radical mediated C(sp3)-H bond oxygenation. *Asian J. Org. Chem.* **10**, 594-601 (2021).
10. Higgins, S. D.; Thomas, C. B. Conversion of aromatic ketones into π-arylalkanoic acids. Oxidation by Thallium (III) and by halogens. *J. Chem. Soc., Perkin Trans.* **1**, 235-242 (1982).
11. Izawa, Y.; Ishiguro K.; Tomioka, H. Photoinduced alcoholysis of α,α,α-tribromoacetophenone to benzoylformate. *Bull. Chem. Soc. Jpn.* **56**, 1490-1496 (1983).
